# Relative configuration of micrograms of natural compounds using proton residual chemical shift anisotropy

**DOI:** 10.1038/s41467-020-18093-5

**Published:** 2020-09-01

**Authors:** Nilamoni Nath, Juan Carlos Fuentes-Monteverde, Dawrin Pech-Puch, Jaime Rodríguez, Carlos Jiménez, Markus Noll, Alexander Kreiter, Michael Reggelin, Armando Navarro-Vázquez, Christian Griesinger

**Affiliations:** 1 NMR based Structural Biology, MPI for Biophysical Chemistry, Am Fassberg 11, 37077 Göttingen, Germany; 2 Department of Chemistry, Gauhati University, Gopinath Bardoloi Nagar, Guwahati, 781014 India; 3 Centro de Investigacións Científicas Avanzadas (CICA), Departamento de Química, Facultade de Ciencias, Agrupación Estratéxica CICA-INIBIC, Universidade da Coruña, 15071 A Coruña, Spain; 4 Department of Chemistry, Technical University of Darmstadt, Alarich Weiss Straße 4, 64287 Darmstadt, Germany; 5 Departamento de Química Fundamental, CCEN, Universidade Federal de Pernambuco, Cidade Universitária, Recife, Brazil

**Keywords:** Marine chemistry, Solution-state NMR, Structure elucidation

## Abstract

3D molecular structure determination is a challenge for organic compounds or natural products available in minute amounts. Proton/proton and proton/carbon correlations yield the constitution. *J* couplings and NOEs oftentimes supported by one-bond ^1^H,^13^C residual dipolar couplings (RDCs) or by ^13^C residual chemical shift anisotropies (RCSAs) provide the relative configuration. However, these RDCs or carbon RCSAs rely on 1% natural abundance of ^13^C preventing their use for compounds available only in quantities of a few 10’s of µgs. By contrast, ^1^H RCSAs provide similar information on spatial orientation of structural moieties within a molecule, while using the abundant ^1^H spin. Herein, ^1^H RCSAs are accurately measured using constrained aligning gels or liquid crystals and applied to the 3D structural determination of molecules with varying complexities. Even more, deuterated alignment media allow the elucidation of the relative configuration of around 35 µg of a briarane compound isolated from *Briareum asbestinum*.

## Abstract (web summary)

Determination of 3D molecular structures remains challenging for natural products or organic compounds available in minute amounts. Here, the authors determine the structure of complex molecules, including few micrograms of briarane B-3 isolated from *Briareum asbestinums*, through measurement of ^1^H residual chemical shift anisotropy.

## Introduction

Given the enormous diversity of natural products, the elucidation of the 3D structure is the object of intense investigations by chemists^1,2^. 3D structure elucidation includes the determination of both relative and absolute configuration. It is well known that different stereoisomers generally have different biological and pharmacological properties. The number of chiral drugs which are clinically approved as a single enantiomer increases^3^, given that often one of the enantiomers is less active or has side effects. Therefore, basic science and pharmaceutical research rely on the correct determination of the relative and absolute configurations of novel compounds. A latest SciFinder search under the keyword ‘structure revision’ brings to the limelight that there were more than 1200 wrong structural reports between 1991 and 2016. There are even 39, 22, 35, and 46 wrong structural reports in 2016, 2017, 2018, and 2019, respectively. Obviously, information exists only about the known incorrect structures, while more incorrect structures might be published in the literature. Therefore, once the molecular constitution is known, the determination of the relative configuration of the stereogenic centers in natural products is essential^1^. However, current methods are time-consuming and error-prone. Total synthesis, the most laborious and time-consuming approach, is considered to be the gold standard to establish the configuration^1^. Yet, total synthesis of mefloquine and ulapualide A, for instance, provided incorrect absolute and relative configurations, respectively, in several reports^4–6^. While X-ray crystallography is a standard technique for crystalline compounds, nuclear magnetic resonance (NMR) is the method of choice to determine relative configuration when the sample does not crystallize. Conventionally, isotropic NMR restraints viz., nuclear Overhauser effect (NOE)^7^ and *J*-couplings^8^ are used to determine 3D structures of molecules. Yet, the combination of these two restraints proves to be insufficient for configuration determination in many cases, specifically when several bonds separate the chiral centers, and the molecule is flexible.

Anisotropic NMR has contributed powerful complementary restraints to NOEs and *J*-couplings^9,10^ which are residual dipolar couplings (RDCs) and residual chemical shift anisotropies (RCSAs)^11–14^. Although RDC applications date back to the sixties, there have been more reports in the past decade to determine the configuration of small molecules^12,15–19^. In contrast, ^13^C RCSA can be robustly measured, using conventional hardware, only since 2016^20–22^. So far, there are only a few reports of temperature-based RCSA measurements for biomolecules^23,24^. Since ^1^H has the highest gyromagnetic ratio and almost 100% natural abundance, ^1^H RCSAs should be measurable even for minute quantities of the compound to determine its configuration. Yet, ^1^H RCSAs have not been introduced for this purpose.

Anisotropic NMR has contributed powerful complementary restraints to isotropic chemical shifts, NOEs and *J*-couplings^9,10^ as implemented, for example, in the powerful DP4+ analysis tool^25^. Measurement of anisotropic NMR parameters requires partial ordering of the molecules in alignment media such as aligning gels and liquid crystals^9,26–33^. Although one-bond ^1^H-^13^C RDC measurement is the most common, the sensitivity of RDC measurement becomes problematic if the available sample is below a few hundred micrograms^34^. On the other hand, RCSA measurement had been difficult due to isotropic shift changes upon molecular alignment^35^. Recently, robust measurement of ^13^C RCSAs was reported by using the compression and stretching of PMMA gels or by using liquid crystals^20–22,35–37^. These RCSAs delivered the relative configuration for a number of molecules with several stereogenic centers by cross validation of the experimental RCSAs against the theoretical ones derived from all possible other relative configurations^22^. However, ^13^C RCSA measurements from 1D ^13^C spectra are impossible due to the low sensitivity of carbon if the available sample is below a few 10’s of micrograms at natural abundance.

As mentioned above, ^1^H RCSAs were not used to determine the configuration of small molecules although they are the most sensitive anisotropic NMR parameter available. Accurate ^1^H RCSA measurement holds the promise to the chemists that minute amounts will allow the determination of relative configurations for rigid and flexible molecules. Here, we introduce two independent tools to robustly measure ^1^H RCSAs, firstly, by using stretchable PMMA^22^ and poly-HEMA^31^ gels, and secondly, in the lyotropic liquid crystalline phase (LLC-phase) of a helically chiral polyarylacetylene (PPA-l-Val_dec_) prepared in chloroform^27,28^. It is noteworthy that polymer signals dominate and very often mask other signals when the sample amount is <100 μg. This problem does not occur when a deuterated gel such as PMMA-*d*_8_ is used, as demonstrated herein for 10 μg of strychnine (**1**), 40 μg of (−)-α-santonin, 45 μg of brucine, and 35 μg of a briarane diterpene (**3**), a natural compound whose configuration was not known before and can be corroborated by DP4+ analysis of the isotropic chemical shifts.

## Results

### ^1^H RCSA measurement of strychnine using PMMA gel

**Fig. 1:**
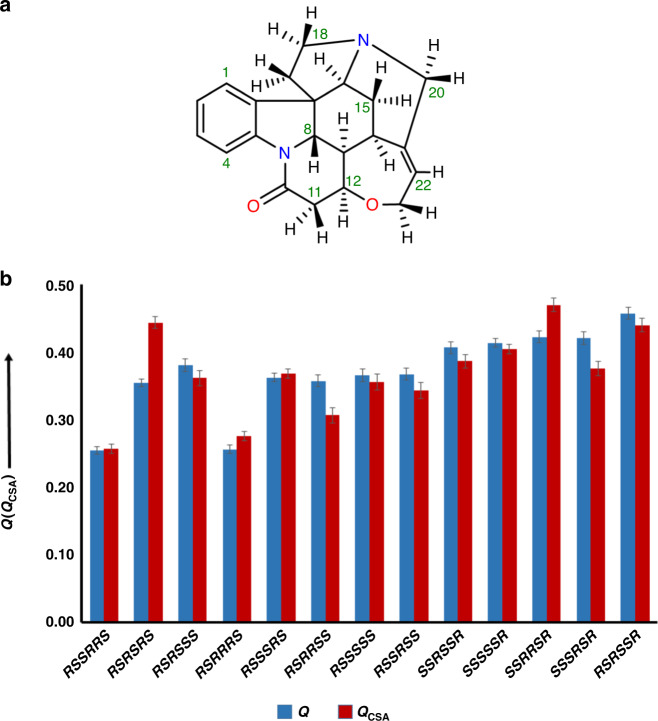
Configuration analysis of strychnine using ^1^H RCSA *Q/Q*_*CSA*_ factors in a PMMA gel. **a** Structure of strychnine (**1**). **b** Quality factors of ^1^H RCSAs derived from stretched PMMA gel of strychnine: the *Q*_*CSA*_ factors (red bar) for the two closest configurations *RSSRRS* and *RSRRRS* are 0.257 ± 0.006 and 0.275 ± 0.006, respectively. The *Q* factors are reported with the blue bars. The two configurations *RSSRRS* and *RSRRRS* were also the two with the lowest *Q* factors in the ^13^C RCSA analysis^21^. The error bars are standard deviations.

^1^H RCSA were measured from 8 mg of strychnine (**1**) (Fig. 1a) dissolved in CDCl_3_ utilizing the PPA-l-Val_dec_ based liquid crystal (Supplementary Note 7) and stretched PMMA gel. The latter was measured in a special NMR tube implementing maximum alignment by confining the gel within the inner diameter of 3.2 mm and minimum alignment by relaxing it to 4.2-mm inner diameter. Stretched PMMA gel derived ^1^H RCSAs ranged from 2.1 to 8.4 Hz measured in a TXI cryoprobe at 700 MHz. Equation (3) was used to extract the RCSAs. In order to analyze the different relative configuration, we derived an alignment tensor from the observed ^1^H RCSAs and density functional theory (DFT)-determined CSA tensors for each configuration using least-squares singular value decomposition (SVD) fitting using the MSpin-RDC program and quantifying the quality of the fit by *Q* factors. Strychnine is multicyclic and therefore only 13 (Fig. 1) out of the totally possible 32 configurations are energetically feasible and further analyzed^20,38^. Their chemical shift tensors were computed by DFT at B3LYP/6-311+G(2d,p) level using the IEF-PCM solvent continuum model in Gaussian 09^39^ with CHCl_3_ as solvent (Supplementary Note 3). The *R/S* nomenclature was used for the stereocenters at C7, C8, C12, C13, C14, and C16, respectively. In this order, *RSSRRS* represents the correct configuration (Fig. 1).

*Q* factors for the correct configuration *RSSRRS* and the next best incorrect configuration *RSRRRS* were almost identical (0.253 and 0.256). ^13^C RCSA with *Q* factors of 0.050 and 0.100, respectively, had identified these two configurations as best fitting^20^. CSA tensors for protons and specifically their axial component have a large variation, ranging for strychnine between 11.0 (H18b) and 2.9 (H8) ppm. Different from ^13^C axial components of chemical shift anisotropies which are large for olefinic and aromatic carbons and small for aliphatic, axial anisotropies for aromatic protons (H1, H2, H3, and H4: 10.2, 4.9, 4.4, and 9.3 ppm, respectively) and aliphatic protons (11H_b_, 13H, 20 H_a_, 15H_b_, and 18 H_a_ are 4.7, 2.9, 9.4, 10.2, and 11.0 ppm, respectively) cover the same range. Even diastereotopic protons have largely diverging axial components of the CSA, 18 H_a_: 11.0 ppm, 18H_b_: 5.7 ppm. Similar to the approach taken for the evaluation of carbon RCSAs which vary a lot^20^, *Q*_CSA_ [Eq. (5)] scales the RCSA deviation of experimental and theoretical of each nucleus with the axial value of the CSA. With this definition, each RCSA is more equally weighted. Indeed, *Q*_CSA_ yields: *Q*_CSA_ = 0.257 ± 0.006 for the correct *RSSRRS* and 0.275 ± 0.006 for the next best incorrect *RSRRRS* configuration, distinguishing better. *Q* factor errors were calculated by the Monte Carlo procedure (Supplementary Notes 5 and 14). These errors are expressed as standard deviations throughout the paper and Supplementary Information. Similar to ^13^C RCSAs, ^1^H RCSAs furnish the lowest *Q* and *Q*_CSA_ values for the correct configuration^21^. The RCSA *Q* and *Q*_*CSA*_ factors computed for the thirteen possible configurations are listed with a bar diagram in Fig. 1b.

### ^1^H RCSA of 10-μg strychnine in deuterated PMMA gel

**Fig. 2:**
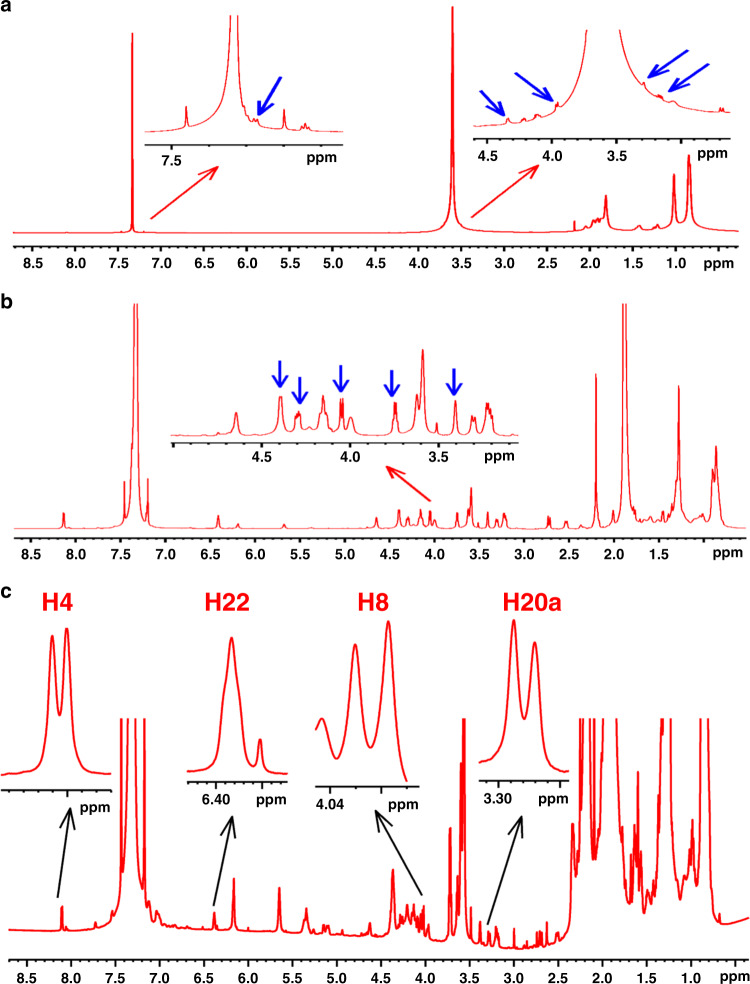
1D ^1^H NMR spectra of microgram amount of strychnine in gels. **a** 1D ^1^H NMR spectrum of 300 μg strychnine in protonated PMMA gel. The spectrum was acquired with 256 scans. Note that only few peaks from the analyte are visible (indicated by the blue arrows) and many signals are masked by the polymer signals. **b** 1D ^1^H NMR spectrum of 80-μg strychnine in deuterated PMMA gel. The spectrum was acquired with 800 scans. Blue arrows on the inset in (**b**) highlight some strychnine resonances that become visible due to the removal of proton signals of the polymer. Both spectra were recorded in a Bruker 800 MHz NMR spectrometer equipped with a TCI cryoprobe. Stretching devices with an inner diameter of 4.0 mm for minimum and 3.2 mm for maximum alignment were used. **c** 1D ^1^H NMR spectrum of 10-μg strychnine acquired with 8192 scans in deuterated PMMA gel under maximum alignment condition. For clarity, expansions for some of the proton signals (H4, H22, H8, and H20a) are also shown.

In the previous subchapter, 8 mg of strychnine were used. Due to the high sensitivity of proton detection, a few micrograms of the sample embedded in a PMMA gel should be sufficient to measure ^1^H RCSAs. However, given the relative concentration of the gel and the solute, the gel resonances are by orders of magnitude larger than those of the solute and cannot be suppressed with *T*_2_ filters or solvent suppression. Therefore, the proton resonances of the gel^40^ were removed by using commercially available and affordable deuterated monomers (Supplementary Fig. 1 where the prominent signals that are usually observed in the protonated gels are now nearly completely absent). The cross-linker could not be obtained in deuterated form and is much less abundant in the gel than the polymerized monomer. The 1D ^1^H NMR spectrum of 300 μg of strychnine aligned in stretched protonated PMMA gel (Fig. 2a) is compared to that of 80-μg strychnine aligned in deuterated PMMA gel (PMMA-*d*_8_) (Supplementary Note 1: sample preparation) (Fig. 2b) and shows the excellent suppression of the gel peaks. The deuterated gels cost marginally more than the protonated as discussed in the Supplementary Note 1: sample preparation. Given that in PMMA-*d*_8_, the polymer signals are suppressed completely 20 ^1^H RCSAs of strychnine could be extracted (Supplementary Table 18) while this is impossible in the protonated gel due to overlap of strychnine protons with the very intense polymer signals. Obviously, the smaller the gel amount, the less residual signal is observed from the protonated PMMA gel which is optimal in a 1.7 mm compression device (see Supplementary Fig. 3). Using the stretching device with 4.2- and 3.2-mm inner diameters RCSA ranged from −0.8 to 3.4 Hz at 800 MHz using a TCI cryoprobe. SVD fitting of the ^1^H RCSAs to a single tensor yielded *Q* (*Q*_CSA_) values of 0.208 (0.231) ± 0.015 (0.022) for the correct *RSSRRS* configuration and 0.271 (0.305) ± 0.015 (0.020) for the next best (incorrect) *RSRRRS* configuration. Even higher *Q* (*Q*_CSA_) factors were observed for the other 11 configurations (see Supplementary Tables 7 and 8).

After the successful measurement of a 80 μg sample with the deuteration strategy, we further prepared an aligned sample of only 10 μg of strychnine (**1**) in PMMA-*d*_8_ gel. A micro stretching device with 2.2- and 1.8-mm inner diameters (Supplementary Note 2) implemented maximum and minimum alignment on a 700 MHz NMR spectrometer. Sufficient signal-to-noise ratios were achieved after 8192 scans. Twelve ^1^H RCSAs could be measured (Supplementary Table 19) as compared to 20 RCSAs collected for the 80 μg sample, since solute signals overlapped with gel signals. SVD fitting of the ^1^H RCSAs to a single tensor yielded *Q*(*Q*_CSA_) values of 0.421 (0.455) ± 0.035 (0.056) for *RSSRRS* and 0.666 (0.676) ± 0.022 (0.021) for the next best *RSRRRS* configuration. The other 11 configurations have even higher *Q* (*Q*_CSA_) factors.

### Microgram level ^1^H RCSA analysis of brucine and santonin

To demonstrate the general discriminatory capability of ^1^H RCSA data, they were recorded at a lower field of 600 MHz for two more molecules, (−)-α-santonin and brucine using a few 10 s of μg. The (−)-α-santonin sample amount used was 40 µg and ^1^H RCSAs were measured utilizing PMMA-*d*_8_ gel inserted in a micro stretching device. For (−)-α-santonin, it should be mentioned that the configuration determination was not possible using one-bond ^1^*D*_CH_ RDCs. However, the ^1^H RCSA data (Supplementary Table 25) renders excellent discrimination between the correct and incorrect configurations. The correct *SSSS* configuration furnished the lowest *Q* (*Q*_CSA_) of 0.262 (0.241) ± 0.044 (0.049) while the closest incorrect *RRRS* configuration furnished 0.474 (0.449) ± 0.041 (0.061) (see Supplementary Note 12). Similarly, we measured the ^1^H RCSAs of 45 µg brucine sample at a 600 MHz spectrometer equipped with a QXI cryoprobe (Supplementary Table 26). Considering that PMMA-*d*_8_ gels are reusable at least for three samples, the gel used for brucine was used before for (−)-α-santonin. The analysis of the ^1^H RCSA data provided *Q* (*Q*_CSA_) factors of 0.157 (0.136) ± 0.028 (0.025) for correct configuration and 0.205 (0.325) ± 0.030 (0.054) for the closest incorrect *RSSRRS* configuration (see Supplementary Note 13). It is interesting to note that the *Q*_CSA_ for the correct configuration is smaller than *Q* while it is opposite for the incorrect one. This can be attributed to H14 whose axial CSA for the correct configuration is 6.7 ppm, but for the incorrect one is 1.9 ppm. Therefore, as compared with *Q, Q*_CSA_ emphasizes the RCSA of H14 in the wrong configuration increasing *Q*_CSA_, while for the correct configuration *Q*_CSA_ is reduced by the large CSA of H14.

### Configuration of estrone in poly-HEMA gel

Measurement of ^1^H RCSAs of strychnine in the LLC-phase of PPA-l-Val_dec_ in CDCl_3_ allows determining the relative configuration as well (see Supplementary Note 7). Similarly, ^1^H RCSAs of estrone measured in poly-HEMA gel swollen in DMSO-*d*_6_ allow the determination of the relative configuration (see Supplementary Note 8). It will be important in the future to make fully deuterated polyaryacetylenes with amino acid side chains and fully deuterated poly-HEMA accessible at an affordable price.

### Relative configuration of flexible molecule—retrorsine

**Fig. 3:**
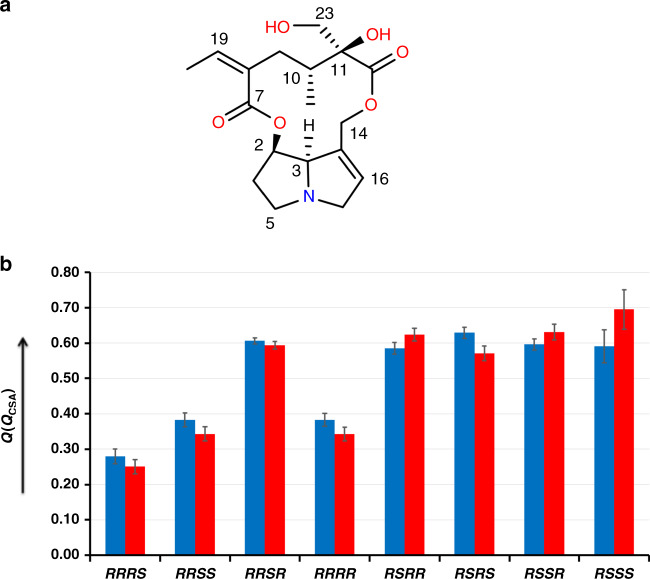
*Q/Q*_*CSA*_ factors of retrorsine. **a** Structure of retrorsine (**2**). **b** The *Q* factors (blue bar) and *Q*_*CSA*_ factors (red bar) for the eight possible relative configurations for the retrorsine. The error bars are standard deviations.

The test molecules investigated so far were rigid molecules. To learn more about the limits of the ^1^H RCSA method, we next applied it to 1 mg of the flexible molecule retrorsine (**2**) (Fig. 3a, b). Using only ^1^*D*_CH_ RDCs it was not possible to assign the correct configuration of retrorsine^41^ mainly due to lack of information about the quaternary chiral center at C11. However, the configuration analysis of retrorsine was accomplished using ^13^C RCSAs^20^. For configuration analysis of retrorsine, the complete conformational ensembles for each possible configuration that are compatible with the NMR data need to be considered. The conformers that are feasible energetically (Supplementary Table 4) were obtained with molecular modeling calculations using the force field MMFF94 in the Macromodel program^42^. The experimental RCSAs (Supplementary Table 23) were fitted to a single tensor. One mg of the sample was aligned in the gel by using a stretching device and RCSAs were measured at a proton frequency of 800 MHz. The RCSA analysis provided *Q* (*Q*_CSA_) factors of 0.280 (0.251) ± 0.021 (0.020) and 0.347 (0.297) ± 0.019 (0.020) for the *RRRS* and *RRRR* configurations, respectively. Since X-ray crystallography also confirms the *RRRS* configuration of the molecule^43^, it nicely shows that the RCSA method indeed discriminates correctly. It may be noted here that these two configurations are also the closest ones when ^13^C RCSA analysis was performed with *Q* factors of 0.184 and 0.216, respectively^20^. Note that the *Q* factor difference and ratio is larger, i.e., discrimination between the configurations is more obvious using ^1^H RCSA.

### Absolute configuration of 35-μg briarane B-3 with unknown stereochemistry

**Fig. 4:**
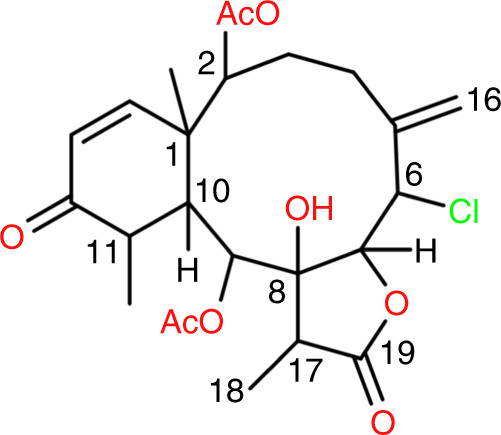
Constitution of the diterpene briarane B-3. The constitutions of briarane B-3 (**3**) is shown.

Finally, we used ^1^H RCSA data for the configuration analysis of a briarane diterpenoid, compound **3**, which was isolated from the gorgonian *Briareum asbestinum* (see below) collected in the waters off the Yucatan Peninsula (Mexico). The constitution of **3** (Fig. 4), deduced by standard NMR and MS analysis (Supplementary Note 9), resulted to be the same as that of briarane B-3, which was reported by Harvell et al. in 1993 without spectroscopic support^44^.

The large number of stereogenic centers present in **3**, its conformational flexibility and the lack of reported data supporting its configuration, lead us to consider this compound as a good test of the ^1^H NMR RCSA methodology in establishing relative configuration. Briaranes have a wide range of biological activities such as anti-inflammatory, antibacterial, cytotoxic, etc. For instance, solenolide E, structurally related to **3**, exhibits a very pronounced antiviral activity against rhinovirus, herpes and Ann Arbor viruses, displays anti-inflammatory activity and is an inhibitor of a cyclooxygenase enzyme^45^.

**Fig. 5:**
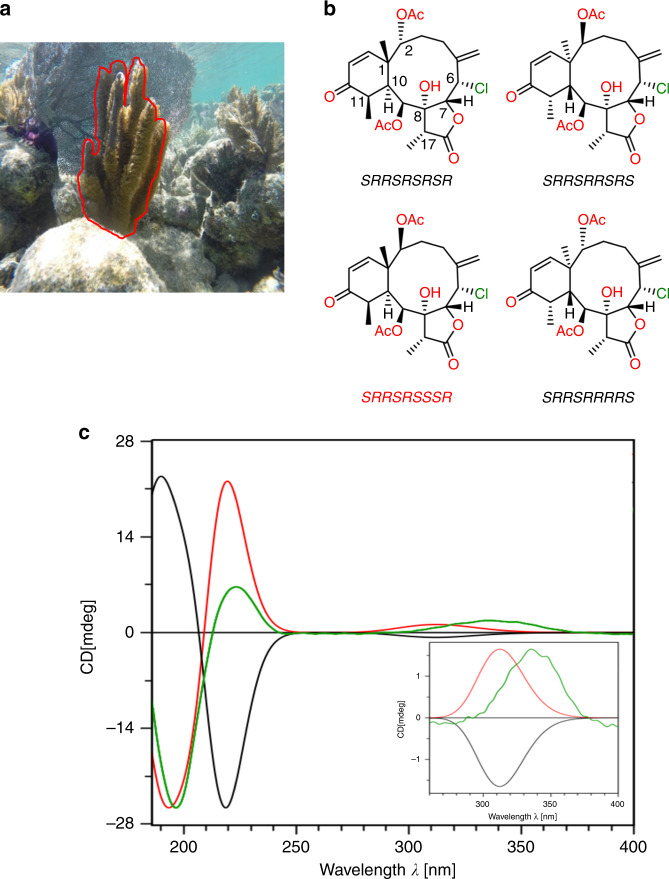
Absolute configuration of 35-μg briarane B-3. **a** Marine gorgonian *Briareum asbestinum* (continuous red line) collected in the waters off the Yucatan Peninsula in Mexico, from which briarane B-3 (**3**) was isolated. **b** The four possible diastereomers for the structure of **3** of which the *SRRSRSSSR* (**3c**) (red) is found to be the correct one. **c** Calculated ECD spectra of *SRRSRSSSR* (red line) and enantiomer *RSSRSRRRS* (black line) versus experimental ECD spectrum (green line) of **3**. Note that the ordering of the stereocenters is C6, C7, C8, C9, C17, C1, C2, C10, C11.

The stereochemical analysis of **3** started with conventional NMR using *J*-couplings and NOEs which allows the determination of the configuration at C6, C7, C8, C9, and C17 as *SRRSR* or *RSSRS* using only 35 μg for these isotropic spectra. Further NMR analysis (see Supplementary Note 10) leave the possible configurations at C1, C2, C10, and C11 to four, i.e. *SRSR*, *SSSR*, *RRRS*, and *RSRS* (Fig. 5b). There are then four possible pairs of enantiomers: (keeping the order C6, C7, C8, C9, C17, C1, C2, C10, and C11): *SRRSRSRSR*/enantiomer (**3a**), *SRRSRRSRS*/enantiomer (**3b**), *SRRSRSSSR*/enantiomer (**3c**), and finally *SRRSRRRRS*/enantiomer (**3d**) showed in Fig. 4b.

To determine the relative configuration of these stereogenic centers, ^1^H RCSAs of **3** (Supplementary Table 24) were measured in PMMA-*d*_8_ gel using a micro stretching device with 2.2- and 1.8-mm inner diameters. The sample amount continued to be 35 μg and experimental ^1^H RCSAs range from 1.8 to 3.2 Hz at a ^1^H frequency of 800 MHz. Compound **3** is a flexible molecule, therefore we fitted the ^1^H RCSA data to multiple conformers using a single tensor for the four relative configurations. The *SRRSRSSSR* configuration (**3c**) furnished the lowest *Q(Q*_*CSA*_*)* factor of 0.176 (0.219) ± 0.036 (0.047), while the other configurations had *Q(Q*_*CSA*_*)* factor of 0.284 (0.308) ± 0.027 (0.031), 0.315 (0.376) ± 0.032 (0.042), and 0.315 (0.376) ± 0.032 (0.042) for *SRRSRRRRS* (**3d**), *SRRSRRSRS* (**3b**), and *SRRSRSRSR* (**3a**), respectively. Given the standard deviations, the *Q*(*Q*_CSA_) factor difference of 0.108 (0.089) between *SRRSRSSSR* (**3c**) and other three diastereomers shown in Fig. 5b established unequivocally the former as the correct relative configuration. This result is in agreement with the DP4+ analysis where we used the conformations derived from ^1^H RCSA analysis (see Supplementary Note 15). With the relative configuration already established, we computed the ECD spectrum of the enantiomers: *SRRSRSSSR* and *RSSRSRRRS* by using time-dependent DFT at the CAM-B3LYP/6-311++G(2d,p) level. The calculated ECD spectra for some of the RCSA determined conformers in the ensemble are significantly different (Supplementary Note 11 and Fig. 25). Therefore, we used a linear combination of the conformations with the populations (Supplementary Table 16) determined from the RCSA analysis to simulate the theoretical ECD curve. The ECD spectrum calculated for *SRRSRSSSR* agrees very well with the experimental one, which unambiguously assigns the absolute configuration. As expected, the calculated ECD spectrum of the enantiomer *RSSRSRRRS* is inverted (Fig. 5c).

Although the ^1^H RCSA methodology works very well for flexible retrorsine and briarane B-3, it may have certain limitations if the molecule is too flexible. The flexibility problem is however not new to ^1^H RCSAs but applies similarly to ^13^C RCSAs or RDCs. Flexibility is less of a problem when there are more NMR parameters. Therefore, we expect that the use of ^1^H RCSAs in addition to ^13^C RCSAs and RDCs will be more powerful than using one of them to solve very flexible molecules.

## Discussion

We reported that ^1^H RCSAs as highly sensitive anisotropic NMR observables can be robustly measured in LLC-phases of a helically chiral polyarylacetylene in chloroform and in DMSO swollen chemically cross-linked polymer gels. ^1^H RCSAs are powerful parameters as they complement conventional *J-*couplings, and NOEs, without the necessity to resort to one bond and long range ^1^H-^13^C RDCs or ^13^C RCSAs. For tiny amounts of analytes, we showed that deuterated gels provide clean spectra down to 10 μg for strychnine, 35 μg for the unknown briarane B-3 (**3**), 45 μg for brucine and 40 μg for α-santonin.

By analysis of these examples of known molecules as well as one unknown molecule, both rigid and flexible, we demonstrated that ^1^H RCSAs can be successfully utilized to determine the correct relative configuration using *Q* or *Q*_CSA_ values. CSA tensor DFT calculations are sufficiently robust such that the result does not depend on the basis sets, DFT method or solvent. In addition, chemists not well trained in NMR will find it more appealing to use RCSAs than RDCs since RCSAs can be easily read from the 1D ^1^H or ^13^C NMR/spectra while RDC measurements require some training with 2D spectroscopy. The analysis tools are available in MSpin-RDC software and the CSA tensor calculations can be done with the Gaussian program package. Therefore, measurement of ^1^H RCSA, which requires only 10 s of micrograms of analyte, will be valuable for the structural analysis of synthetic and natural products that are hitherto not solvable due to their limited availability.

## Methods

### Measurement of RCSAs in liquid crystal

For the liquid crystalline sample, molecular alignment varies with temperature. The temperature dependence of the chemical shift is small for isotropic and anisotropic solutions and relatively constant over a range of 10 °C. Therefore, ^1^H RCSAs can be measured by varying the alignment of the sample inside a 5 mm normal NMR tube at certain temperatures. One way to measure ^1^H RCSAs is to record 1D ^1^H NMR spectra in the anisotropic and isotropic phases. The ^1^H chemical shift differences between these phases provide RCSAs that are not corrected for isotropic contributions. The isotropic contribution can be corrected by calculating the temperature-induced isotropic shift using a second 1D ^1^H NMR spectrum recorded at another temperature in which the phase is isotropic. If the liquid crystal is anisotropic at 300 K and isotropic at, let us say, 305 and 310 K, then the RCSA of nuclei ‘*i*’ is determined from the following equation,

1 $${\Delta}{\mathrm{RCSA}}_i\,=\,\left( {\delta _i^{300{\mathrm{K}}}\,-\,\delta _i^{305{\mathrm{K}}}} \right)\,-\,\left( {\delta _i^{305{\mathrm{K}}}\,-\,\delta _i^{310{\mathrm{K}}}} \right)\,=\,\delta _i^{300{\mathrm{K}}}\,-\,2\delta _i^{305{\mathrm{K}}}\,+\,\delta _i^{310{\mathrm{K}}},$$

where ‘*i*’ is the *i*th ^1^H and *δ*_i_ is the chemical shift of ‘*i*’. This equation assumes a linear temperature dependence of the isotropic shift between 300 and 310 K such that the temperature independent anisotropic shift can be separated from the temperature dependent isotropic shift.

In case, two temperatures yielding isotropic phases cannot be accessed within the temperature range of the probe, the RCSA can also be measured correctly by measuring the anisotropic and isotropic spectra at two temperatures e.g., 300 and 315 K using the following equations.

2 $${\Delta}{\mathrm{RCSA}}_i\,=\,{\mathrm{(}}\delta _i^{300{\mathrm{K}}}\,-\,\delta _{{\mathrm{ref}}}^{300{\mathrm{K}}}{\mathrm{)}}_{{\mathrm{aniso}}}\,-\,{\mathrm{(}}\delta _i^{315{\mathrm{K}}}\,-\,\delta _{{\mathrm{ref}}}^{315{\mathrm{K}}}{\mathrm{)}}_{{\mathrm{iso}}},$$

where, *δ*_ref_ is the chemical shift for any chosen reference nucleus chosen from the analyte. Different alignment strengths are achieved by a small variation of the sample temperature such that this method does not need any special tube or piston in the tube. Therefore, the measurement can be performed in 1.7, 3 mm, or in standard 5 mm NMR tubes unlike with the constrained gels that require special devices.

For the polyacetylene based liquid crystal used in the present work, the isotropic conditions were not reached in the range of 300–320 K. Due to temperature limitations of the probe, isotropic conditions were not checked at temperatures >325 K. Therefore, Eq. (2) was used to extract the ^1^H RCSAs. Equation (1) can be used for other liquid crystals that show isotropic behavior at two different temperatures above the liquid crystal clearing point or by diluting the sample with a few % of solvent.

### Measurement of RCSAs in stretchable gels

For stretchable gels, elongating the gel through radial mechanical force induces molecular alignment. During this process, the analyte concentration remains constant and hence, no correction due to isotropic chemical shift changes is required. For such a gel, the RCSA of a nucleus ‘*i*’ is derived from the following equation^20^.

3 $${\Delta}{\mathrm{RCSA}}_{i}\,=\,\left(\delta _i\,-\,\delta _{\mathrm{ref}} \right)^{\max}\,-\,\left(\delta _i\,-\,\delta _{\mathrm{ref}} \right)^{\min}.$$

Here, one of the nuclei is taken as reference atom and RCSA is measured as a chemical shift difference between the maximum (max) and minimum (min) alignment conditions.

### Chemical shift anisotropy calculation

The anisotropic distribution of orientations of the compound under alignment conditions is described by the alignment tensor *Â* that contains five independent elements and therefore requires minimally five linearly independent RCSAs^46,47^. Furthermore, the chemical shift tensors necessary for RCSA analyses can be obtained at low computational cost by using GIAO-based DFT calculations in Gaussian 09^39,48^. DFT methods are very powerful and reliable in determining the various NMR parameters from the optimized geometries. In practice, they can be calculated in parallel in a time frame that is usually chosen for the measurement of experimental data, thereby speeding up the structure elucidation process especially when many stereogenic centers need to be defined (Supplementary Note 4). The proton shifts can be calculated with enough accuracy by using DFT methods with a larger basis set or by using Møller–Plesset perturbation theory^49^. Accounting for the impact of the computational level on the computed ^1^H CSAs anisotropies a standard deviation of the axial component of the ^1^H CSAs [i.e., *σ*_33_ − (*σ*_22_ + *σ*_11_)/2] as low as 0.19 ppm was obtained here after variation of solvent models, basis sets and DFT methods (see Supplementary Table 6)^49^. We also took this CSA variation as error contributing to the back-calculated RCSAs and used this in the Monte Carlo analysis when calculating the standard deviations of *Q* and *Q*_CSA_ for the different configurations. Different from carbon atoms whose chemical shifts are only marginally influenced by the solvent, the proton CSA values are much largely influenced. In view of this, we performed calculations in two steps. First, proton CSAs were computed with different DFT functional and MP2 level with various medium to larger size basis sets. This step enables the selection of the best theoretical model. In the second step, the impact of the different solvent models on the CSA values was tested. Among the different basis set used for the calculation, 3–21G is the minimal basis set that provided a correct configuration assignment for each solvent. With higher basis sets, the results for the assignment does not change, although it reduces the *Q* factors (Supplementary Table 10). Note that for methyl group, all three-proton RCSA tensors are calculated and then averaged.

### Quality factors

The experimentally measured RCSAs are fit to a single alignment tensor using SVD as implemented in the MSpin-RDC program^50^. The quality of the fit is represented by the following quality factors:

4 $$Q\,=\,\sqrt {\frac{{{\sum} ({\rm{RCSA}}_{{\mathrm{exp}}}\,-\,{\rm{RCSA}}_{\rm{cal}})^2}}{{{\sum} {\rm{RCSA}_{{\mathrm{exp}}}^2} }}}$$

and

5 $$Q_{\rm{CSA}}\,=\,\sqrt {\frac{{{\sum} {\left[ {( {\rm{RCSA}_{{\mathrm{exp}}}\,-\,RCSA_{cal}} ){\mathrm{/}}{\rm{CSA}}^{2}} \right]} }}{{{\sum} {( {\rm{RCSA}_{{\mathrm{exp}}}{\mathrm{/}}CSA} )^2} }}}$$

in which, CSA, the axial anisotropy of the tensor equals σ_33_ − (σ_22_ + σ_11_)/2 and the chemical shielding Eigenvalues *σ*_ii_ are obtained from DFT. Ideally, for the correct configuration, the *Q* factor should be 0. Analysis of error propagation is described in the Supplementary Note 14. For flexible molecules, conformers were obtained from force field calculations using MMFF94^42^. Performing RCSA data analysis under single tensor approximation, populations are fit with the MSpin-RDC software along with alignment tensor components.

## Supplementary information

Supplementary Information

Description of Additional Supplementary Files

Supplementary Data 1

## Data Availability

All data used in this paper are available from the corresponding author upon request. All the data used in the paper, DFT structural co-ordinates along with the CSA tensors and other reporting summaries for this article is available as a Supplementary data 1 file. All the bar plots including the frequency polygons of error analysis are provided in a excel file as a Source Data File. Source data are provided with this paper.

## Source data

Source Data

## Code availability

All codes that are not commercially available will be provided by the corresponding author upon request. Source data are provided with this paper.
