# Author Correction: Relative configuration of micrograms of natural compounds using proton residual chemical shift anisotropy

**DOI:** 10.1038/s41467-020-18927-2

**Published:** 2020-09-29

**Authors:** Nilamoni Nath, Juan Carlos Fuentes-Monteverde, Dawrin Pech-Puch, Jaime Rodríguez, Carlos Jiménez, Markus Noll, Alexander Kreiter, Michael Reggelin, Armando Navarro-Vázquez, Christian Griesinger

**Affiliations:** 1 NMR based Structural Biology, MPI for Biophysical Chemistry, Am Fassberg 11, 37077 Göttingen, Germany; 2 Department of Chemistry, Gauhati University, Gopinath Bardoloi Nagar, Guwahati, 781014 India; 3 Centro de Investigacións Cientıfí cas Avanzadas (CICA), Departamento de Quıḿ ica, Facultade de Ciencias, Agrupación Estratéxica CICA-INIBIC, Universidade da Coruña, 15071 A, Coruña, Spain; 4 Department of Chemistry, Technical University of Darmstadt, Alarich Weiss Straße 4, 64287 Darmstadt, Germany; 5 Departamento de Química Fundamental, CCEN, Universidade Federal de Pernambuco, Cidade Universitária, Recife, Brazil

**Keywords:** Marine chemistry, Solution-state NMR, Structure elucidation

Correction to: *Nature Communications* 10.1038/s41467-020-18093-5, published online 01 September 2020.

The original version of the Supplementary Information associated with this Article contained errors in Supplementary Fig. 9a and on page 7. In both cases, chemical formula of the liquid crystalline medium was drawn incorrectly. The HTML has been updated to include a corrected version of the Supplementary Information; the original incorrect versions of these Figs. can be found as Supplementary Information associated with this Correction.

## Supplementary information

Supplementary Information

