# Supplementary material for: Relative configuration of micrograms of natural compounds using proton residual chemical shift anisotropy
Source: Nat Commun. 2020 Sep 1;11:4372. doi: 10.1038/s41467-020-18093-5 (PMC7463026; doi:10.1038/s41467-020-18093-5)
Supplement: Supplementary file 1 — Supplementary Information [file 41467_2020_18093_MOESM1_ESM.pdf]

Supplementary Information for manuscript

Relative configuration of micrograms of natural compounds using proton residual chemical shift anisotropy

By Nath, Fuentes-Monteverde *et al.*

Table of Contents

Supplementary Note 1: Sample preparation…………………………………………………………5

Supplementary Note 2: Micro Stretching Device (MSD)..10

Supplementary Note 3: Computational methods………………………………………………12

Supplementary Note 4: Chemical shift tensor calculation.……..………………………………15

Supplementary Note 5: Calculation of standard deviation associated with ^{1}H RCSAs…………...18

Supplementary Note 6: Comparison of Q and Q_{CSA} factors at the different levels of theory.........21

Supplementary Note 7: Relative configuration analysis of strychnine in liquid crystal…………25

Supplementary Note 8: Relative configuration analysis of estrone………………………………27

Supplementary Note 9: NMR data and analysis of briarane B-3……………………...…….……29

Supplementary Note 10: Relative configuration analysis of briarane B-3…………………….……41

Supplementary Note 11: ECD measurement……………………………………………….......43

Supplementary Note 12: 40 µg ^{1}H RCSA analysis of santonin………………………………… 48

Supplementary Note 13: 45 µg ^{1}H RCSA analysis of brucine……………………………………53

Supplementary Note 14: Monte Carlo analysis……………………………………………..…...58

Supplementary Note 15: DP4+ analysis…..65

Supplementary Note 16: Experimental data for molecules investigated.………………….………66

Supplementary Note 17: Alignment Tensor parameters from SVD……………………………...73

List of Figures

Supplementary Figure 1: Overlay of 1D ^{1}H NMR spectra of stretchable………………………...6

Supplementary Figure 2: (a): 3 mm tube attached to a modified teflon-made-cap……………….9

Supplementary Figure 3: 1D ^{1}H NMR spectra of 70 µg strychnine sample in micro…………...10

Supplementary Figure 4: Hilgenberg´s micro stretching device……………………………...12

Supplementary Figure 5: The 13 sterically feasible diastereomers of strychnine ……………......13

Supplementary Figure 6: Experimental ^{1}H chemical shifts (δ_{exp})………………………………17

Supplementary Figure 7: Workflow for calculating the standard………………………………18

Supplementary Figure 8: Variation of *RSSRRS*-strychnine ^{1}H $CSA_{b,i,ax}$……………………………19

Supplementary Figure 9: A) Workflow for measuring ^{1}H RCSA using as alignment media..….26

Supplementary Figure 10: The Q factors (blue bar) and Q_{CSA} factors (red bar) ………………..28

Supplementary Figure 11-14: Isotropic NMR spectra of 2 mg of briarane B-3……………...31-33

Supplementary Figure 15: A) Key HMBC correlations, B) Key NOESY correlations…………34

Supplementary Figure 16-22: Isotropic NMR spectra of 35 μg sample of briarane B-3…….35-41
Supplementary Figure 23: 1D ^{1}H spectrum for a 35 μg sample of briarane B-3…………………42
Supplementary Figure 24. Bar plot for the briarane B-3 ^{1}H RCSA fitting………………………44
Supplementary Figure 25: Calculated ECD (red line) for the different conformers……………...46
Supplementary Figure 26: Conformers of 1*S*,2*S*,6*S*,7*R*,8*R*,9*S*,10*S*,11*R*,17*R*-briarane B-3………47
Supplementary Figure 27: Conformers of 1*R*,2*R*,6*R*,7*S*,8*S*,9*R*,10*R*,11*S*,17*S*-briarane B-3………48
Supplementary Figure 28-33: Figures for the ^{1}H RCSA analysis of santonin ……..………49-53
Supplementary Figure 34-39: Figures for the ^{1}H RCSA analysis of brucine ……….………54-57
Supplementary Figure 40: Monte Carlo analysis for the ^{1}H RCSA analysis for …………………57
Supplementary Figure 41: Monte Carlo analysis for the ^{1}H RCSA analysis for the…………….59
Supplementary Figure 42: Q and Q_{CSA} frequency polygons of *RSSRRS*-strychnine……………...60
Supplementary Figure 43: Q and Q_{CSA} frequency polygons of *RSSRRS* -strychnine…………….61
Supplementary Figure 44: Q and Q_{CSA} frequency polygons of estrone (blue line) and…………62
Supplementary Figure 45: Monte Carlo simulation of *RRSS*-retrorsine and *RRRS*-retrorsine……63
Supplementary Figure 46: Monte Carlo simulation of $Q(Q_{CSA})$………………………………………63
Supplementary Figure 47: Monte Carlo simulation of $Q(Q_{CSA})$……………………..……………64

List of Tables

Supplementary Table 1: Chemicals used for gel preparation of the fully…………………………6
Supplementary Table 2: Amount of chemicals used on liquid crystal sample preparation……….8
Supplementary Table 3: Gels composition and quadrupolar splitting of the gel prepared……….11
Supplementary Table 4: Number of conformers of the different…………………….……………14
Supplementary Table 5: Proton chemical shift of strychnine……………………………………16
Supplementary Table 6: Axial part of ^{1}H CSA for the different protons…………………………20
Supplementary Table 7: Bar plot showing the Q factors (blue bar) and Q_{CSA}……………………22
Supplementary Table 8: Q_{CSA} factors of strychnine (80 μg sample) at DFT levels ……………...23
Supplementary Table 9: Bar plot showing the Q factors (blue bar) and Q_{CSA} …………………...24
Supplementary Table 10: Q factor calculated for estrone and 13-*epi*-estrone ……………..…...27
Supplementary Table 11: Alignment tensor´s GDO, standard deviation ………………………28
Supplementary Table 12: Alignment tensor´s GDO, standard deviation ……….…...................29
Supplementary Table 13: Alignment tensor´s GDO, standard deviation ………………………29
Supplementary Table 14: ^{1}H and ^{13}C chemical shift assignment of briarane B-3………………30

Supplementary Table 15: ^{1}H RCSA data for the fitting of briarane B-3……………………….43
Supplementary Table 16: RCSA determined populations for different……………………....45
Supplementary Table 17. Proton residual chemical shift anisotropies error (Hz) ……………...57
Supplementary Table 18: Experimental ^{1}H ΔRCSA values for 80 μg strychnine………………66
Supplementary Table 19: Experimental ^{1}H ΔRCSA values for 10 μg strychnine………………67
Supplementary Table 20: Experimental ^{1}H ΔRCSA values for strychnine in liquid crystal……67
Supplementary Table 21: Experimental ^{1}H ΔRCSA values for 8 mg strychnine……………….68
Supplementary Table 22: Experimental ^{1}H ΔRCSA values for 3 mg estrone…………………..69
Supplementary Table 23: Experimental ^{1}H ΔRCSA values for 1 mg retrorsine in gel…………70
Supplementary Table 24: Experimental ^{1}H ΔRCSA values for 35 μg of briarane B-3…………71
Supplementary Table 25: Experimental ^{1}H ΔRCSA values for 40 μg of santonin……………...72
Supplementary Table 26: Experimental ^{1}H ΔRCSA values for 45 μg of brucine……………….72
Supplementary References……………………………………………………………....83

Supplementary Note 1

Sample preparation: Preparation of deuterated PMMA gel (PMMA-d_8 gel)

Methyl-*d_3* methacrylate-*d_5* (MMA-*d_8*) and ethylene glycol dimethacrylate (EGDMA) are first purified through a basic alumina column. A 100x diluted EGDMA stock (1 v/v % EGDMA) is prepared by mixing EGDMA and acetone-*d_6* at 1:99 volume ratio. The free radical initiator, 2,2′-azobis(2-methylpropionitrile) (AIBN), is dissolved in methanol-*d_4* to 6% (w/v). For 70% (v/v) PMMA gels of 0.04 mol % cross-linking, a solution is prepared by mixing 1.4 mL MMA-*d_8*, 100 μL 1% EGDMA, 10 μL 6% AIBN, and 0.6 mL acetone-*d_6*. The mixture is purged with dry N_2 for 8 minutes, and then quickly transferred into 3 mm NMR tubes. The tubes are filled with dry N_2 and then either capped and fastened with parafilm or fire sealed. The tubes are then directly transferred into a water bath, which is heated up at 65 °C for the polymerization reaction. The reaction is generally completed after 5 hours, and tubes are allowed to cool down. Afterwards, tubes containing the deuterated PMMA gel are uncapped and left for air dry (usually 3 days). After carefully breaking the closed end of the tube, the dried gel stick is gently pulled out and cut into 2.2 cm segments. A 2-step wash is carried out to remove unreacted monomers and oligomers. First, the gel segments are washed in a 1:1 (v/v) mixture of acetone and methanol at room temperature for 1 day. Then, they are washed twice with chloroform overnight. The washed gels are then dried on a glass surface for two days. Afterwards, dry gels are ready for use or can be stored for future use. The resulting gel shows mechanical properties that allow their use in a stretching device (4.2 mm/3.2 mm). Gels compatible with Hilgenberg´s micro stretching device (MSD) (2.2/1.8 mm) were prepared in either 2 or 1.7 mm capillary tubes from Hilgenberg. PMMA-*d_8* gels can be prepared in different cross linker concentrations (i.e., from 0.009 until 0.04 mol %) showing different degrees of alignment. The crosslinker concentration of 0.009 molar % yields the best mechanical properties. The gel can be washed and reused for 3 additional samples to our best knowledge. This recipe is a modification of previously reported procedures.[1-3] In deuterated PMMA gel, only MMA-*d_8* was used. The cross linker and the radical initiator were not deuterated. The price of chemicals increases by 157 euros for the preparation of 2 ml solution of gel sample that yields around 12 gel sticks each of 2.2 cm length (calculated based on information available in https://www.sigmaaldrich.com). Thus, the price for each gel sample increases only by 13 euros, which is a marginal value comparing with the labor cost.

Supplementary Table 1. Chemicals and materials used for gel preparation of the deuterated PMMA compatible with the stretching device. OD: outer diameter; ID: inner diameter

Chemicals	Short Name	Isotopic purity (atom % D)	CAS Number / Product number
Ethylene glycol dimethacrylate	EGDMA	-	97-90-5
Methyl-d_3 methacrylate-d_5	MMA-d_8	≥ 99	35233-69-3
2,2′-Azobis(2-methylpropionitrile)	AIBN	-	78-67-1
Acetone-d_6	-	-	666-52-4
1.7 mm capillary tubes (OD=1.7/ID=1.3 mm)			2001711
2 mm capillary tubes (OD=2.0/ID=1.6 mm)			2001711

The comparison of the 1D ^{1}H NMR spectra of protonated and deuterated PMMA gels is displayed in Supplementary Figure 1.

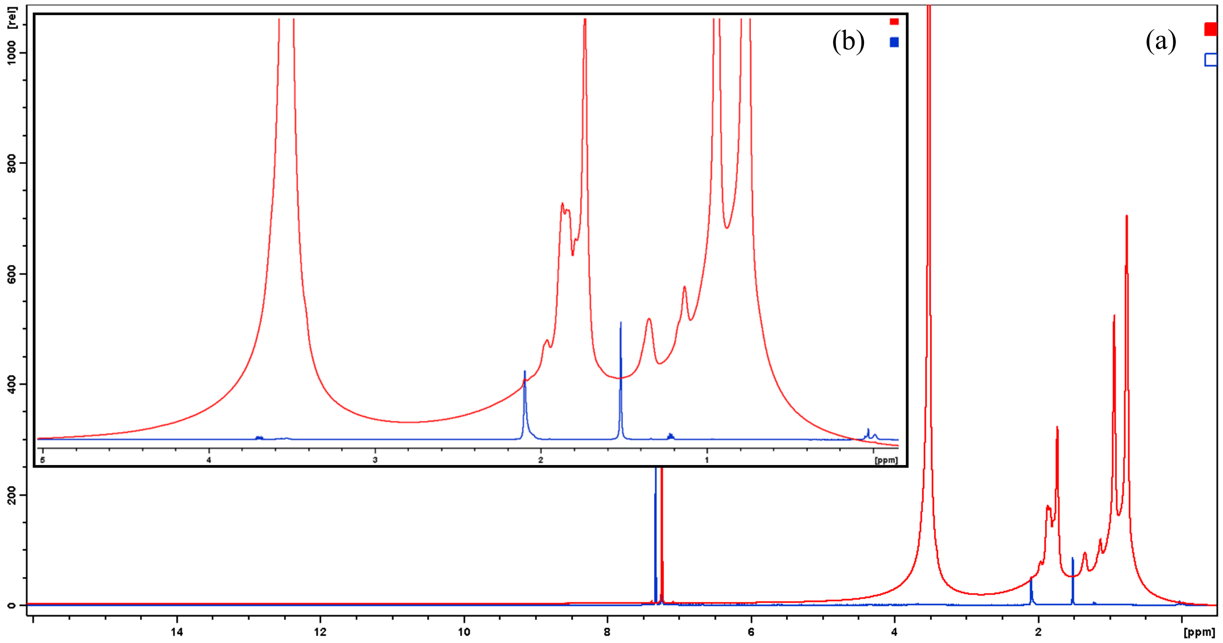

Supplementary Figure 1. ^{1}H NMR spectra of gel. Overlay of 1D ^{1}H NMR spectra of stretchable protonated PMMA gel (70/0.04, red line) and deuterated PMMA gel (70/0.05, blue line) measured in a 700 MHz Bruker spectrometer (a). The inset panel shows spectrum detail from 0 to 5 ppm (b). The former is suitable for the New Era's stretching device (4.2/3.2 mm), and the latter one is

suitable for the Hilgenberg's micro stretching device (2.2/1.8 mm). Measurements were done under minimum alignment conditions. Number of scans in all cases was 32.

Preparation of poly-HEMA gel

The polymerization conditions used in the preparation of the poly-HEMA gel were thoroughly optimized for the measurement of ^{1}H RCSA and gel containing 70% monomer and 0.04% cross-linker density was found to be optimum for the measurement. The gel was prepared by using a polymerization reaction of the monomer 2-hydroxyethylmethacrylate (HEMA) and by using ethylene glycol dimethacrylate (EGDMA), as a cross linker.[4] HEMA and EGDMA are first purified through a basic alumina column. A 100x diluted EGDMA stock (1% EGDMA) is prepared by mixing EGDMA and HEMA at 1:99 volume ratio. The free radical initiator 2,2'-Azobis(4-methoxy-2,4-dimethylvaleronitrile) (V-70) is dissolved in methanol to 6% (w/v). For 70% (v/v) poly-HEMA gels of 0.02 mol % cross-linking, a solution is prepared by mixing 1.4 mL HEMA, 50 µL 1% EGDMA, 10 µL 6% V-70, and 0.6 mL acetone. The mixture was first sonicated (10 min) and then purged with dry N_2 for 10 minutes, and then quickly transferred into 3 mm NMR tubes. The tubes are filled with dry N_2 and then either capped and fastened with parafilm or fire sealed. Polymerization is carried out by heating the reaction mixture at 55ºC for 6 hours in a water bath. After the reaction, tubes containing the gel were uncapped and kept in contact with air for two days. After two days, the dried gel stick is gently pulled out, and cut into 2 cm segments and the gel sticks are several times washed in a 1:1 (v/v) mixture of acetone and methanol and are dried again.

Liquid crystal sample preparation (5 mm and 3 mm NMR tubes)

The lyotropic liquid crystalline phases were prepared as follows. A total amount of 139-155 mg of the polymer (either PPA-L-Val$_{dec}$ or PPA-D-Val$_{dec}$)[5,6] was weighted directly into the NMR tube.

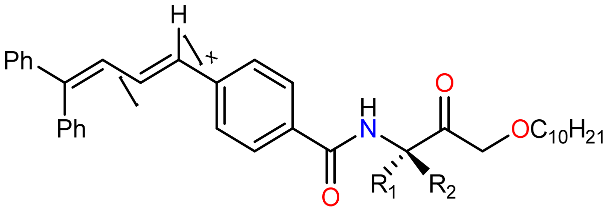

R_1 = *i*Pr; R_2=H; x = 750: *PPA-L-Val$_{dec}$*
R_1 = H; R_2= *i*Pr; x = 1000: *PPA-D-Val$_{dec}$*

A stock solution of the analyte in $CDCl_3$, was added in such a way that the polymer concentration was 18 w/v. The NMR-tube was fire sealed, and the polymer was allowed to dissolve overnight. The sample was centrifuged back and forth (1000 rpm) until the line width of the ^2H signals become sharp and constant. A most detailed description regarding sample preparation can be found as a video on the web.[7] For the liquid crystal sample preparation, 12 mg of strychnine was used in a 5 mm NMR tube, which can be reduced to 1 mg by using a 3 mm NMR tube. Down to 1 mg amount of sample, the polymer signal can be suppressed using a T_2 filtered 1D ^{1}H NMR.

Supplementary Table 2. Amount of chemicals used for the liquid crystal sample preparation

Chemical	Chemical mass (mg)	Polymer mass (mg)	$CDCl_3$ mass (mg)
Strychnine (5 mm NMR tube)	12.0	139.1	634.7
Strychnine (3 mm NMR tube)	1.0	22.4	102.0

Micro compression device (MCD) sample preparation (1.7 mm NMR tubes)

Gels for the micro compression device: The monomer and the cross-linker used for the polymerization reaction are the same as described in literature.[2] The reaction was carried out in capillary tubes that swollen gel could be inserted into 1.7 mm tube of the MCD (Supplementary Figure 2). The long PMMA gel sticks obtained from the gel reaction are cut in a way they are 4.5 cm long after swelling in $CDCl_3$. The gels prepared in this way show the same mechanical properties as the ones prepared in a 3 mm NMR tube; washing, pre-swelling, and dialysis was done as before to prepare the gel for measurement. For measurement, gel compression was done by a gel swollen in chloroform (40 µL) inside a 1.7 mm tube of the MCD which operates in the same way as the 5 mm one.[8] ^{1}H-RCSAs can be measured at two different compressions. Nevertheless, maximum and minimum alignment conditions were used through maximally and slightly compressed gel for the measurements in such a way that they provided the biggest possible RCSAs. For measurement, the device can be directly used in any cryo probe with appropriate deuterated solvent for locking and shimming. Supplementary Figure 3 shows a ^{1}H NMR spectrum of strychnine (70 µg) in a non-deuterated PMMA (80/0.24) gel using a 1.7 mm micro-compression device (Supplementary Figure 2).

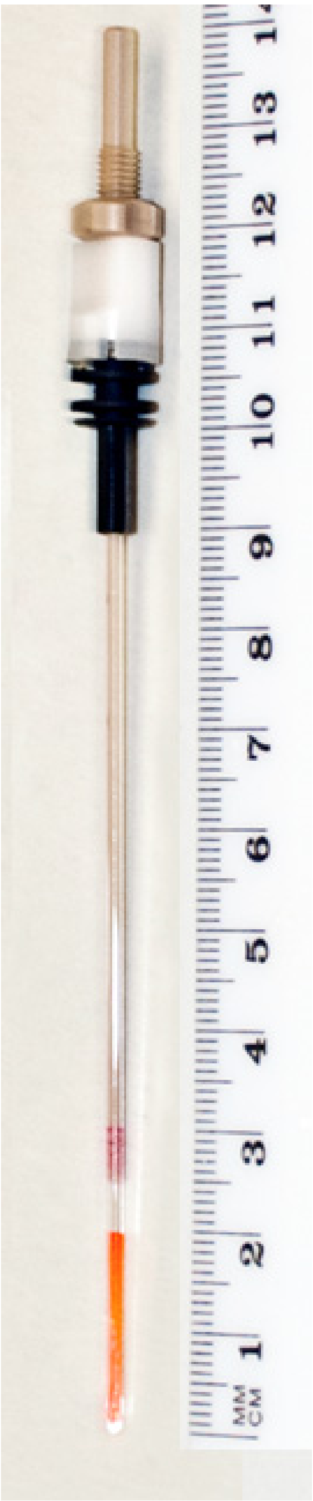

Supplementary Figure 2. Gel micro compression device. 1.7 mm MCD with a PMMA gel (in orange) swollen in $CDCl_3$. A natural pigment was used to show the colourless gel.

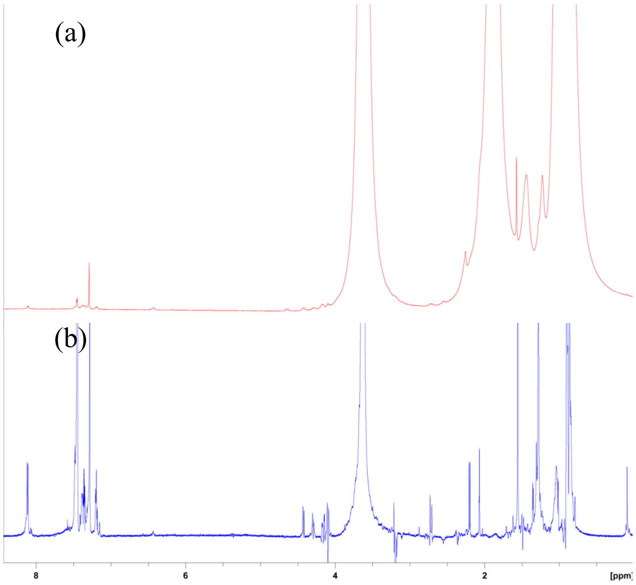

Supplementary Figure 3. ^{1}H NMR spectrum of strychnine in gel. 1D ^{1}H NMR spectra of 70 μg strychnine sample in 1.7 mm MCD in a protonated PMMA gel in which no polymer signal suppression was applied (a). Signal suppression by using a T_2 filtered was also applied (b).

Supplementary Note 2

Micro Stretching Device (MSD)

The main goal of developing the micro stretching device (MSD) is to measure RDCs and RCSAs at microgram scale in combination with the deuterated PMMA gels, introduced for this work. The MSD was conceived at the Max Planck Institute for Biophysical Chemistry (Göttingen) and manufactured by the German glass company Hilgenberg. The device, as the one described by Liu *et al.*,[9] is a two side open NMR tube, with an outside diameter of 3 mm (Supplementary Figures 4a and 4b). The tube provides two sections with different diameters to induce diverse alignment conditions necessary for RCSAs measurement. The gel stick is initially swollen in a 5 mL Eppendorf. It is then delivered into the larger diameter section of 2.2 mm, by using a Teflon device, which reminds us of a syringe (Supplementary Figure 4c) At this point, the gel may not get stretched or slightly stretched providing a minimum alignment condition at which the first spectrum is collected. Afterwards, the swollen gel is moved through the device by a glass plunger and pushed into the narrower section of the tube, producing the maximum alignment condition (Supplementary Figure 4d).

The device is now available with 3 different narrowed section diameters being compatible with both 5 mm and 3 mm cryoprobes (2.2/1.8; 1.6 and 1.4 mm). The Hilgenberg provides this device either with Teflon®, silicon or rubber stoppers, which are compatible with $CHCl_3$, MeOH and DMSO. So far, our research group has developed a recipe for PMMA-d_8 and adapted the currently existing one for PMMA, poly-HEMA and poly-DEGMEMA[10] gels for these devices; meaning that samples compatible with $CHCl_3$, DMSO and MeOH can be analized (Supplementary Table 3). By using the MSD along with PMMA-d_8, it was possible to accurately measure ^{1}H RCSAs and subsequently determine the relative configuration of (-)-α-santonin, brucine, strychnine and finally briarane B-3, a new diterpene isolated from *Briareum asbestinum*.

The gel stick is inserted inside the widest section of the MSD (2.2 mm) by using a reduced and modified version of the New Era's device. The main difference is that the whole device is made of Teflon, and neither metallic parts nor *O*-rings are used. During the insertion of the gel into the device, there is an internal funnel that guides the gel and slightly passes from the wider diameter tube part chamber to the 2.2 mm tube section. Generally, the gel is tight enough to be held by the device at this minimal stretching condition; in some cases, it gives measurable deuterium splitting in the wide section. Sometimes, few drops of deuterated solvent are added from the other end of the tube to help the delivery and stretching process. Quadrupolar splittings ($\Delta\nu_Q$) observed under stretching conditions, i.e. 1.8, 1.6 and 1.4 mm inner diameter are reported in Supplementary Table 3.

Supplementary Table 3. Gel composition and its quadrupolar splitting under different stretching conditions afforded by several MSDs.

Gel composition	Inner diameter (mm)	$\Delta\nu_Q$ (Hz)
PMMA[a] (70/0.05)	1.8	32.0
PMMA-*d8*[a] (70/0.05)	1.8	17.0
PMMA-*d8*[b] (70/0.02)	1.8	54.0
Poly-HEMA[c] (60/0.04)	1.8	3.6
PMMA-*d8*[c] (70/0.009)	1.8	22.6
Poly-DEGMEMA[b] (70/0.2)	1.6	4.8
Poly-DEGMEMA[b] (70/0.2)	1.4	7.1

[a]$\Delta\nu_Q$ was measured in a Bruker spectrometer running at 800 MHz for ^{1}H, [b] measured in a spectrometer running at 900 MHz and [c] measured in a spectrometer running at 600 MHz.

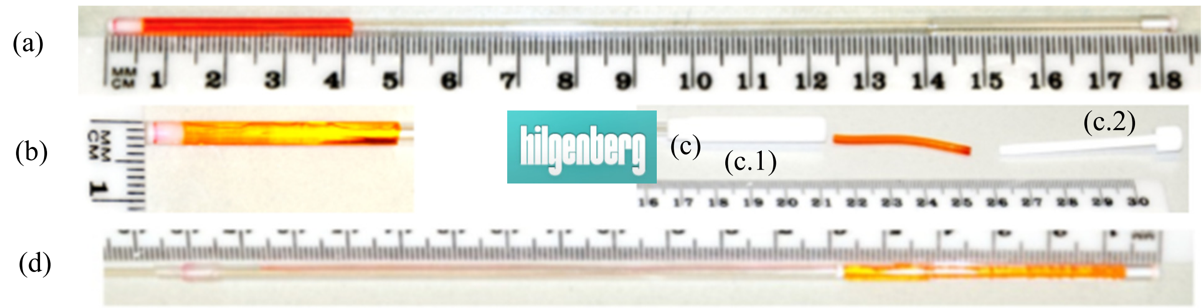

Supplementary Figure 4. Hilgenberg's micro-stretching device. The devices have three different inner diameters (1.4, 1.6 and 1.8 mm); a highly resistant 3 mm borosilicate glass NMR tube provided with two open sides and two inner diameters (a and b). Gel swelling in a sample solution (orange color in the figure) usually takes 24 h. Then the swollen gel stick (4.5 cm long) is pushed inside the MSD using a dedicated delivery device comprising a chamber (c.1) and piston (c.2). Once the gel is inside the 2.2 mm section of the tube, isotropic data can be recorded. Afterwards, the gel is stretched by pushing it from the wide region of the device (2.2 mm) into the narrow section (1.8 mm) with a glass piston (2 mm diameter). The piston is kept in its final position by wrapping with Teflon® tape (d). The gel shown here is a PMMA (70/0.05) swollen in $CDCl_3$ with a natural pigment.

Supplementary Note 3

Computational methods: Strychnine

Strychnine has six chiral centers whose different permutations leads to 64 different configurations. Excluding enantiomers only 32 configurations are left. The configurations were labelled via the *R* or *S* configuration of carbons C7, C8, C13, C12, C14, and C16 respectively, for example *RSSRRS* for the correct configuration (See Supplementary Figure 5). For strychnine, the chemical shift tensors required for analysis for thirteen different B3LYP/6-31G(d) optimized geometries were computed using the Gauge-Independent Atomic Orbital (GIAO) method[11] using Density Functional Theory (DFT) at B3LYP/6-311+G(2d,p) level. Solvation was taken into account using the Polarizable Continuum Model using the integral equation formalism variant (IEFPCM)[12] method with $CHCl_3$ parameters in Gaussian09.[13] Since the multicyclic constitution is not

compatible with all configurations, only 13 diastereomers are sterically possible (Supplementary Figure 5).

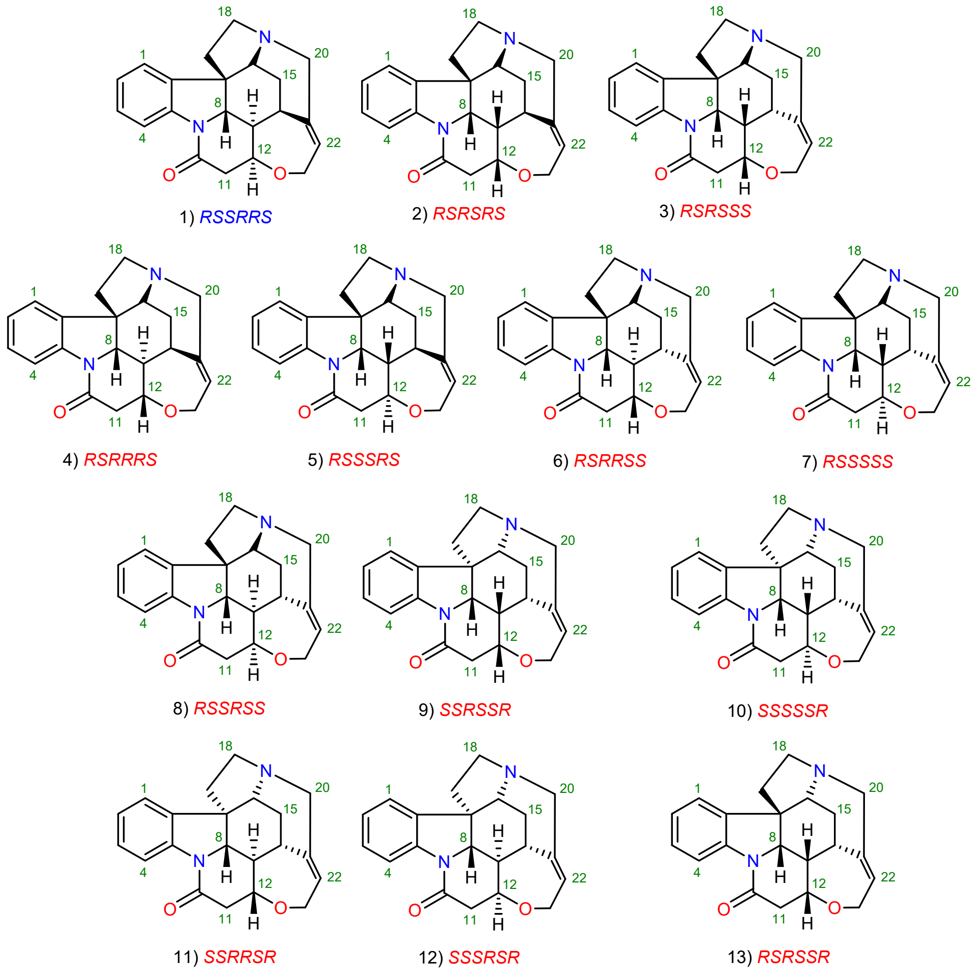

Supplementary Figure 5. Configurations of strychnine. The 13 sterically feasible diastereomers of strychnine are shown, whose carbons C7, C8, C13, C12, C14, and C16 are labelled either the *R* or *S* descriptors. The designation *RSSRRS* denotes the correct configuration. Chemical shift tensors were calculated at GIAO/B3LYP/6-311+G(2d,p)//B3LYP/6-31G(d) level using the IEFPCM solvent method with $CHCl_3$ parameters. The correct configuration, 1 *RSSRRS*, is coloured in blue while the incorrect ones (2-12) were coloured in red.

Estrone

The conformational search of estrone and 13-*epi*-estrone was based on molecular mechanics calculations using Discovery Studio 2.5 (Accelrys) and the standard Consistent Force Field (CFF)

for organic molecules. The "generate conformations" protocol with the FAST search method was used in the software. Within a 33 kJ mol^{-1} energy threshold, two conformers of estrone and four conformers of 13-*epi*-estrone were generated. These conformers were further optimized at DFT level B3LYP/6-311(d) using the IEFPCM solvent model with DMSO parameters. From the calculation, it is found that only the lowest energy conformer is populated for both estrone and 13-*epi*-estrone. The chemical shift tensors were calculated at DFT level B3LYP/6-311+G(2d,p) using the same solvent model.

Retrorsine

Since retrorsine is a flexible molecule, we observed ensemble averaged NMR data. Therefore, for the configuration analysis of retrorsine, the complete structural ensembles that are compatible with the NMR data must be considered. We have used the same set of conformers for different configurations that were used for ^{13}C RCSAs.[3] All the conformers present within an energy threshold of 9 kJ mol^{-1} were generated using force field Merck molecular force field (MMFF94)[14] in Macromodel software. The chemical shift tensors were calculated at DFT level GIAO/B3LYP/6-31+G(2d,p).

Supplementary Table 4. Number of conformers for the 8 different configurations of retrorsine.

Configuration	Number of conformers
RRRR	8
RRRS	4
RRSR	3
RRSS	9
RSRR	3
RSRS	8
RSSR	7
RSSS	10

Briarane B-3

The planar structure of briarane B-3 was created in ChemDraw Professional V17.0. Conformational analysis of briarane B-3, using an energy cut off of 2.5 $kcalmol^{-1}$, was performed with Discovery studio using the MMFFs force field in vacuum. Conformers were chosen for the

gas phase geometrical optimization calculation using DFT methods with the B3LYP functional and the 6–31+G(d,p) basis-set as implemented with the Gaussian 09 program package. Vibrational analysis was performed at the same level to confirm stability of the minima. For the electronic circular dichroism (ECD) calculation, time-dependent density function theory (TDDFT) calculations were performed at CAM-B3LYP/6-311++G(2d,p) and PBE0/Def2TZVP levels using the IEFPCM method with acetonitrile parameters (24 excited states of briarane B-3 were used during the ECD calculation). ECD curves were calculated based on rotatory strengths using half bandwidth of 0.3 eV using SpecDis version 1.70.1.[15] The ECD spectra were constructed based on the Boltzmann-weighting according to their population contribution.

Supplementary Note 4

Chemical shift tensor calculation

We evaluate the robustness of ^{1}H RCSA calculations by two different DFT methods over all the configurations of strychnine with the data from the 80 µg sample ($CDCl_3$) in PMMA-d_8 (70/0.04) recorded at an 800 MHz NMR spectrometer by using a stretching device (4.2/3.2 mm).
It is expected that the proton chemical shift tensor is highly sensitive to the solvent model and also to the functional/basis set chosen. Therefore, NMR shielding tensors were computed with two DFT methods. We use equivalent levels of theory and compare the 'Continuous Set of Gauge Transformations' (CSGT) method[16] with the aforementioned GIAO method for the computation of NMR shifts. Generally, both CSGT and GIAO methods were used with very popular and well tested functionals for ^{1}H chemical shift calculations, namely with basis sets, viz., B3LYP/6-311+G(2d,p)[16,17] and PBE0/cc-pVTZ.[12,18,19] The latter combination is found to be suitable especially for ^{1}H-chemical shift calculations.[20,21] Solvent model dependence was estimated by using IEFPCM method[12] in the first step and the Conductor-like Screening Model (COSMO)[12] in the last step; both solvent models have been successfully employed in assignment of small molecules from chemical shifts.[22] In our own experience, the strychnine system seems to be slightly better described with B3LYP, which has better performance (R^2=0.9984), but in general terms both combinations are appropriate as both show a R^2 bigger than 0.995.[23] Results are shown in Supplementary Table 4 and Supplementary Figure 6. Once both methods have been shown to correlate well with experimental data, we proceed to obtain both quality factors; Q and Q_{CSA}.

Supplementary Table 5: Proton chemical shift of strychnine (δ_H) in ppm and DFT computed chemical shift values (σ_{calc}).

Proton number	DFT Numbering [a]	$\delta_{H\,exp}$ (ppm)	σ_{calc} (GIAO/B3LYP) (ppm)[b]	σ_{calc} (CSGT/PBE0) (ppm)[b]
**4**	29	8.055	23.381	23.223
**3**	28	7.228	24.341	23.907
**1**	26	7.162	24.349	23.847
**2**	27	7.075	24.551	24.013
**22**	37	5.958	25.678	25.056
**13**	34	1.265	30.789	30.165
**15a**	41	1.473	30.473	29.920
**17b**	44	1.904	30.046	29.384
**17a**	45	1.904	29.975	29.384
**15b**	42	2.352	29.508	28.957
**12**	33	4.264	27.541	26.991
**8**	30	3.851	27.887	27.416
**16**	43	4.033	27.765	27.311
**20b**	39	2.802	29.222	28.614
**23b**	36	4.133	27.624	27.206
**23a**	35	4.039	27.526	27.105
**11b**	32	2.638	29.155	28.788
**11a**	31	3.104	28.782	28.378
**14**	40	3.152	28.654	28.119
**18b**	47	2.896	28.874	28.378
**18a**	46	3.310	28.674	28.228
**20a**	38	3.752	27.921	27.552

[a] Numbering of the different ^1H nuclei of strychnine in DFT files.[b] The calculated chemical shifts were not referenced. Experiments were measured in an 800 MHz Bruker spectrometer in $CDCl_3$.

a)

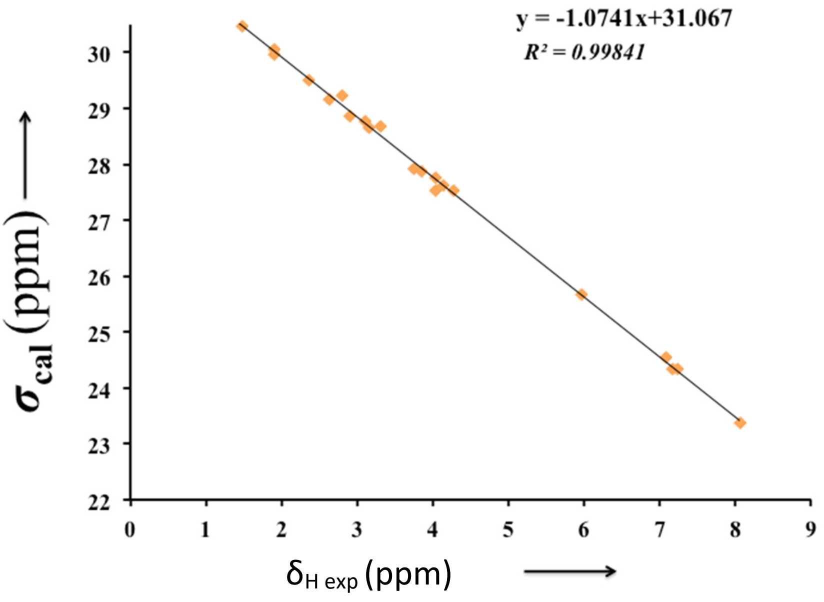

b)

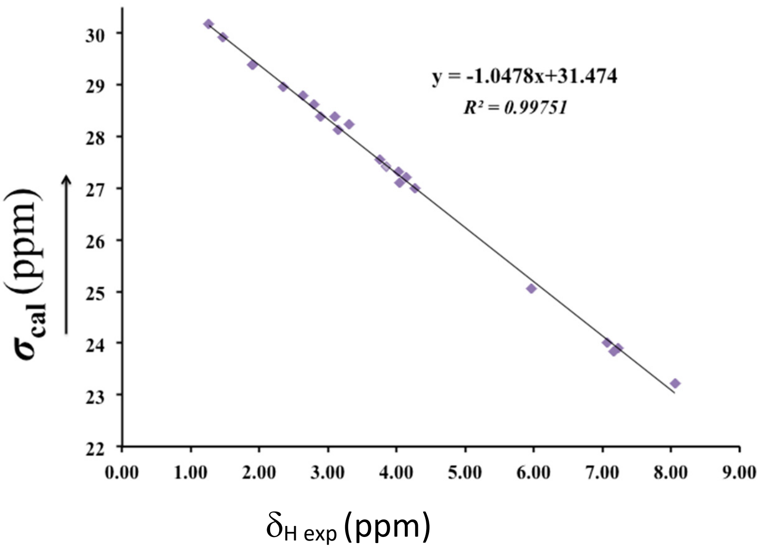

Supplementary Figure 6. Chemical shift correlation. Experimental ^{1}H chemical shifts ($\delta_{H\ exp}$) of strychnine (800 MHz; $CDCl_3$) and the isotropic chemical shifts (σ_{calc}, not referenced) computed at: a) GIAO/B3LYP/6-311+G(2d,p)/IEFPCM (Orange-top) level and b) CSGT/PBE0/cc-pVTZ/COSMO (Purple-bottom) level.

Supplementary Note 5

Calculation of standard deviation associated with ^{1}H RCSAs

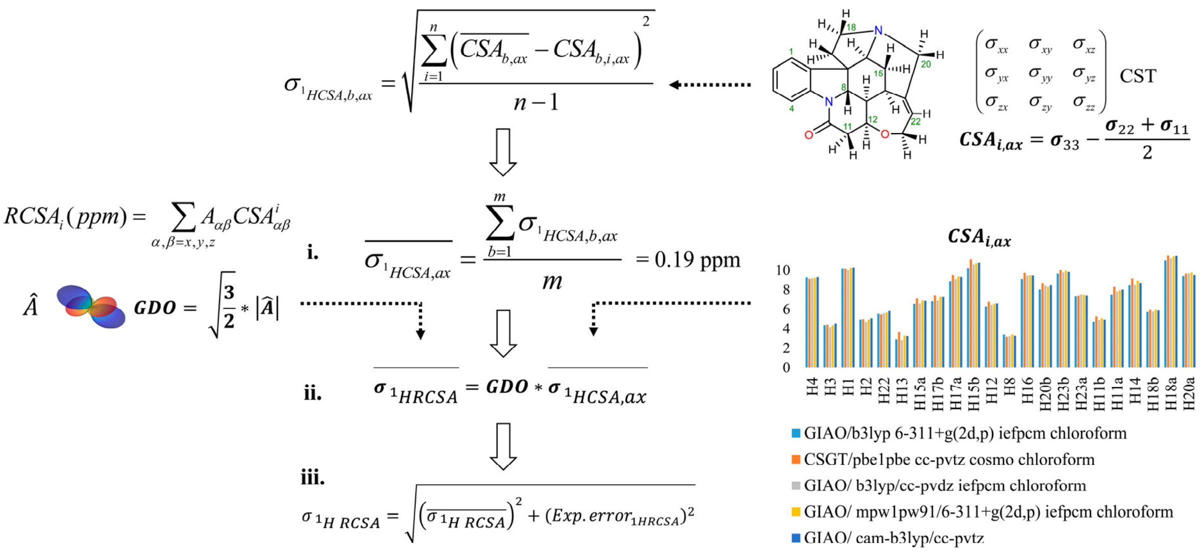

Supplementary Figure 7. Error calculation. Workflow for calculating the standard deviation associated with ^{1}H RCSA ($\sigma_{^1H\,RCSA}$) in three simple steps i.e., (i), (ii) and (iii).

Standard deviation associated with back-calculated and experimental ^{1}H RCSAs was computed using a 3 step workflow, described in Supplementary Figure 7, as follows: The chemical shift anisotropy (CSA) for the correct configuration of strychnine was computed at n = five levels of theory (Gaussian09, running index i). Then, the standard deviation of the axial component of the CSA for each proton (*b)* was calculated ($\sigma_{1HCSA,b,ax}$) by using Supplementary Equation (1) Afterwards, the average uncertainty associated with the back-calculated ^{1}H CSAs ($\overline{\sigma_{1HCSA,ax}}$) was estimated by using Supplementary Equation (2)

$$\sigma_{^1HCSA,b,ax} = \sqrt{\frac{\sum_{i=1}^{n}\left(\overline{CSA_{b,ax}} - CSA_{b,i,ax}\right)^2}{n-1}} \qquad \text{Supplementary Equation (1)}$$

$$\overline{\sigma_{^1HCSA,ax}} = \frac{\sum_{b=1}^{m}\sigma_{^1HCSA,b,ax}}{m} \qquad \text{Supplementary Equation (2)}$$

where; n: is the number of different computational approaches taken, meaning functional/basis set combination (5). $CSA_{b,i,ax}$: axial CSA for each proton and DFT approach. $\overline{CSA_{b,ax}}$: average of $CSA_{b,i,ax}$. m: is the number of ^{1}H nuclei in strychnine for which RCSAs were determined (22 in total, see Supplementary Table 8)

Variation of ^{1}H $CSA_{b,i,ax}$ (b is the running index over the protons presents in strychnine) when different levels of theory (i) are used is depicted in Supplementary Figure 8 and Supplementary Table 6.

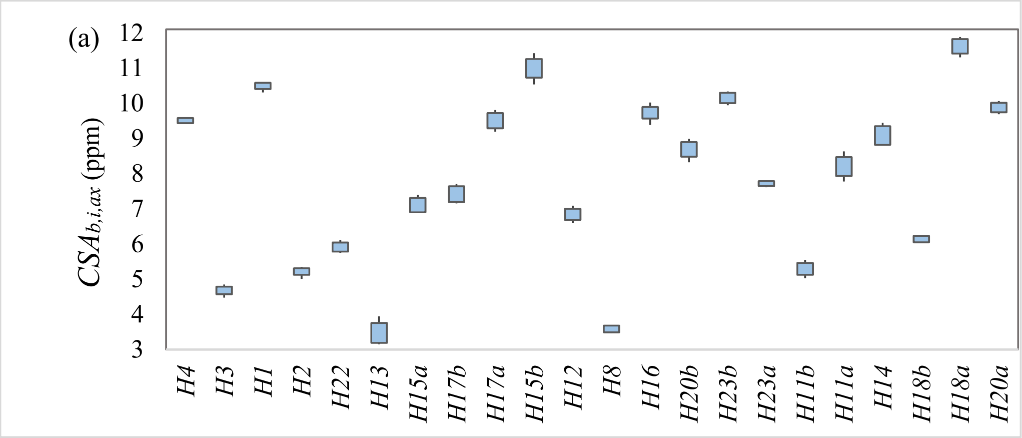

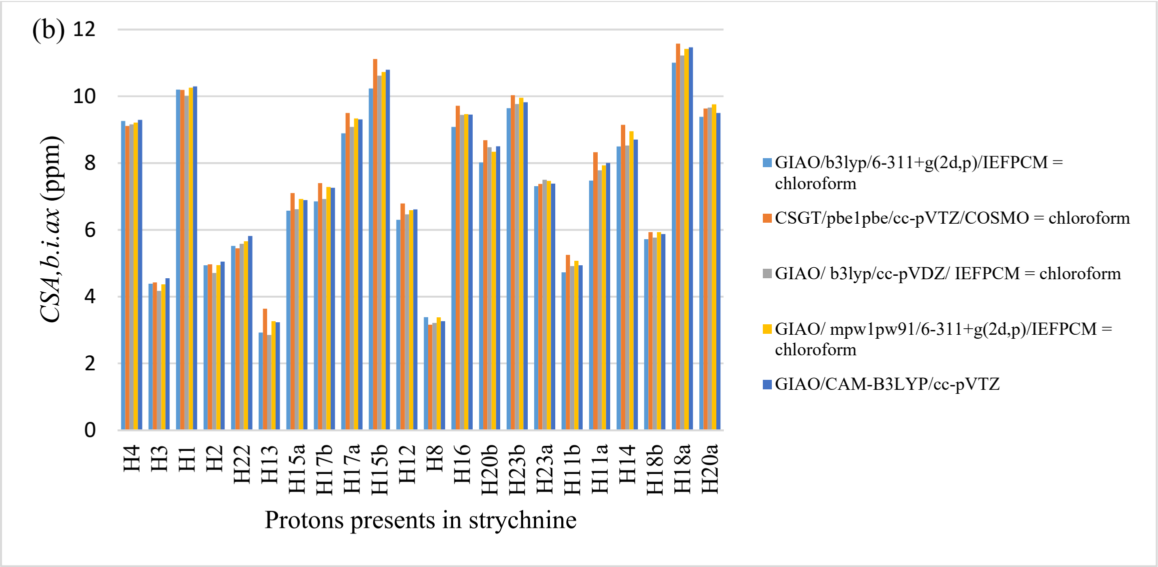

Supplementary Figure 8. Variation of proton CSA. Variation of *RSSRRS*-strychnine ^{1}H $CSA_{b,i,ax}$ is depicted by the box and whisker plot in (a) with $\sigma_{^{1}HCSA,b,ax}$ depicted as size of the boxes. b) ^{1}H $CSA_{b,i,ax}$ represented as bars for each of the 5 calculations depending on the different DFT levels (n).

Supplementary Table 6. Axial part of ^{1}H CSA for the different protons of *RSSRRS* configuration of strychnine. IEFPCM and COSMO are two different solvent models.[12] $\sigma_{CSA,b,ax}$ is the standard deviation of the axial component of the CSA from the 5 different models for each proton b. The averages over all protons ($^{1}H_{b}$) is reported in the lowest row.

Proton	DFT level 1	DFT level 2	DFT level 3	DFT level 4	DFT level 5	$\overline{\sigma CSA_{b,ax}}$	$\sigma\ _{^{1}HCSA,b,ax}$
**H4**	9.26	9.11	9.16	9.22	9.30	9.21	0.08
**H3**	4.38	4.43	4.17	4.37	4.55	4.38	0.14
**H1**	10.20	10.20	10.01	10.26	10.30	10.19	0.11
**H2**	4.94	4.97	4.71	4.95	5.05	4.92	0.13
**H22**	5.52	5.45	5.58	5.66	5.82	5.61	0.14
**H13**	2.93	3.64	2.85	3.27	3.23	3.18	0.31
**H15a**	6.57	7.10	6.62	6.92	6.89	6.82	0.22
**H17b**	6.85	7.40	6.93	7.28	7.26	7.14	0.24
**H17a**	8.89	9.50	9.08	9.34	9.31	9.23	0.24
**H15b**	10.23	11.12	10.62	10.73	10.80	10.70	0.32
**H12**	6.30	6.79	6.46	6.59	6.61	6.55	0.18
**H8**	3.38	3.16	3.21	3.38	3.27	3.28	0.10
**H16**	9.08	9.72	9.44	9.47	9.45	9.43	0.23
**H20b**	8.02	8.69	8.47	8.34	8.50	8.40	0.25
**H23b**	9.64	10.03	9.77	9.95	9.82	9.84	0.15
**H23a**	7.31	7.37	7.50	7.47	7.39	7.41	0.08
**H11b**	4.73	5.25	4.92	5.07	4.94	4.98	0.19
**H11a**	7.48	8.33	7.78	7.93	8.00	7.91	0.31
**H14**	8.50	9.14	8.53	8.95	8.70	8.77	0.28
**H18b**	5.72	5.93	5.77	5.94	5.87	5.85	0.10
**H18a**	11.00	11.58	11.22	11.42	11.47	11.34	0.23
**H20a**	9.39	9.63	9.66	9.76	9.50	9.59	0.15
**Avg.**	7.29	7.66	7.38	7.56	7.55	7.49	**0.19**

DFT level from 1 to 5 are: GIAO/b3lyp/6-311+G(2d,p)/IEFPCM(chloroform), CSGT/PBE1PBE/cc-pVTZ/COSMO (chloroform), GIAO/B3LYP/cc-PVDZ/IEFPCM (chloroform), GIAO/MPW1PW91/6-311+G(2d,p)/ IEFPCM (chloroform), GIAO/CAM-B3LYP/cc-pVTZ. Average: Avg.

Finally, the standard deviation based on the variation of functional/basis set associated with the computed CSA ($\overline{\sigma\ {}^{1}{}_{HCSA,ax}}$) averaged over all protons b was found to be 0.19 ppm [(Supplementary Equation (2)]. We took this standard deviation for all further error discussions.

Now, we introduce the uncertainty of ^{1}H RCSA ($\overline{\sigma\ {}^{1}{}_{HRCSA}}$) associated with any molecule [step (ii)]:

$$\overline{\sigma\ {}^{1}{}_{HRCSA}} = GDO^{*}\ \overline{\sigma\ {}^{1}{}_{HCSA,ax}} \qquad \text{Supplementary Equation (3)}$$

where, *GDO* is the generalized degree of order of the alignment tensor computed without taken any error source into account (See Supplementary Figure 7, step 2 and Supplementary Table 11). The GDO of an alignment tensor $\hat{A}$ is defined as:

$$\text{GDO}=\sqrt{\frac{3}{2}}\ |\hat{A}|$$

Finally, the standard deviation associated with the ^{1}H RCSA ($\sigma\ {}^{1}{}_{H\ RCSA}$) is composed of the error of the computed RCSA ($\overline{\sigma\ {}^{1}{}_{H\ RCSA,}}$) and the experimental error of measurement ($Exp.error_{1H\ RCSA}$) by using the following mathematical expression [See Supplementary Figure 7, step (iii)]:

$$\sigma\ {}^{1}{}_{H\ RCSA} = \sqrt{\left(\overline{\sigma\ {}^{1}{}_{HRCSA}}\right)^{2} + (Exp.error_{1H\ RCSA})^{2}} \qquad \text{Supplementary Equataion (4)}$$

$Exp.error_{1H\ RCSA}$, the experimental error associated with the proton residual chemical shift anisotropy is estimated as described in the literature.[4] For $Exp.error_{1H\ RCSA}$ we average also over all protons b. The error $\sigma\ {}^{1}{}_{H\ RCSA}$ is used later for the calculation of the standard deviation of Q and Q_{CSA}.

Supplementary Note 6

Comparison of Q and Q_{CSA} factors computed at different levels of theory

Both quality factors were calculated through procedures well described in the literature[3] using NMR shielding tensors computed by using the above-discussed different approaches: GIAO and CSGT and for the solvent models IEFPCM and COSMO. Irrespective of the mode of calculations, the results for Q and Q_{CSA} are very similar.

Supplementary Table 7. Bar plot showing the *Q* factors for thirteen configurations of strychnine (80 µg sample) aligned in a stretching device (4.2/3.2 mm) and PMMA-d_8 (70/0.04). *Q* factors of strychnine computed at DFT levels GIAO/B3LYP/6-311+G(2d,p)/IEFPCM and CSGT/PBE0/cc-pVTZ/COSMO are given in the column chart. Error bars are expressed as the standard deviation of *Q*, computed through Monte Carlo analysis.

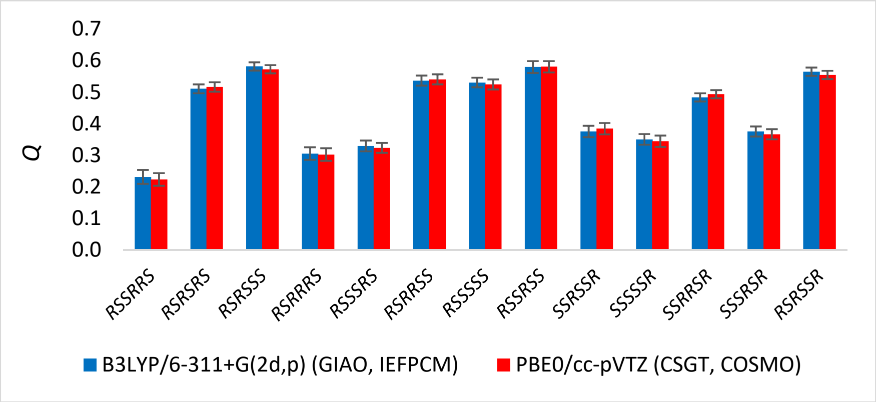

Configuration		Q_{B3LYP}±σ*		Q_{PBE0}±σ**	
1	*RSSRRS*	0.208	0.015	0.213	0.015
2	*RSRSRS*	0.479	0.014	0.483	0.014
3	*RSRSSS*	0.597	0.013	0.592	0.013
4	*RSRRRS*	0.271	0.015	0.274	0.015
5	*RSSSRS*	0.352	0.015	0.35	0.015
6	*RSRRSS*	0.496	0.014	0.503	0.014
7	*RSSSSS*	0.542	0.013	0.534	0.014
8	*RSSRSS*	0.553	0.013	0.567	0.013
9	*SSRSSR*	0.359	0.015	0.356	0.015
10	*SSSSSR*	0.333	0.015	0.32	0.015
11	*SSRRSR*	0.527	0.013	0.532	0.013
12	*SSSRSR*	0.411	0.015	0.401	0.015
13	*RSRSSR*	0.622	0.012	0.62	0.013

*CSA computed at GIAO/B3LYP/6-311+G(2d,p)/ IEFPCM. **CSA computed at CSGT/PBE0/cc-PVTZ /COSMO. Solvent parameters used in the calculations were for chloroform.

Supplementary Table 8. Q_{CSA} factors of strychnine (80 µg sample) at DFT levels GIAO/B3LYP/6-311+G(2d,p)/ IEFPCM and CSGT/PBE0/cc-pVTZ/COSMO. Error bars are expressed as the standard deviation of Q_{CSA}, computed through Monte Carlo analysis.

**Configuration**		**$Q_{CSA/B3LYP}$±σ***		**$Q_{CSA/PBE0}$±σ****	
**1**	*RSSRRS*	0.231	0.022	0.223	0.020
**2**	*RSRSRS*	0.510	0.014	0.516	0.015
**3**	*RSRSSS*	0.581	0.013	0.572	0.013
**4**	*RSRRRS*	0.305	0.02	0.302	0.020
**5**	*RSSSRS*	0.329	0.017	0.323	0.016
**6**	*RSRRSS*	0.536	0.016	0.540	0.016
**7**	*RSSSSS*	0.530	0.015	0.524	0.016
**8**	*RSSRSS*	0.579	0.019	0.580	0.018
**9**	*SSRSSR*	0.375	0.018	0.384	0.018
**10**	*SSSSSR*	0.350	0.017	0.344	0.018
**11**	*SSRRSR*	0.483	0.013	0.493	0.013
**12**	*SSSRSR*	0.375	0.016	0.366	0.016
**13**	*RSRSSR*	0.564	0.013	0.554	0.013

*CSA computed at GIAO/B3LYP/6-311+G(2d,p)/IEFPCM. **CSA computed at CSGT/PBE0/cc-PVTZ/COSMO. Solvent parameters used in the calculations were for chloroform.

Supplementary Table 9. Bar plot showing the Q factors (blue bar) and Q_{CSA} factors (red bar) are reported for thirteen configurations of strychnine (10 µg sample) aligned in PMMA-d_8 (70/0.05). Data was recorded in a 700 MHz NMR spectrometer by using a MSD (2.2/1.8 mm). CSA was computed at DFT level GIAO/B3LYP/6-311+G(2d,p)/IEFPCM, with choloform solvent parameters. Error bars are expressed as the standard deviation of Q(Q_{CSA}), computed through Monte Carlo analysis.

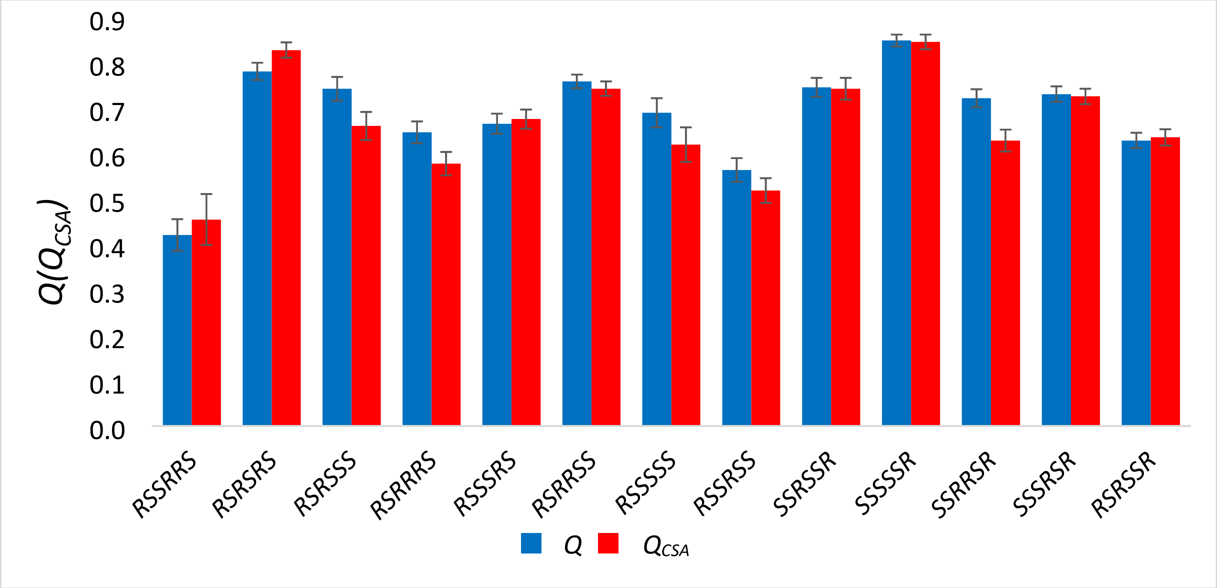

Configuration	σ 1H RCSA. (Hz)[a]	*Q* ± σ		*Q* Max	*Q* Min	*Q*CSA ± σ		*Q*CSA Max	*Q*CSA Min
RSSRRS	0.35	0.425	0.035	0.310	0.516	0.455	0.056	0.651	0.296
RSRSRS	0.28	0.781	0.019	0.837	0.719	0.828	0.017	0.875	0.789
RSRSSS	0.35	0.743	0.026	0.801	0.651	0.661	0.031	0.772	0.564
RSRRRS	0.28	0.648	0.024	0.723	0.577	0.578	0.026	0.65	0.492
RSSSRS	0.28	0.667	0.022	0.735	0.577	0.676	0.021	0.74	0.608
RSRRSS	0.21	0.759	0.015	0.799	0.710	0.743	0.016	0.788	0.700
RSSSSS	0.42	0.689	0.032	0.782	0.579	0.62	0.038	0.758	0.510
RSSRSS	0.28	0.564	0.026	0.631	0.475	0.519	0.027	0.598	0.429
SSRSSR	0.28	0.746	0.021	0.803	0.685	0.743	0.024	0.797	0.680
SSSSSR	0.21	0.849	0.013	0.885	0.810	0.846	0.016	0.887	0.801
SSRRSR	0.28	0.720	0.020	0.770	0.671	0.629	0.024	0.691	0.565
SSSRSR	0.21	0.731	0.017	0.785	0.683	0.726	0.017	0.771	0.686
RSRSSR	0.21	0.628	0.017	0.670	0.577	0.636	0.018	0.697	0.580

[a] ^{1}H RCSA standard deviation (σ 1H RCSA) was computed by using Supplementary Equation (4).

Supplementary Note 7

Relative configuration analysis of strychnine in liquid crystal

Measurement of ^{1}H RCSAs of strychnine in liquid crystal uses different temperatures to induce different alignment conditions necessary for RCSA measurement, schematically shown in Supplementary Figure 9a. 12 mg of strychnine is aligned in the liquid crystal in a 5 mm NMR tube. The sample showed maximum alignment at 300 K and minimum alignment at 315 K. The RCSAs were computed by using Supplementary Equation (3) provided in the manuscript and RCSAs have values in between -12.0 and 10.4 Hz, measured in a Bruker spectrometer running at 800 MHz. In this case, both ^{13}C and ^{1}H RCSA analysis furnished slightly higher Q factors for the correct configuration (0.122±0.006 and 0.343 ± 0.022) in relation to those data data collected in PMMA gel. The Q(Q_{CSA}) factors are 0.343(0.439) ± 0.022(0.030) for the correct *RSSRRS* and 0.413(0.419) ± 0.021(0.023) for the next closest incorrect *RSSSRS* configuration. The results obtained for different configurations of strychnine from liquid crystal ^{1}H RCSA data is represented in the bar diagram in Supplementary Figure 9b.

(a)

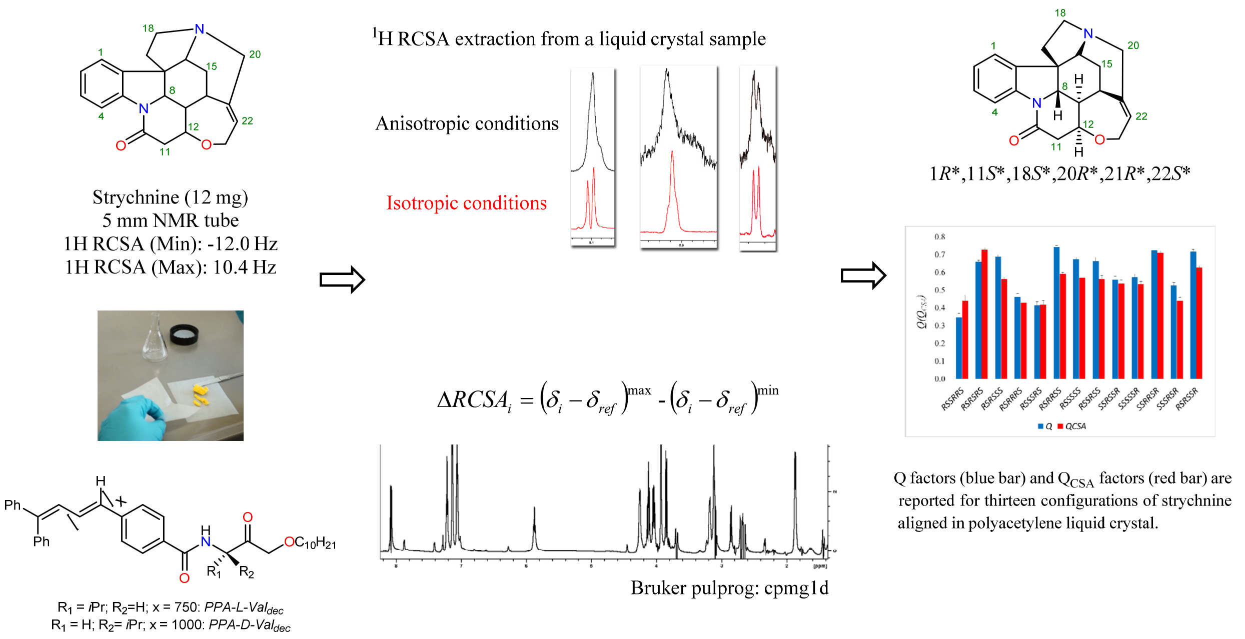

(b)

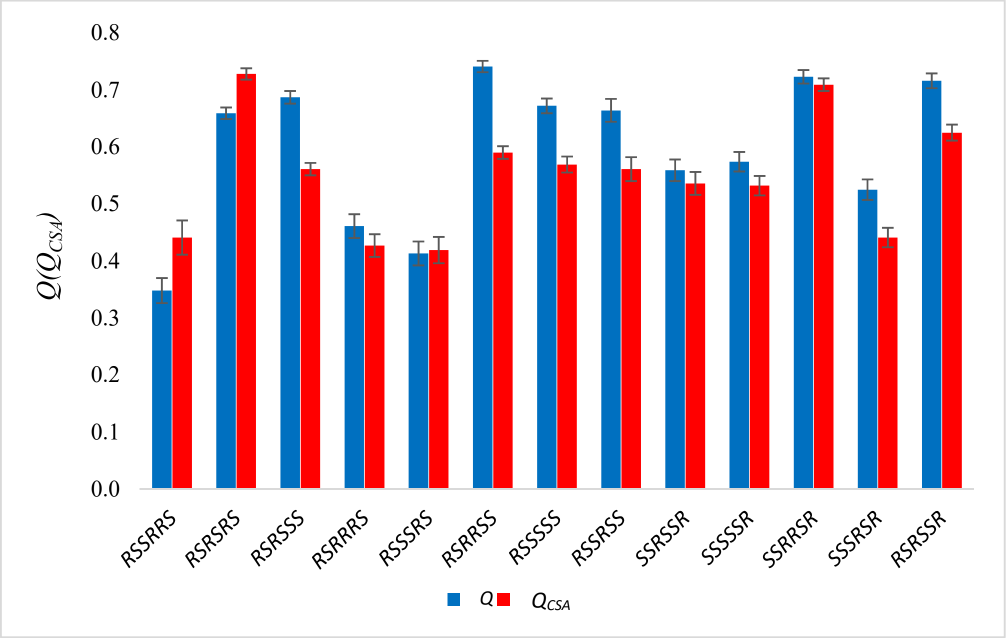

Configuration		$Q \pm \sigma$		$Q_{CSA} \pm \sigma$	
**1**	*RSSRRS*	0.348	0.022	0.441	0.030
**2**	*RSRSRS*	0.659	0.010	0.728	0.010
**3**	*RSRSSS*	0.687	0.011	0.561	0.011
**4**	*RSRRRS*	0.461	0.021	0.427	0.020
**5**	*RSSSRS*	0.413	0.021	0.419	0.023
**6**	*RSRRSS*	0.741	0.010	0.590	0.011
**7**	*RSSSSS*	0.672	0.013	0.569	0.014
**8**	*RSSRSS*	0.664	0.020	0.561	0.021
**9**	*SSRSSR*	0.559	0.019	0.536	0.020
**10**	*SSSSSR*	0.574	0.017	0.532	0.017
**11**	*SSRRSR*	0.723	0.012	0.709	0.011
**12**	*SSSRSR*	0.525	0.018	0.441	0.017
**13**	*RSRSSR*	0.716	0.013	0.625	0.014

*CSA computed at GIAO/B3LYP/6-311+G(2d,p)/IEFPCM. Solvent parameters for chloroform were used in the calculations. Error bars are expressed as the standard deviation of $Q(Q_{CSA})$, computed through Monte Carlo analysis.

Supplementary Figure 9. Configuration analysis. Workflow for measuring ^{1}H RCSA using as alignment media liquid crystal (a). The Q factors (blue bar) and Q_{CSA} factors (red bar), including its $Q(Q_{CSA})$ standard deviations, are reported for thirteen configurations of strychnine aligned in polyacetylene liquid crystal (b).

Supplementary Note 8

Relative configuration analysis of estrone in poly-HEMA gel

3 mg of estrone are aligned in a poly-HEMA gel swollen in DMSO-d_6 by using a stretching device (4.2/3.0 mm). The measured RCSAs range from -1.52 to -7.36 Hz at a ^{1}H spectrometer frequency of 800 MHz. The analysis was done for the two configurations, estrone and 13-*epi*-estrone. While the analysis of ^{13}C RCSA data collected in DMSO compatible gel did not assign correctly the configurations[24] ^{1}H-RCSAs did assign correctly: The SVD-fitted ^{1}H RCSA data analysis provided lowest Q(Q_{CSA}) factors of 0.212(0.188)±0.017(0.022) while for 13-*epi*-estrone, they are 0.445(0.482)±0.022(0.026), respectively. The difference of 0.233 and 0.294 for Q and Q_{CSA} factors together with the respective standard deviations shows that configuration differentiation is possible using ^{1}H RCSA only. It is worth mentioning that ^{13}C RCSA data acquired in PMMA gel furnished Q factors of 0.09 and 0.13 for estrone and 13-*epi*-estrone, respectively. In order to find DFT benchmarking in determining the effect of solvents in the computation of the anisotropy of CSA tensors and also in finding robust functionals and basis sets, CSA tensors were calculated using different DFT functionals, such as B3LYP and MPW1PW91, and second-order Møller–Plesset perturbation theory calculations (MP2). Here we looked at different solvents as opposed to the situation in strychnine where only $CDCl_3$ was used. The Q factors obtained for estrone and 13-*epi*-estrone are tabulated (Supplementary Table 10). We find that the results are consistent in different theoretical models. It is noteworthy that proton CSA values for different models follow the identical trends (Supplementary Table 10 and Supplementary Figure 10b with the exception of computation with smaller basis sets. The $Q(Q_{CSA})$ factors (Supplementary Figure 10b) computed from CST at DFT level GIAO/B3LYP/6-311+g(2d,p) and using the IEFPCM solvent method.

Supplementary Table 10. Q factor calculated for estrone and 13-*epi*-estrone in different theoretical models/different solvents.

Functional/basis set	Solvent	Q(estrone)	Q (13-*epi*-estrone)
MPW1PW91/6-311+g(2d,p)	DMSO	0.214	0.443
B3LYP/6-311+g(2d,p)	DMSO	0.206	0.443
B3LYP/6-311+g(2d,p)	$CHCl_3$	0.205	0.443
MPW1PW91/6-31+g(2d,p)	DMSO	0.193	0.439
B3LYP/6-311+g(2d,p)	MeOH	0.219	0.443
MPW1PW91/3-21g	DMSO	0.210	0.450
MP2/6-311g+(2d,p)	$CHCl_3$	0.185	0.456

(a)

(b)

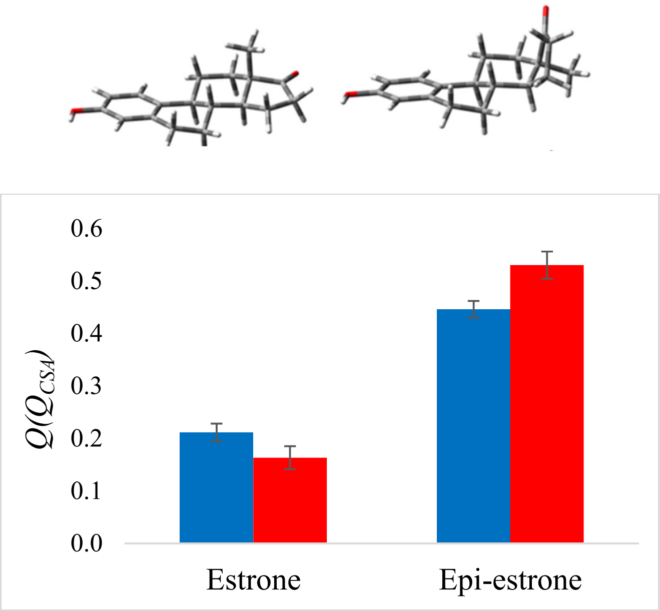

Supplementary Figure 10. Configuration analysis of estrone. a) 3D structures of estrone and epi-estrone. b) The Q factors (blue bar) and Q_{CSA} factors (red bar) for estrone and 13-*epi*-estrone. The error bars are standard deviations.

Supplementary Table 11. Alignment tensor´s GDO, standard deviation ($\sigma_{1H\ RCSA}$), Q and Q_{CSA} factor associated with the ^1H RCSA analysis of each retrorsine´s configuration. The difference in the $\sigma_{1H\ RCSA}$ for the different configuration originates from different GDOs. ^1H RCSA were measured in an 800 MHz Bruker spectrometer.

Configuration	GDO	$\sigma\ {}^1H_{RCSA}$ (Hz)	$Q \pm \sigma$	$Q_{CSA} \pm \sigma$
RRRS-Retrorsine	1.05E-03	0.160	0.273 ± 0.018	0.343 ± 0.019
RRRR-Retrorsine	1.05E-03	0.160	0.380 ± 0.021	0.251 ± 0.020
RRSR-Retrorsine	6.88E-04	0.080	0.606 ± 0.008	0.594 ± 0.011
RRSS-Retrorsine	1.05E-03	0.160	0.340 ± 0.019	0.297 ± 0.020
RSRR-Retrorsine	9.88E-04	0.160	0.584 ± 0.017	0.624 ± 0.018
RSRS-Retrorsine	8.82E-04	0.160	0.627 ± 0.016	0.571 ± 0.021
RSSR-Retrorsine	8.30E-04	0.160	0.596 ± 0.016	0.631 ± 0.021
RSSS-Retrorsine	3.33E-03	0.480	0.399 ± 0.046	0.695 ± 0.056

Supplementary Table 12. Alignment tensor's GDO, standard deviation [$\boldsymbol{\sigma}$ $_{^1H\ RCSA}$ derived from Supplementary Equation (4)], Q and Q_{CSA} factor associated with the ^{1}H RCSA analysis of estrone. ^{1}H RCSA were measured in an 800 MHz Bruker spectrometer.

Configuration	GDO	σ $_{^1H\ RCSA}$ (Hz)	$Q \pm \sigma$	$Q_{CSA} \pm \sigma$
Estrone	1.458E-03	0.273	0.211 ± 0.017	0.163 ± 0.022
13-*epi*-estrone	1.377E-03	0.263	0.446 ± 0.016	0.530 ± 0.026

Supplementary Table 13. Alignment tensor´s GDO, standard deviation [σ $_{^1H\ RCSA}$ derived from Supplementary Equation (4)], Q and Q_{CSA} factor associated with the ^{1}H RCSA analysis of each possible briarene B-3's configuration. ^{1}H RCSA were measured in an 800 MHz Bruker spectrometer.

Configuration	GDO	σ $_{^1H\ RCSA}$ (Hz)	$Q \pm \sigma$	$Q_{CSA} \pm \sigma$
6*S*,7*R*,8*R*,9*S*,17*R*,1*S*,2*S*,10*S*,11*R*-briarane B-3	1.24E-03	0.189	0.284 ± 0.027	0.308 ± 0.031
6*S*,7*R*,8*R*,9*S*,17*R*,1*S*,2*R*,10*S*,11*R*-briarane B-3	1.13E-03	0.172	0.315 ± 0.032	0.376 ± 0.042
6*S*,7*R*,8*R*,9*S*,17*R*,1*R*,2*S*,10*R*,11*S*-briarane B-3	7.33E-04	0.111	0.139 ± 0.026	0.170 ± 0.034
6*S*,7*R*,8*R*,9*S*,17*R*,1*R*,2*R*,10*R*,11*S*-briarane B-3	7.81E-04	0.119	0.315 ± 0.033	0.375 ± 0.042

Supplementary Note 9

NMR/HR-MS data and analysis for briarane B-3

Briarane B-3 was detected as its sodium adduct ([M+Na$^+$]) with an ion peak at *m/z* 505.1584 (calc. *m/z* value for $C_{24}H_{31}O_8NaCl^+$ is 505.1600). For the ^{1}H and ^{13}C chemical shift assignment, 1D ^{1}H, COSY, HSQC were recorded in $CDCl_3$ for 2 mg of the sample in an 800 MHz spectrometer. To demonstrate that one could work with few 10 micrograms, comparatively less sensitive HMBC was recorded in 800 MHz spectrometer equipped with a cryo probe that took 2 and half days of measurement time. To determine the configuration of several chiral centers, the NOESY

experiment was performed. The ^{1}H and ^{13}C chemical shift assignments are provided in Supplementary Table 14 and spectra are provided in Supplementary Figures 11-14.

In order to demonstrate that constitution of briarane B-3 can also be determined from micromolar concentration, a full set of the NMR experiments was recorded with microgram quantitites from a 35 μg sample; ^{1}H, COSY, HSQC, HMBC, *J*-HMBC, HSQC-HECADE and NOESY. Number of scans is indicated with the acronym NS. In those experiments where non-uniform sampling was used p%/N^{t}/N will denote the number of complex points being p% out of N^{t} points leading to N acquired complex points. The isotropic NMR spectra for 35 μg sample are provided in Supplementary Figures 16-22.

Supplementary Table 14. ^{1}H and ^{13}C chemical shifts assignment of briarane B-3.

Carbon numbering	δ_C (ppm)	δ_H (ppm)	Multiplicity and *J* (Hz)
1	44.9		
2	79.3	4.81	d (9.4)
3	28.5	2.41, 1.87	2.41 dt (15.6, 9.4, 9.4)-1.87 dd (15.6, 10.9)
4	28.5	2.61, 1.62	2.61 dd (15.0,10.9)-1.62 m
5	142.6		
6	67.3	4.83	bs
7	78.2	5.77	bs
8	82.9		
9	74.4	5.11	d (5.1)
10	38.5	2.99	dd (4.7, 5.1)
11	46.8	2.61	dq (4.7,7.4)
12	202.7		
13	126.1	5.97	d (10.4)
14	154.7	6.19	d (10.4)
15	19.0	1.16	s
16	119.9	5.69, 5.37	bs-s
17	42.3	2.41	q (7.7)
18	10.1	1.30	d (7.7)
19	176.4		
20	15.1	1.30	d (7.4)
21Ac	170.3		
22Ac	169.6		
$CH_3$21	21.9	2.14	s
$CH_3$22	21.2	2.24	s
OH		3.43	bs

dd: doublet of doublets; dt: doublet of triplets; dq: doublet of quartet; bs: broad singlet; d: doublet; s: singlet; t: triplet; q: quartet; m: multiplet.

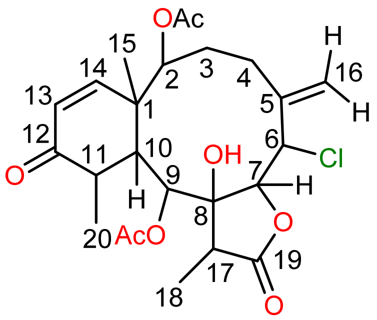

(a)

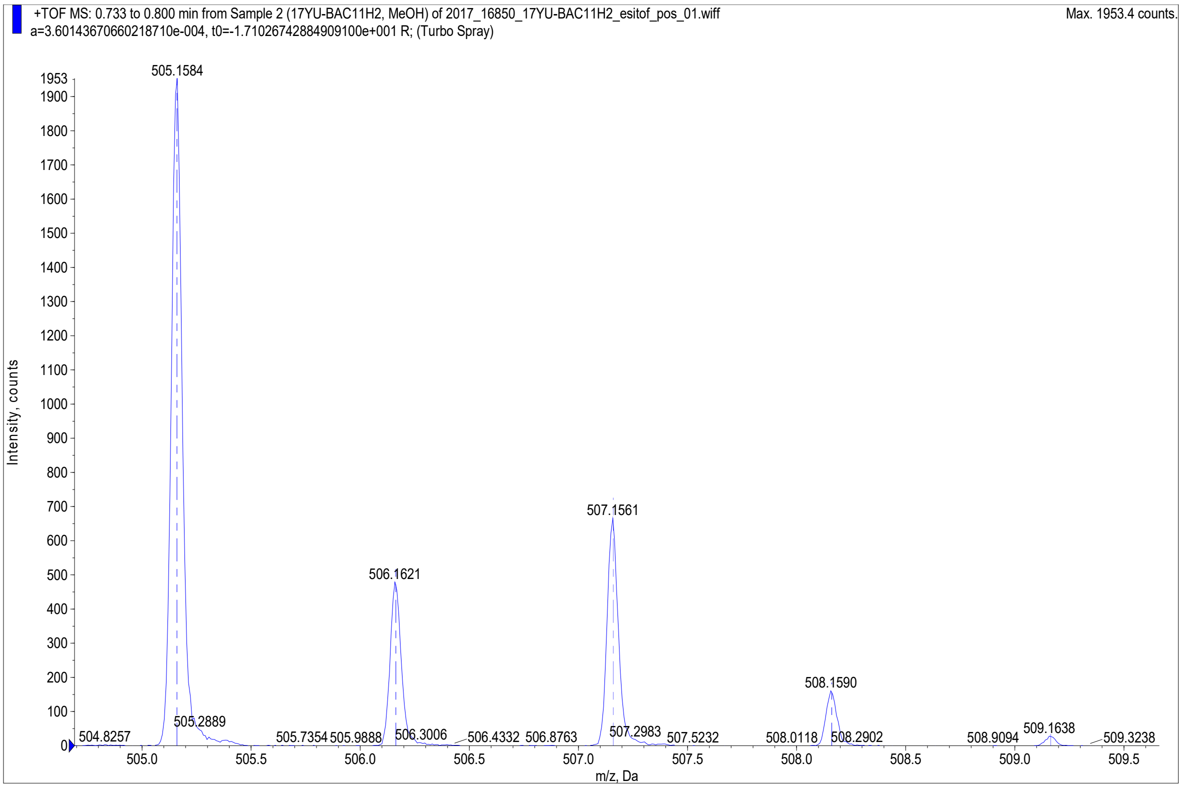

+TOF MS: 0.733 to 0.800 min from Sample 2 (17YU-BAC11H2, MeOH) of 2017_16850_17YU-BAC11H2_esitof_pos_01.wiff
a=3.60143670660218710e-004, t0=-1.71026742884909100e+001 R; (Turbo Spray)
Max. 1953.4 counts.
Intensity, counts
m/z, Da
505.1584
506.1621
507.1561
508.1590
509.1638

(b)

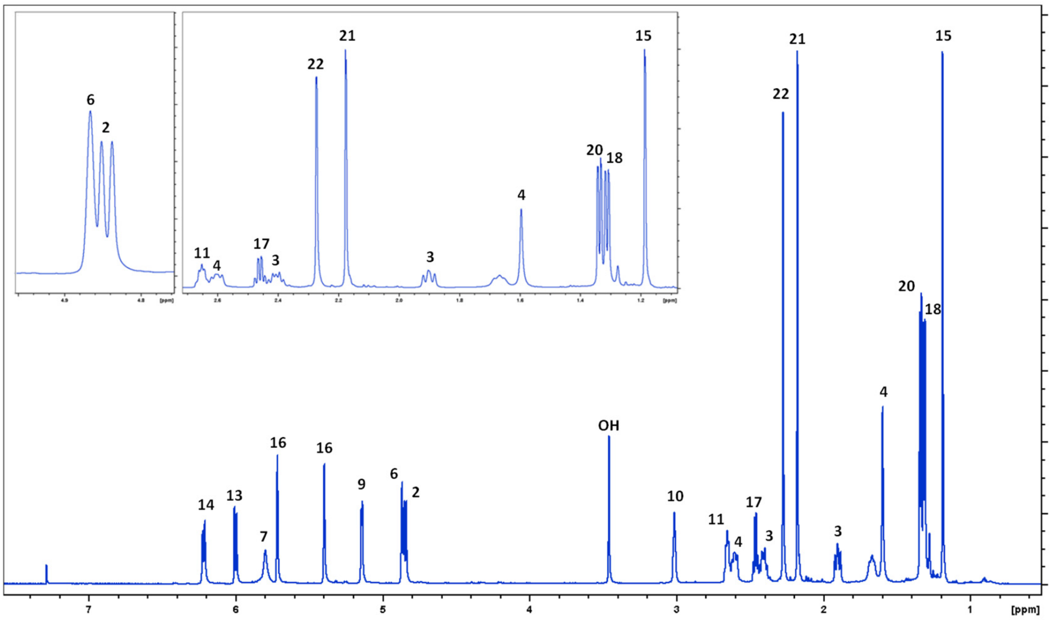

Supplementary Figure 11. **Regular HR-MS and proton NMR.** a) HR-MS spectrum (positive-ion mode) of briarane B-3. b) 1D ^{1}H spectrum of briarane B-3 (2 mg), whose structure along with atom numbering is shown on the top. Measured in a Bruker spectrometer running at 800 MHz. NS: 8.

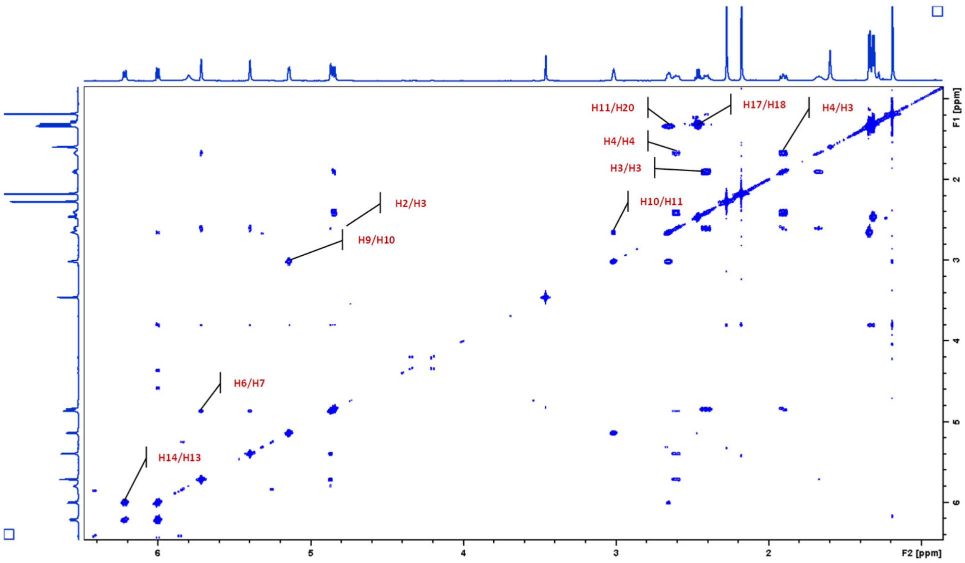

Supplementary Figure 12. Proton-proton correlation. 2D COSY spectrum of briarane B-3 (2 mg). Measured in a Bruker spectrometer running at 700 MHz. (*cosyqf45*; NS: 16)

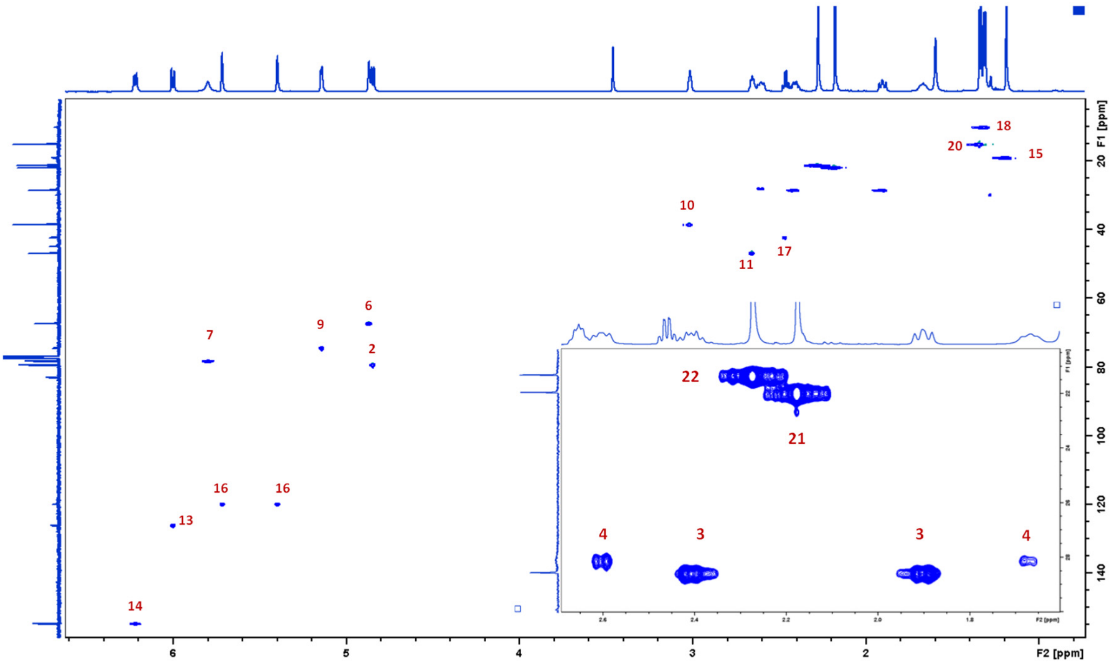

Supplementary Figure 13. Proton-carbon correlation. 2D HSQC pure shift spectrum of briarane B-3 (2 mg). Measured in a Bruker spectrometer running at 700 MHz. (NS: 16)

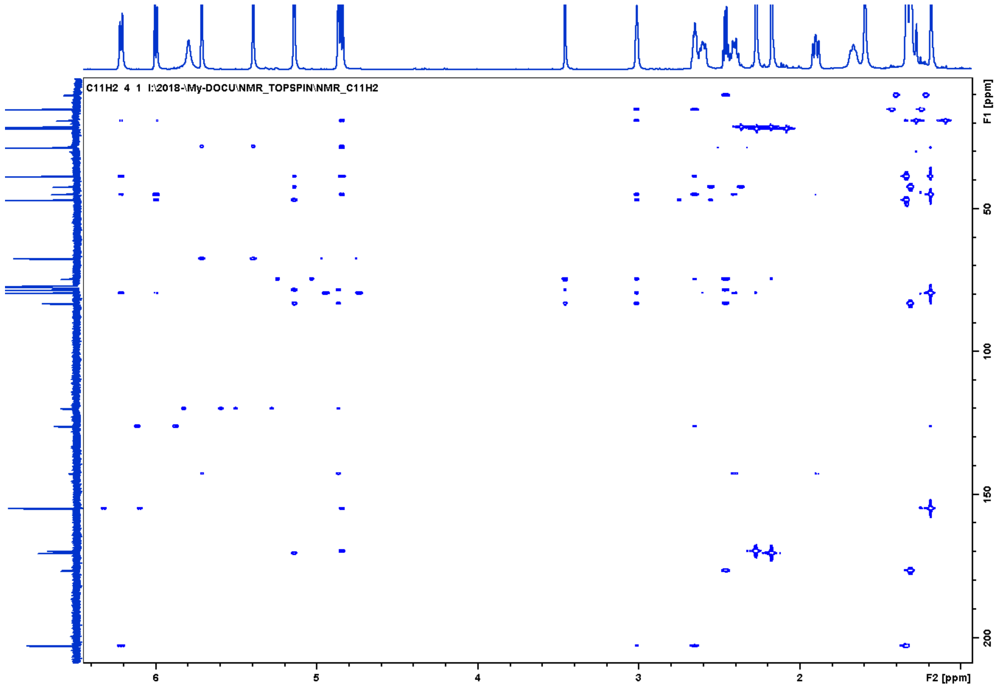

Supplementary Figure 14. Long range proton carbon correlation. HMBC spectra for briarane B-3 (35 µg). Measured in a Bruker spectrometer running at 700 MHz. (*hmbcetgpnd*; NS 40)

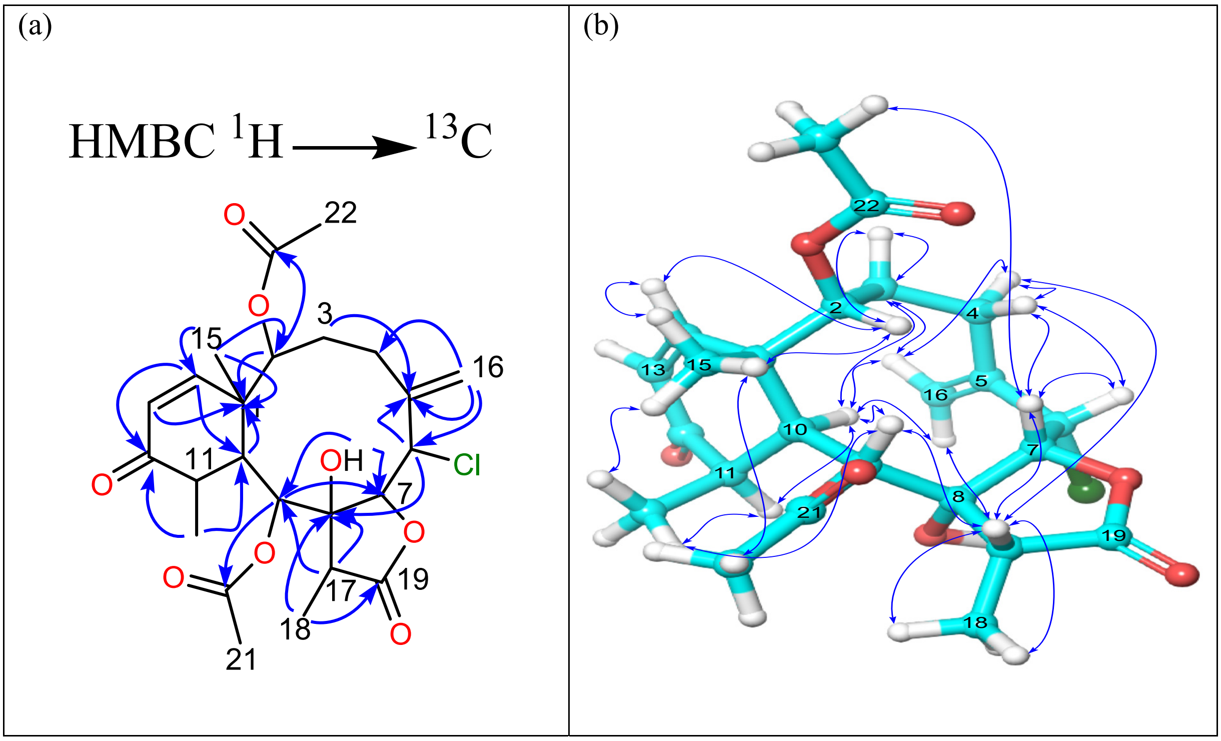

Supplementary Figure 15. Key *J* coupling and distance correlations. a) Key HMBC correlations. b) Key NOESY correlations, derived from a 2 mg sample of briarane B-3.

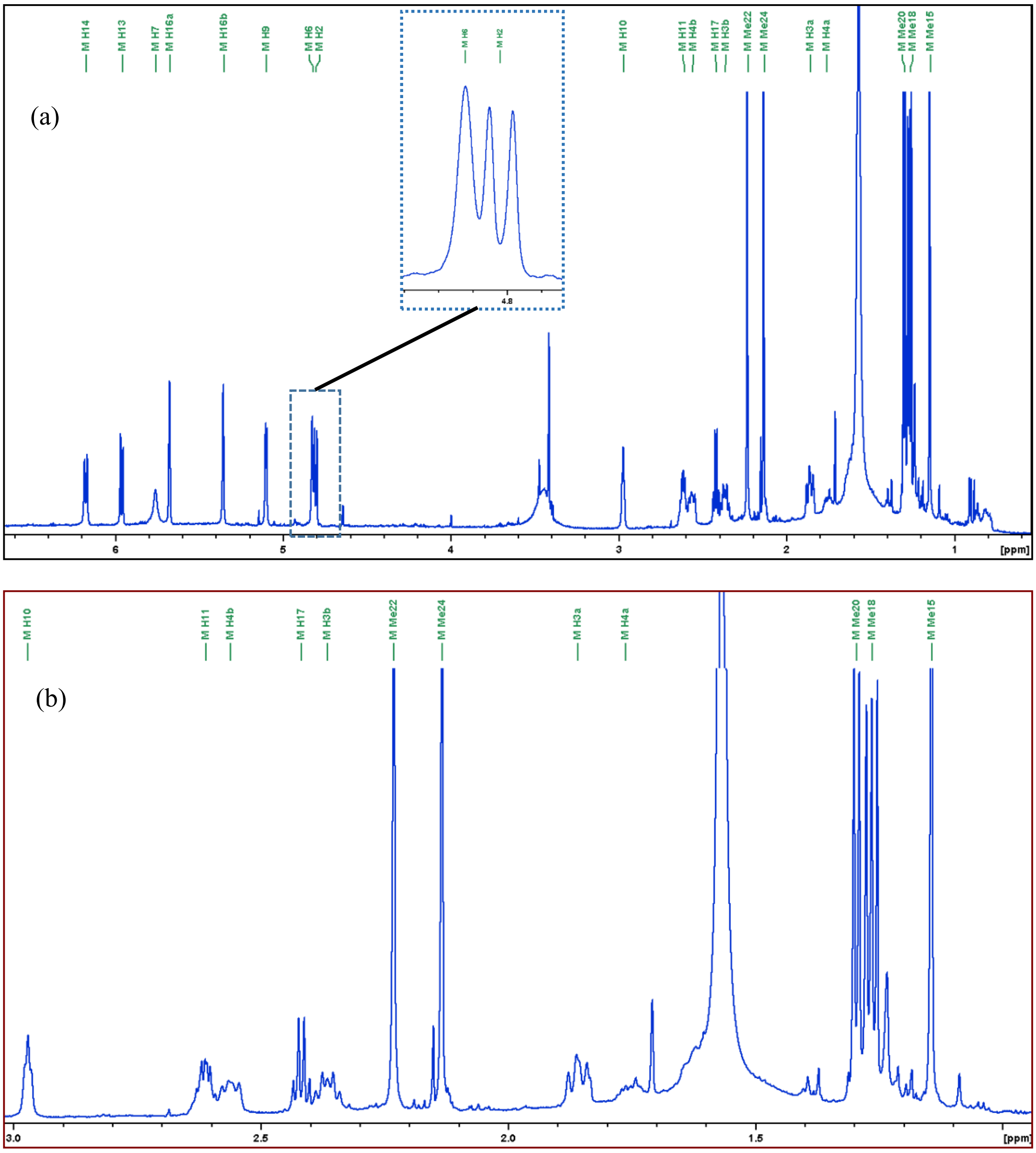

Supplementary Figure 16. Regular proton NMR of 35 µg sample. a) 1D ^{1}H NMR spectra of briarane B-3 (35 µg) using a 1.7 mm NMR tube measured in a 700 MHz spectrometer. The spectrum has sufficient signal-to-noise ratio allowing the full assignment of all the proton present in the briarane B-3. b) Expanded region of the spectrum (from 1 to 3 ppm) is shown in the lower part of the figure confirming the full assignment. (*zggppr*; NS: 400)

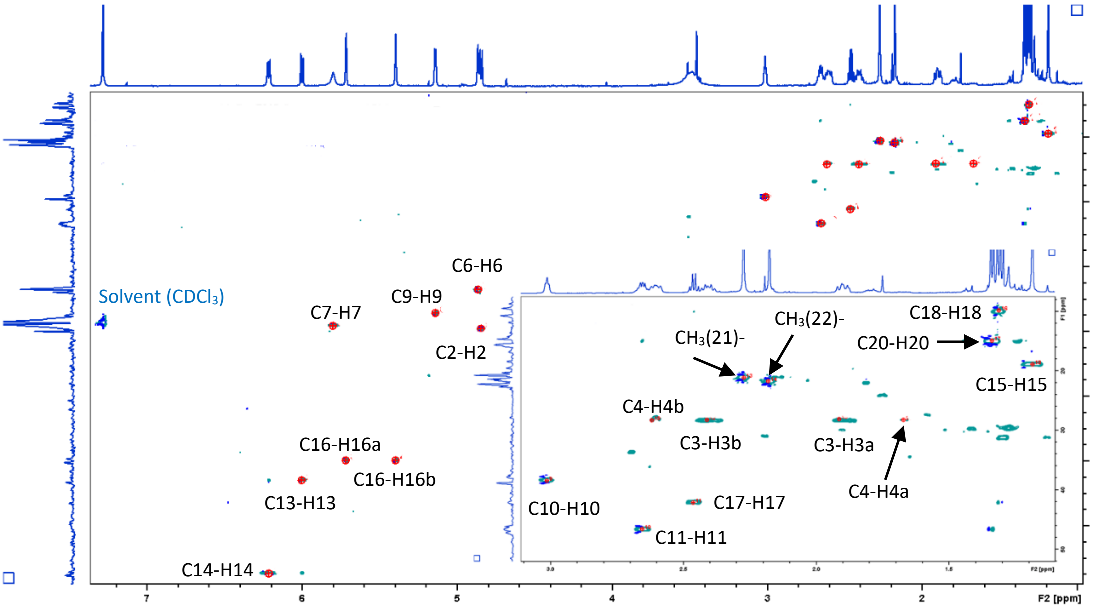

Supplementary Figure 17. Proton-carbon correlation of 35 µg sample. HSQC spectrum of briarane B-3 (35 µg) using a 1.7 mm NMR tube measured in a 700 MHz spectrometer. (*hsqcetgpsisp2.2*). $^1J_{CH}$ used was 140 Hz. Relaxation delay 2.0 s. NS: 160. Experimental time: 36 h.

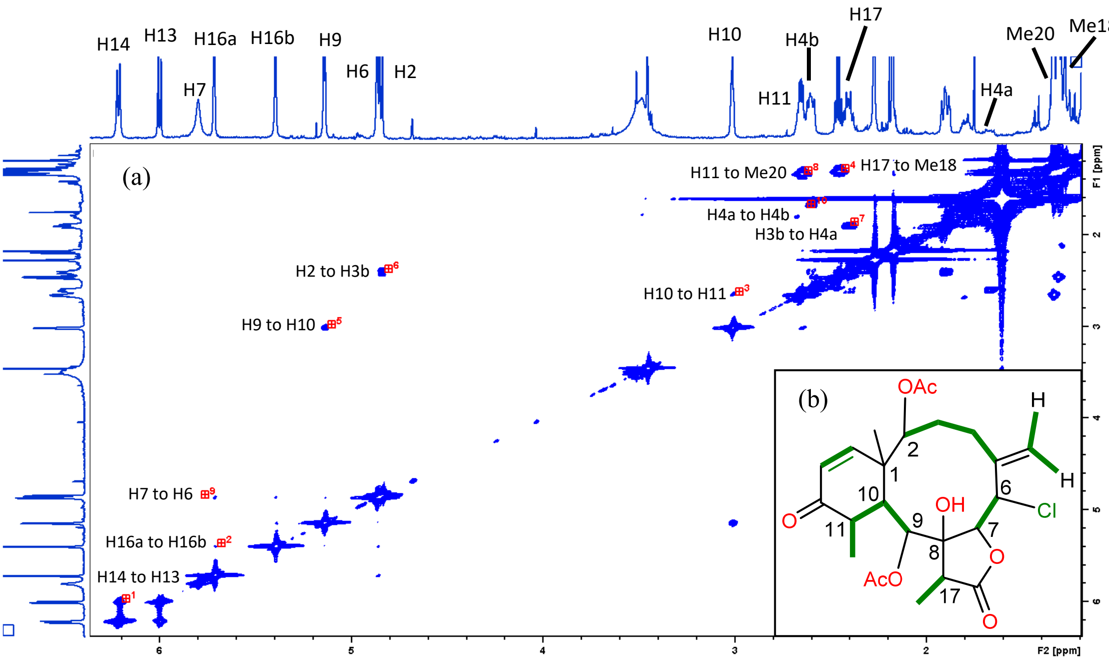

Supplementary Figure 18. Proton-proton correlation of 35 µg sample. a) COSY spectrum of briarane B-3 (35 µg) in a 1.7 mm NMR tube measured in a 700 MHz spectrometer. b) Main COSY correlations are indicated in green in the structure. (*cosyqf45*; NS: 64). Experimental time: 15 h.

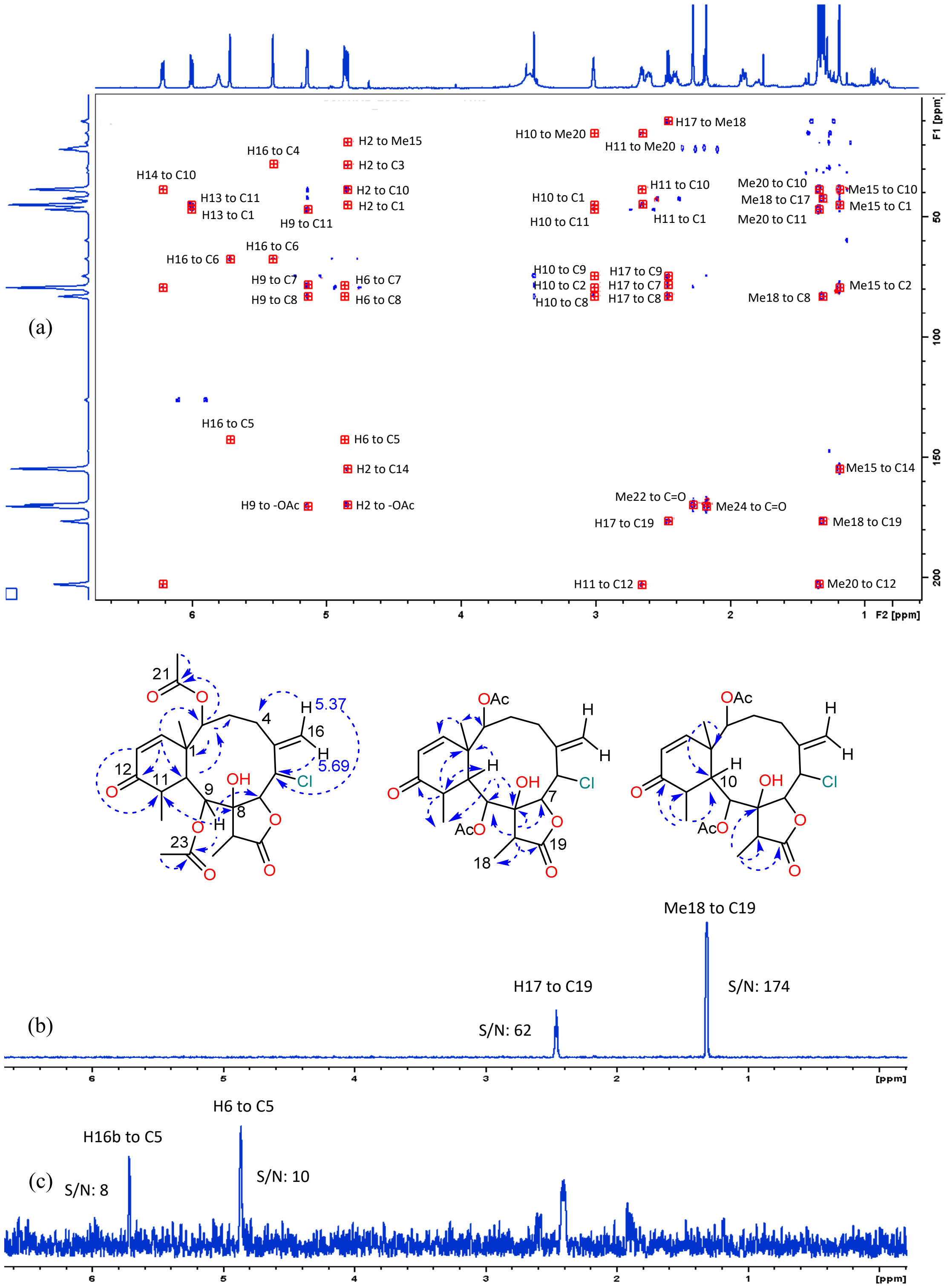
(a)
H17 to Me18
H10 to Me20
H11 to Me20
H2 to Me15
H16 to C4
H2 to C3
H14 to C10
H13 to C11
H13 to C1
H9 to C11
H2 to C10
H2 to C1
H10 to C1
H10 to C11
H11 to C10
H11 to C1
Me20 to C10
Me18 to C17
Me20 to C11
Me15 to C10
Me15 to C1
H16 to C6
H16 to C6
H9 to C7
H9 to C8
H6 to C7
H6 to C8
H10 to C9
H10 to C2
H10 to C8
H17 to C9
H17 to C7
H17 to C8
Me18 to C8
Me15 to C2
H16 to C5
H6 to C5
H2 to C14
Me15 to C14
H9 to -OAc
H2 to -OAc
Me22 to C=O
Me24 to C=O
H17 to C19
Me18 to C19
H11 to C12
Me20 to C12
F1 [ppm]
F2 [ppm]
5.37
5.69
OAc
AcO
OH
Cl
Me18 to C19
H17 to C19
S/N: 174
S/N: 62
(b)
[ppm]
H6 to C5
H16b to C5
(c)
S/N: 8
S/N: 10
[ppm]

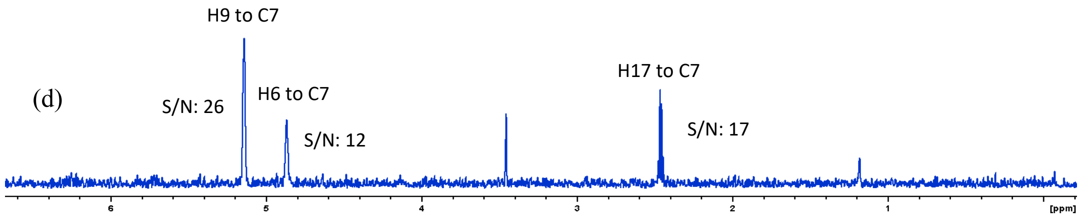

Supplementary Figure 19. Long range proton-carbon correlation of 35 µg sample. a) HMBC spectra for briarane B-3 (35 µg) measured in a Bruker spectrometer running at 800 MHz NMR spectrometer (*hmbcetgpnd;* NS: 248). All the key correlations are indicated in blue dashed arrow and selective traces showing proton correlation to C19, C5 and C7 (b, c and d panels, respectively). Signal to Noise ratio of selected HMBC correlations is indicated and labeled as S/N. Experimental time 62 h.

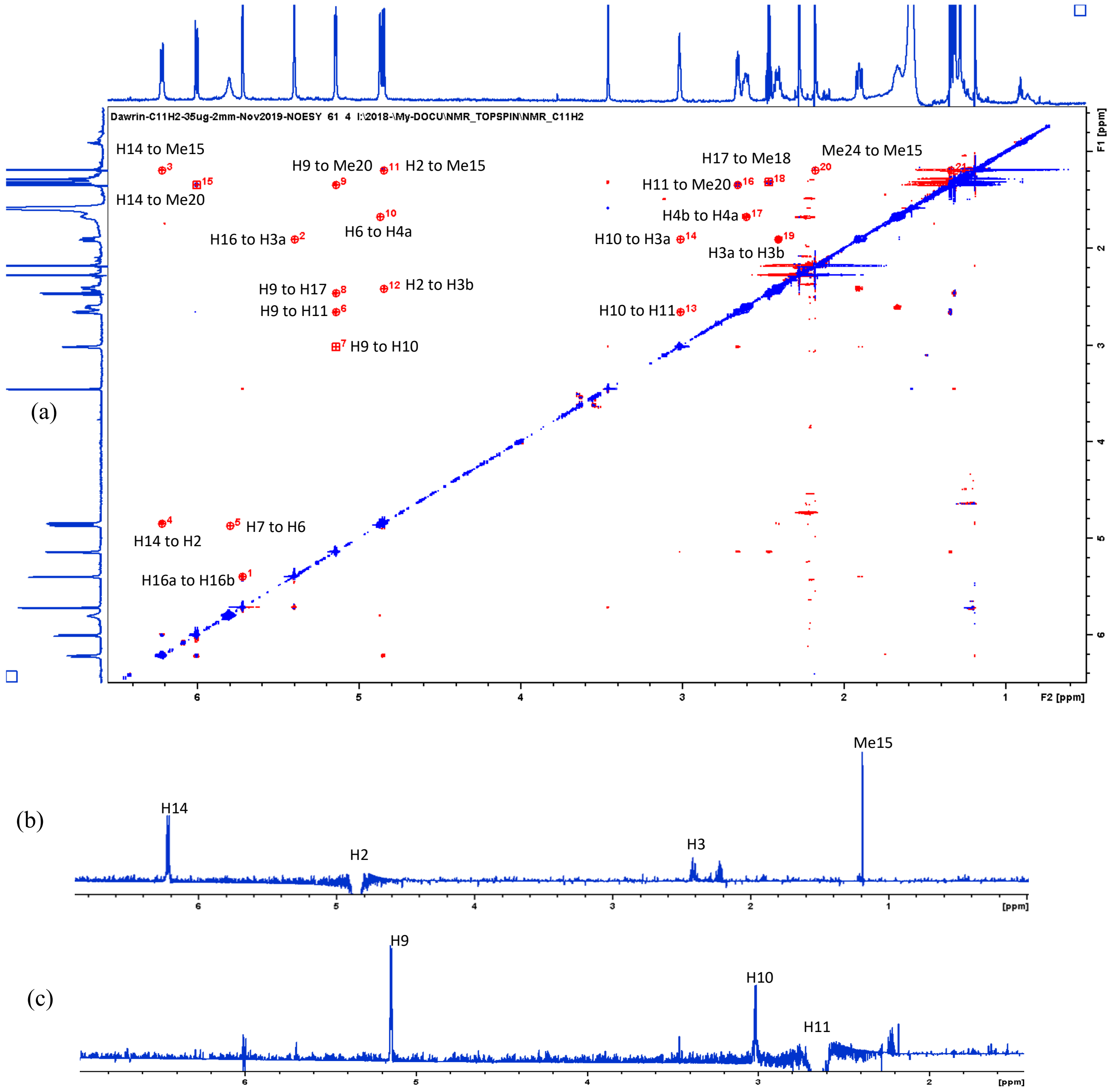

Supplementary Figure 20. Proton-carbon distance correlation of 35 µg sample. 2D NOESY spectra of briarane B-3 (35 µg) using a 2.0 mm NMR tube measured in Bruker spectrometer running at 800 MHz (a). Slice through the H2 (4.81 ppm) diagonal showing NOESY correlations to H14 (6.19 ppm), H3 (2.38 ppm) and Me15 (1.87 ppm) (b). Slice through the H11 (2.62 ppm) diagonal showing NOESY correlations to H9 (5.11 ppm) and H10 (2.99 ppm) (c). Mixing time: 420 ms. NUS parameters: 45 %/1 K/230. (*noesyetgp*; NS: 176). Experimental time: 68 h

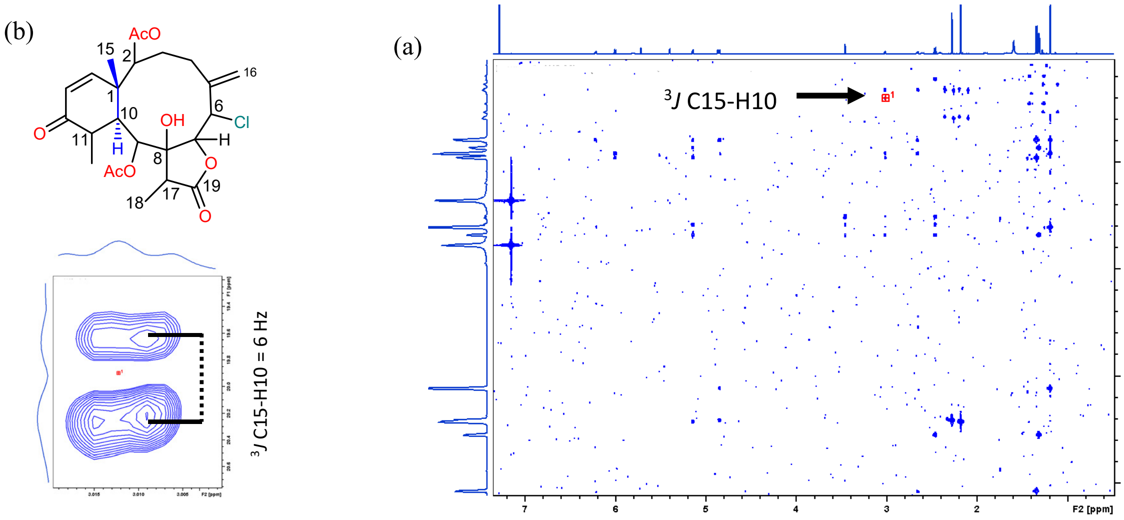

Supplementary Figure 21. Long range coupling measurement of 35 µg sample. *J*-HMBC spectrum of briarane B-3 (35 µg) sample using a 2.0 mm NMR tube recorded in a Bruker spectrometer running at 800 MHz (a). Inset shows coupling extraction of ^{3}JC15-H10 (6 Hz), blue marked in the structure, from the indirect dimension (b). Scaling factor used was 20. NUS parameters: 35 %/512/89. (*hmbcetgpjcl2nd*; NS: 576). Experimental time: 62 h.

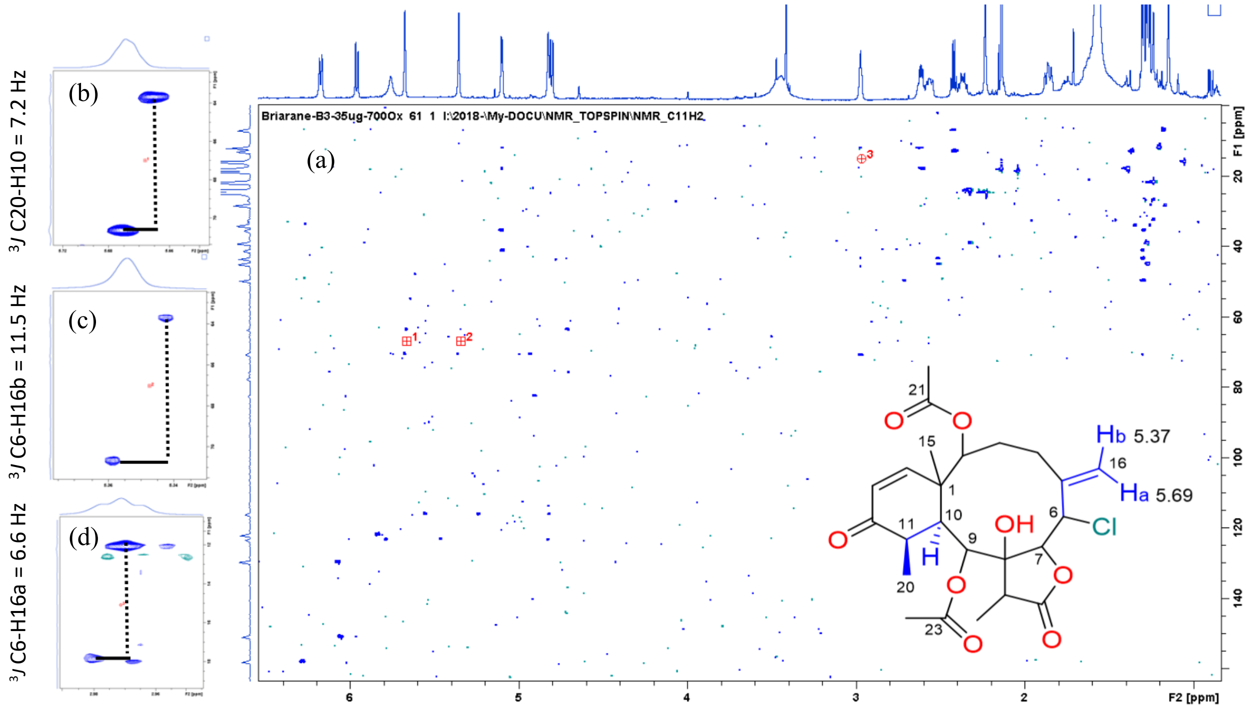

Supplementary Figure 22. Long range coupling measurement of 35 µg sample. HSQC-HECADE spectrum of briarane B-3 (35 µg), using a 2.0 mm NMR tube, recorded in a Bruker spectrometer running at 700 MHz (a). Inset shows coupling extraction of ^{3}JC6H16a, ^{3}JC6H16b and ^{3}JC20H10 (b, c, and d respectively); indicated in blue in the structure. TOCSY mixing time: 90 ms; $^{1}J_{CH}$ = 140 Hz and scaling factor of 8. (*hsqcdietgpjcndsisp*; NS: 160). Experimental time: 56 h.

Supplementary Note 10

Relative configuration analysis of briarane B-3 in PMMA-d_8 /$CDCl_3$ gel (2.2/1.8 mm)

NOE distance restraints establish relative configurations at C6, C7, C8, C9 and C17 as *S*, *R*, *R, S* and *R*, respectively, and leave the relative configurations at C1, C2, C10 and C11 undetermined. This provides a total of 16 absolute and 8 relative configurations. The strong NOE correlation between the methyl group at C1 and C11 indicates that they are located on the same side of the cyclohexanone ring. The absence of NOE correlations between H10 and the methyl groups at C1 and C11 seems to indicate an *anti*-disposition between H10/Me15 and H10/Me20. This was confirmed by a *J*-based configurational analysis that showed two large values of 7.2 and 6.0 Hz for $^{3}J(C_{20}\text{-}H_{10})$ and $^{3}J(C_{15}\text{-}H_{10})$, respectively. Since C1 and C10 must be either *RR* or S*S*, the four

possible relative configurations for those stereogenic centers are: *SRSR*, *SSSR*, *RRRS* and *RSRS* (C1, C2, C10, C11). The *RR* or *SS* configurations of C1 and C10 can also be further corroborated by the presence of medium sized HMBC correlations between carbon of the methyl at C15 and H10; carbon of the methyl group at C20 and H10 (see the 35 μg HMBC spectrum in **Supplementary** Figures 19, 21 and 22).

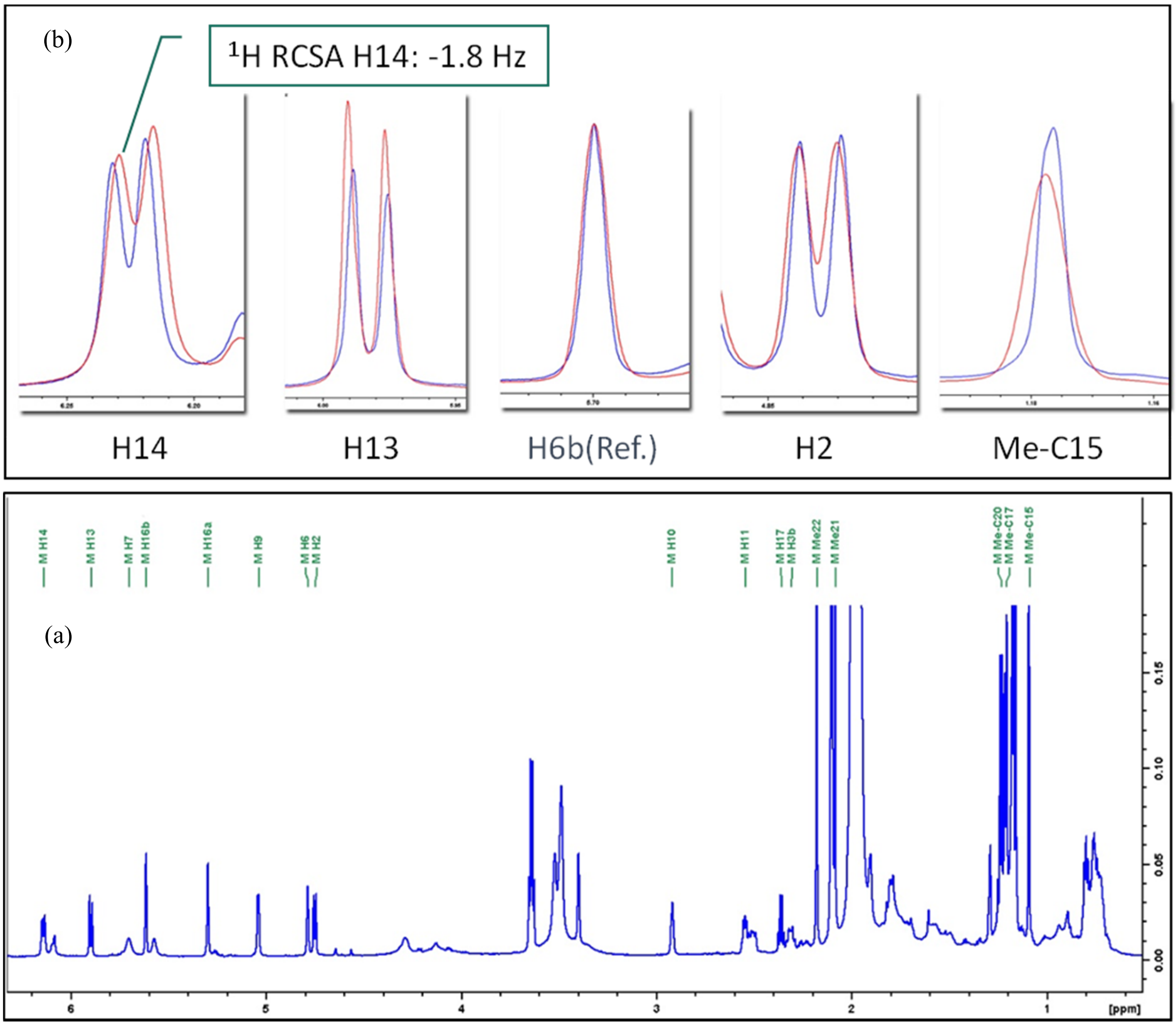

Supplementary Figure 23. Anisotropic proton NMR of 35 μg sample. 1D ^{1}H spectrum for a 35 μg sample of briarane B-3 in PMMA-d_8 (70/0.05) analyzed in a micro stretching device (2.2/1.8 mm) (a). ^{1}H RCSA data extraction for selective protons; blue and red represent isotropic and anisotropic measurement, respectively (b). Analysis was done in a Bruker spectrometer running at 800 MHz. (*zg*; NS: 1024).

Supplementary Table 15. ^{1}H RCSA data for the fitting of 6*S*,7*R*,8*R*,9*S*,17*R*,1*S*,2*S*,10*S*,11*R*-briarane B-3. Table shows experimental (^{1}H RCSA, exp (Hz)) and back calculated ^{1}HRCSA (^{1}H RCSA, calc (Hz), as well as the standard deviation of latest (σ^{1}H RCSA, calc) estimated from the Monte Carlo analysis, by using the $\boldsymbol{\sigma_{1H\ RCSA}}$ from Supplementary Equation (4).

Proton Number	^{1}H RCSA, exp (Hz)	^{1}H RCSA, calc (Hz)	σ^{1}H RCSA, calc (Hz)
**H14**	-1.7	-1.8	0.2
**H13**	1.1	1.0	0.2
**H6**	0.1	0.2	0.2
**H2**	0.9	0.7	0.2
**H10**	1.7	1.6	0.2
**H11**	2.7	2.6	0.2
**H4b**	1.1	1.0	0.2
**H17**	0.6	0.3	0.2
**Me15**	0.8	0.9	0.1
**Me-C22**	0.5	0.5	0.1
**Me20**	0.9	0.8	0.1
**Me18**	1.3	1.4	0.1
**Me-C21**	-0.6	-0.7	0.1

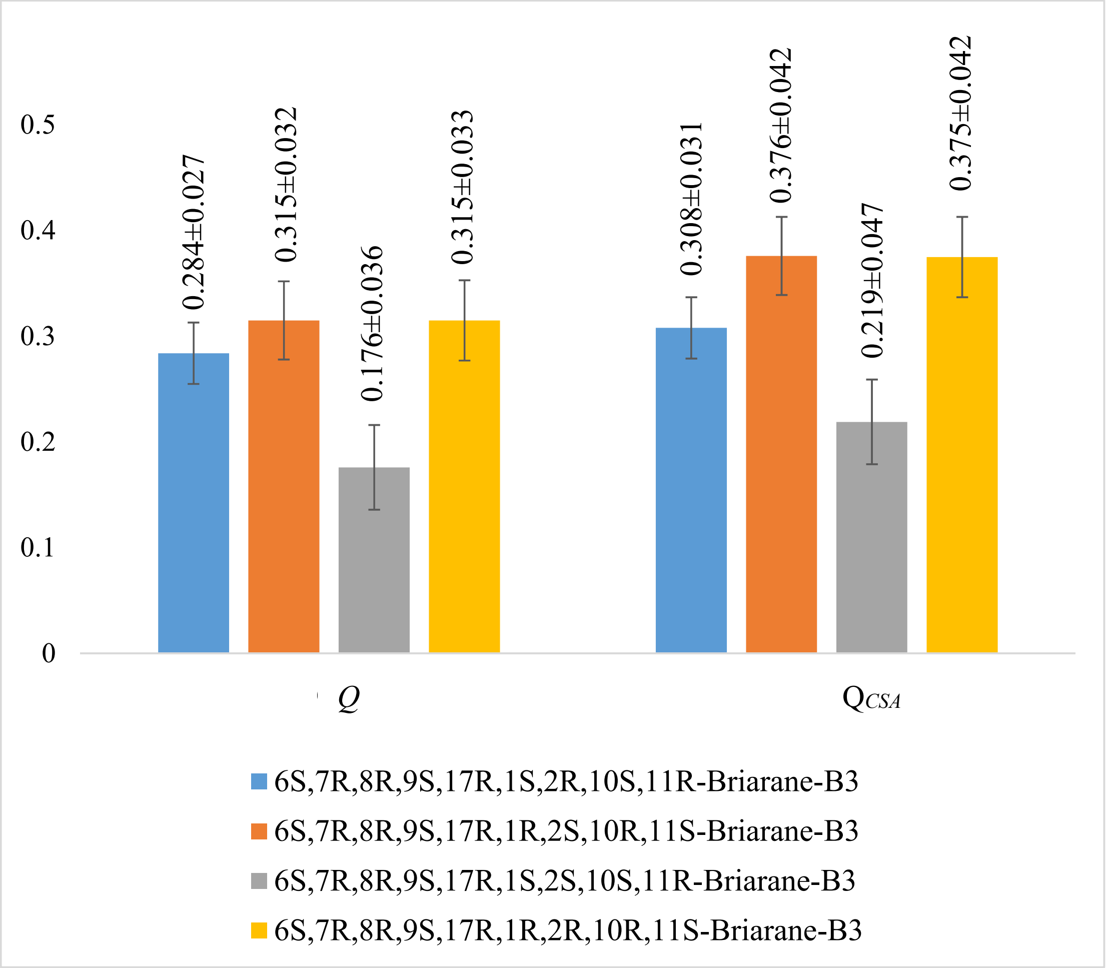

Supplementary Figure 24. Configuration analysis. Bar plot of Q and Q_{CSA} for the briarane B-3 ^{1}H RCSA fitting. The errors of the $Q(Q_{CSA})$ factors are expressed as the standard deviation. Standard deviation was computed by MSpin Montecarlo-module, using $\sigma_{^{1}H\ RCSA}$ from Supplementary Equation (4) in Hz shown in Supplementary Table 13.

Supplementary Note 11

ECD measurement:

ECD was measured in a JASCO J-815 CD spectromenter in acetonitrile, using a path length of the measuring cell of 1 mm and a sample concentration of 0.2 mg/mL. Population distributions used in ECD curve fitting is given in Supplementary Table 16. The ECD calculations were carried out by using IEFPCM[12] solvent model with acetonitrile as solvent.

Supplementary Table 16. ^{1}H-RCSA determined populations for different conformers of the configuration 6*S*,7*R*,8*R*,9*S*,17*R*,1*S*,2*S*,10*S*,11*R*-briarane B-3, which was used to simulate the ECD curve.

**Conformers**	**Population (%)**
C1	20.26
C3	16.19
C12	14.16
C13	11.11
C14	5.03
C20	10.10
C30	2.01
C33	3.01
C34	4.02
C39	10.10
C40	4.02

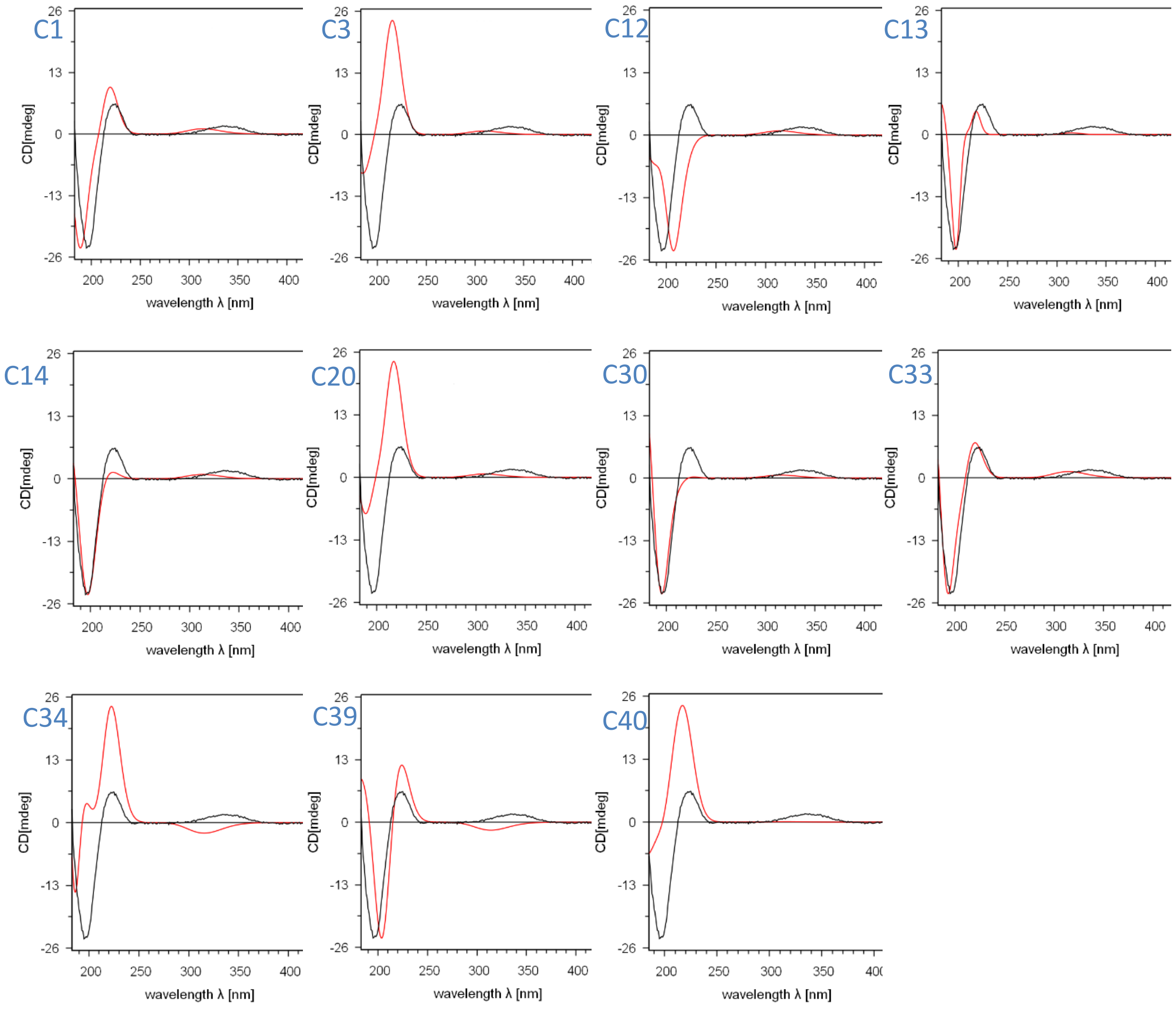

Supplementary Figure 25. ECD spectra of conformers. Calculated ECD (red line) for the different conformers 6*S*,7*R*,8*R*,9*S*,17*R*,1*S*,2*S*,10*S*,11*R*-briarane B-3 computed at TD-DFT/CAM-B3LYP/6-311++G(2d,p) and the experimental ECD spectrum (black line).

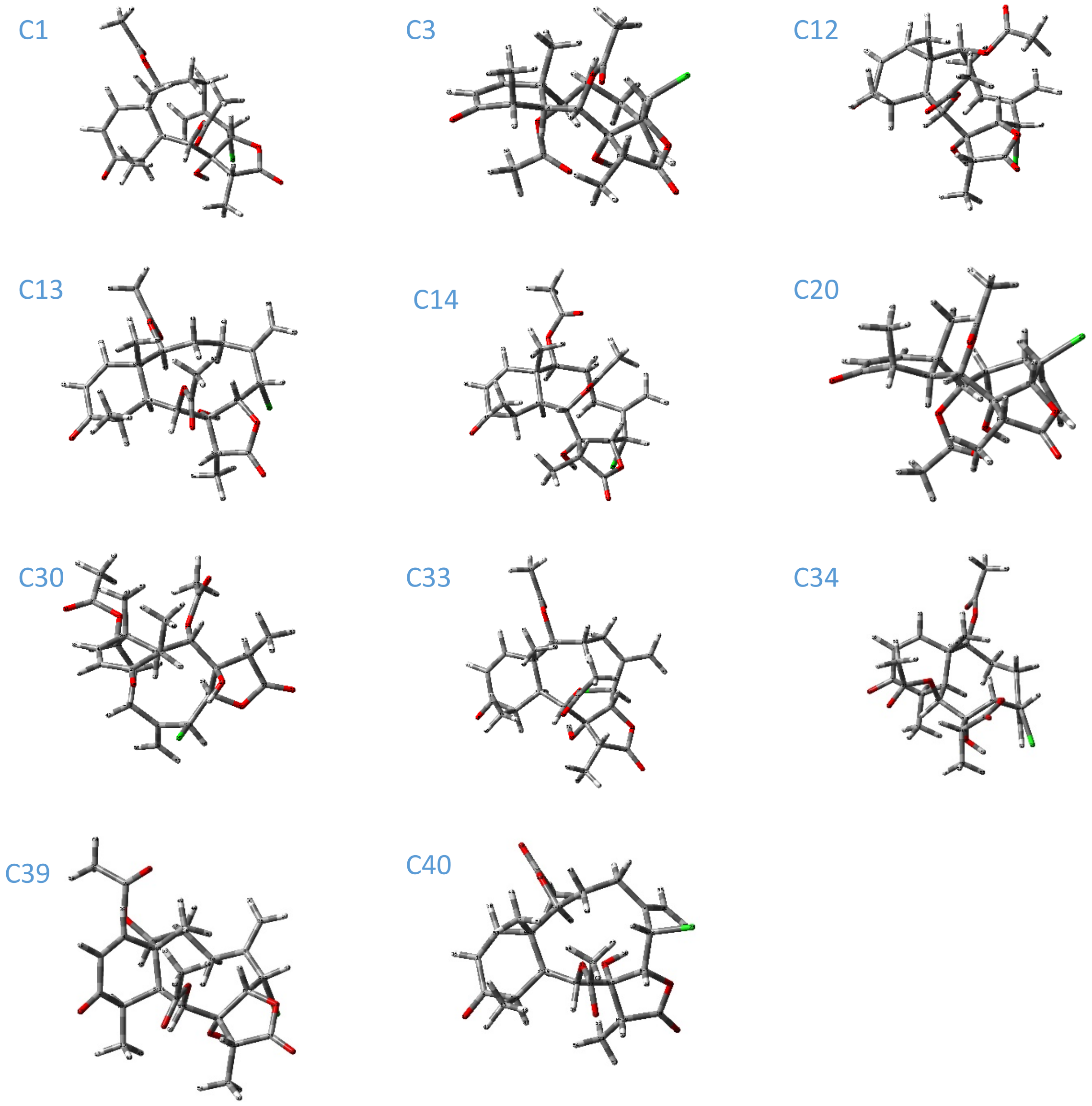

Supplementary Figure 26. 3D structures. Conformers of 6*S*,7*R*,8*R*,9*S*,17*R*,1*S*,2*S*,10*S*,11*R*-briarane B-3.

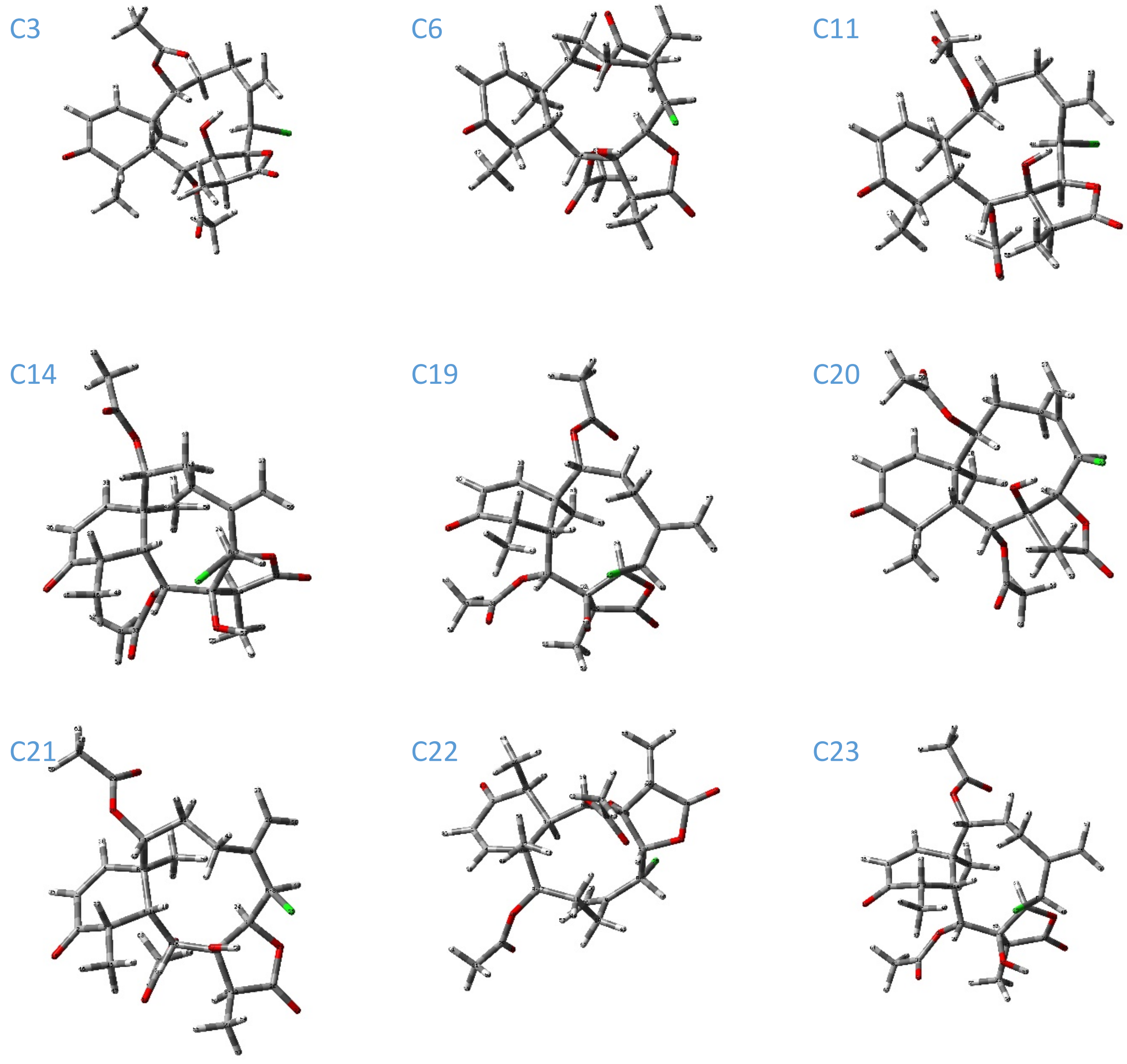

Supplementary Figure 27. 3D structures. Conformers of 6*R*,7*S*,8*S*,9*R*,17*S*,1*R*,2*R*,10*R*,11*S*-briarane B-3.

Supplementary Note 12

^{1}H RCSA analysis of 40 µg (-)-α-santonin at 600 MHz NMR spectrometer

The ^{1}H RCSA of (-)-α-santonin was measured for 40 µg sample amount using a Hilgenberg´s micro stretching device (2.2/1.8 mm). Experiments were performed in a 600 MHz NMR spectrometer equipped with a 5 mm cryo probe. For alignment PMMA-d_8 gel was used that has a cross-linker density of 0.009 %.

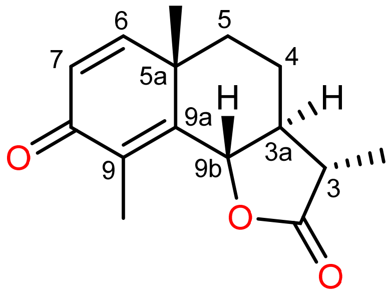

Structure of (-)-α-santonin

(-)-α-Santonin has 8 possible relative configurations and they were labelled via the *R* or *S* descriptors at C3, C3a, C5a and C9b, respectively. The correct structure has *SSSS* configuration. The initial structure of the configurations were built up in ChemDraw®. Structures were then optimized at B3LYP/6-31+G(d,p) level and chemical shift tensor were computed using GIAO method at DFT level B3LYP/6-31++g(2d,p) by using IEFPCM solvation model with $CHCl_3$ parameters.

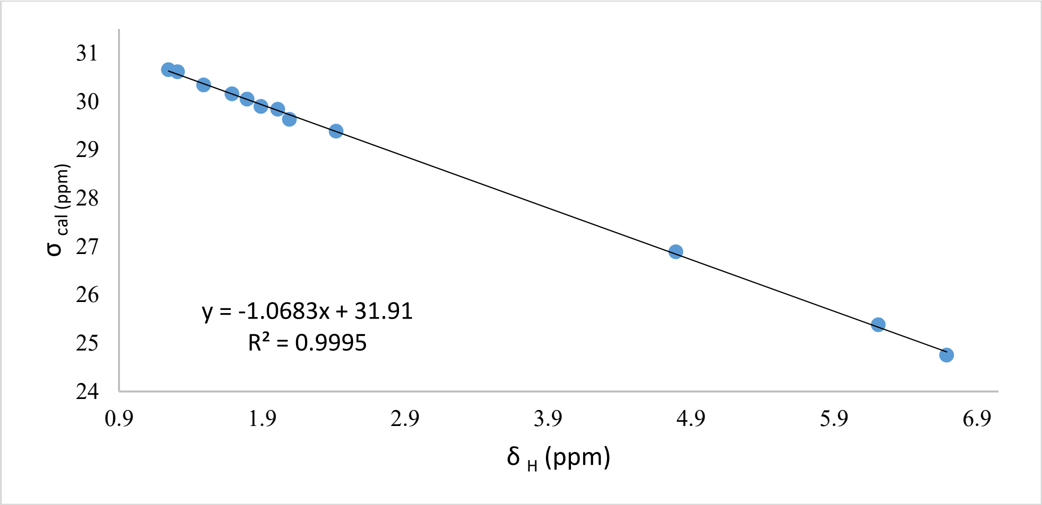

Supplementary Figure 28. Chemical shift correlation. Experimental ^{1}H chemical shifts (δ_H) of (-)-α-santonin and the calculated isotropic chemical shifts (σ_{cal}, not referenced) at DFT levels: GIAO/B3LYP/6-31++G (2d,p)/ IEFPCM ($CHCl_3$). Fitting curve equation and its coefficient of determination are shown in the figure.

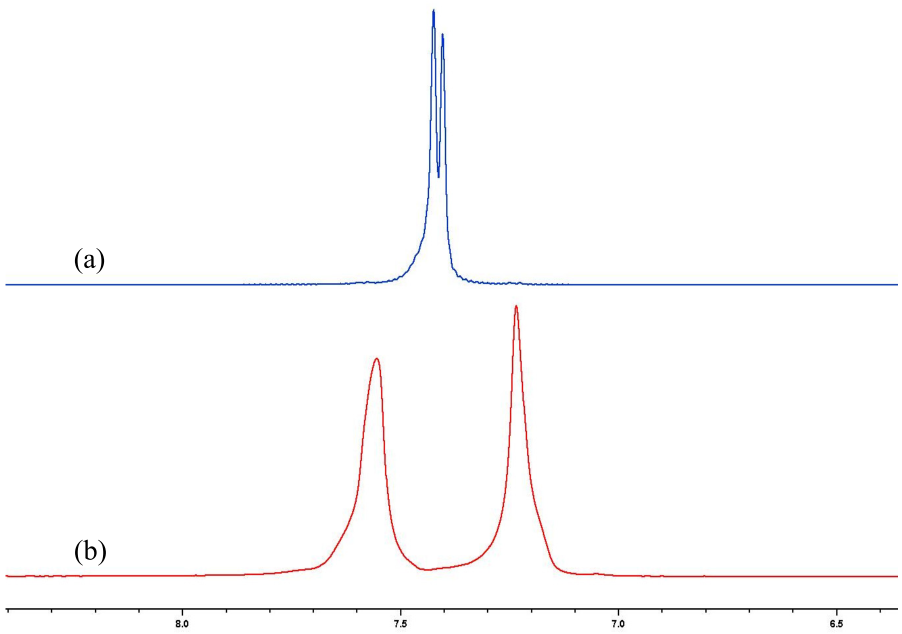

Supplementary Figure 29. Testing of alignment. Residual quadrupolar splitting of $CDCl_3$ in PMMA-d_8 gel when it was stretched inside 2.2- and 1.8-mm diameters of the device, a and b respectively. The deuterium splitting for the spectra from top to bottom in the figure are 1.6 and 27.7 Hz, respectively. Line width under anisotropic condition was 3.3 Hz. Recorded in a 600 MHz Bruker spectrometer.

(b)

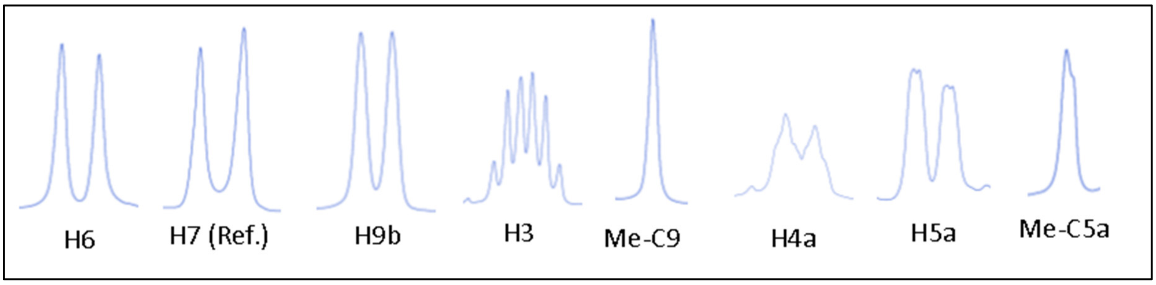

(a)

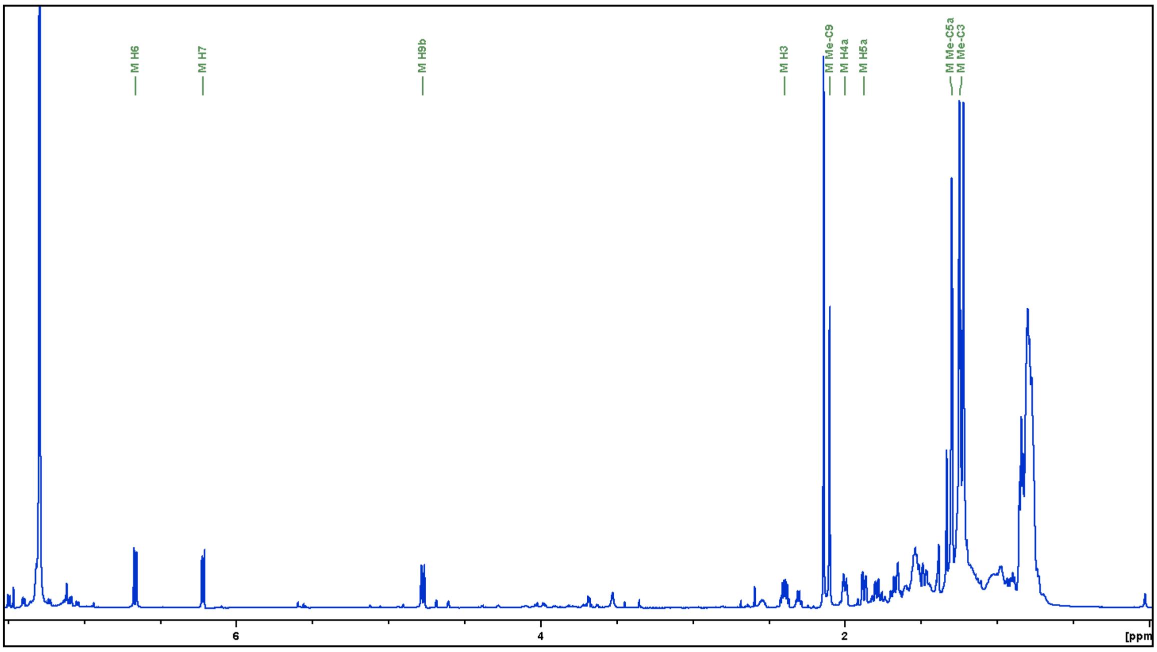

Supplementary Figure 30. Anisotropic proton NMR. 1D ^{1}H NMR spectrum of (-)-α-santonin (40 μg) when the gel was inside the 2.2 mm inner diameter of the device (a). The spectrum was acquired with a spin echo pulse sequence without *J* modulation (PROJECT).[26] Some of the resonances are shown in the top (b). (NS: 896)

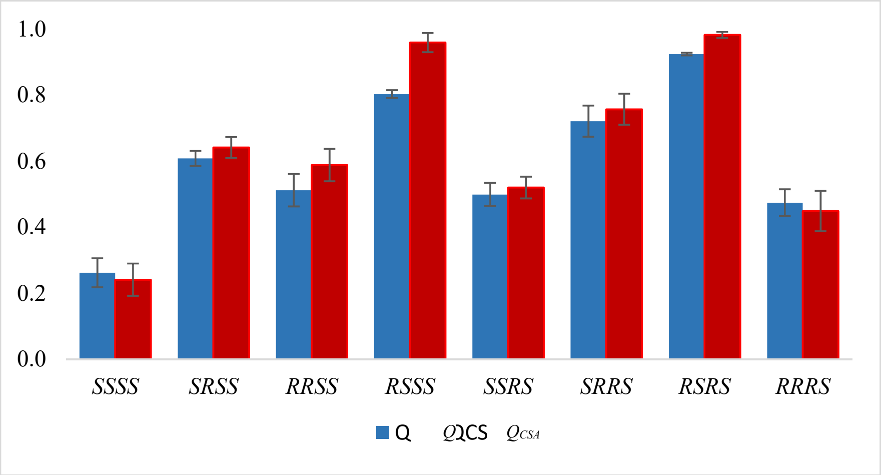

Supplementary Figure 31. Configuration analysis. The Q factors (blue bar) and Q_{CSA} factors (red bar) for (-)-α-santonin by ^{1}H RCSA analysis. The error bars represent the standard deviation.

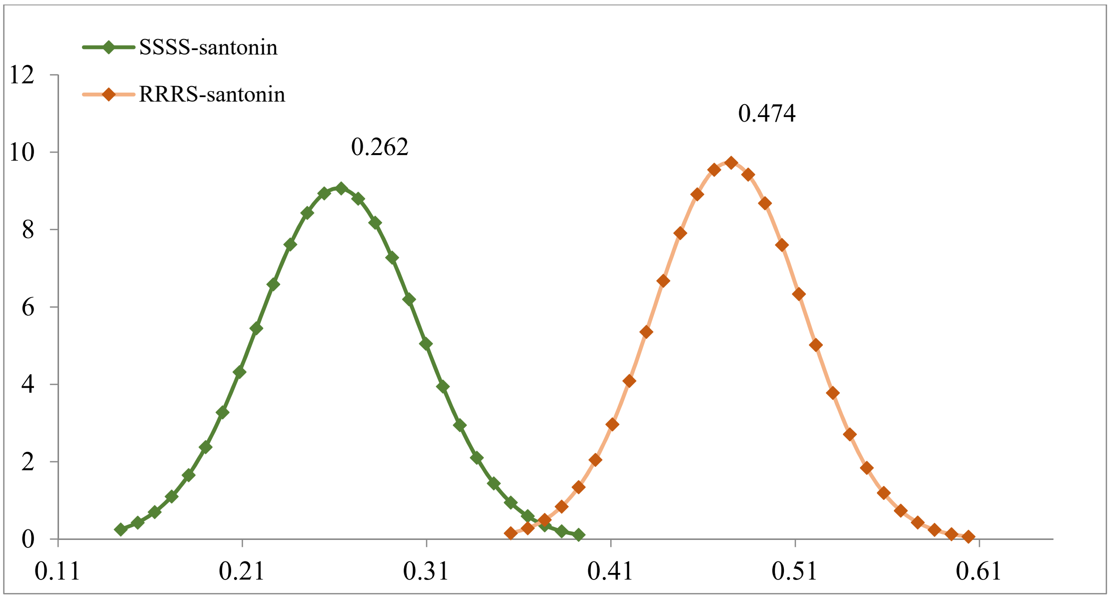

Supplementary Figure 32. Frequency polygons. Q frequency polygons of the closest wrong configuration of (-)-α-santonin (*RRRS*) compared to the correct one (i.e. *SSSS* configuration). Monte Carlo simulation was done using a Gaussian distribution with $\sigma_{1H\ RCSA}$ from Supplementary Equation (4) with 512 points. The plot was obtained from the Monte Carlo calculation that yields the standard deviations of the Qs.

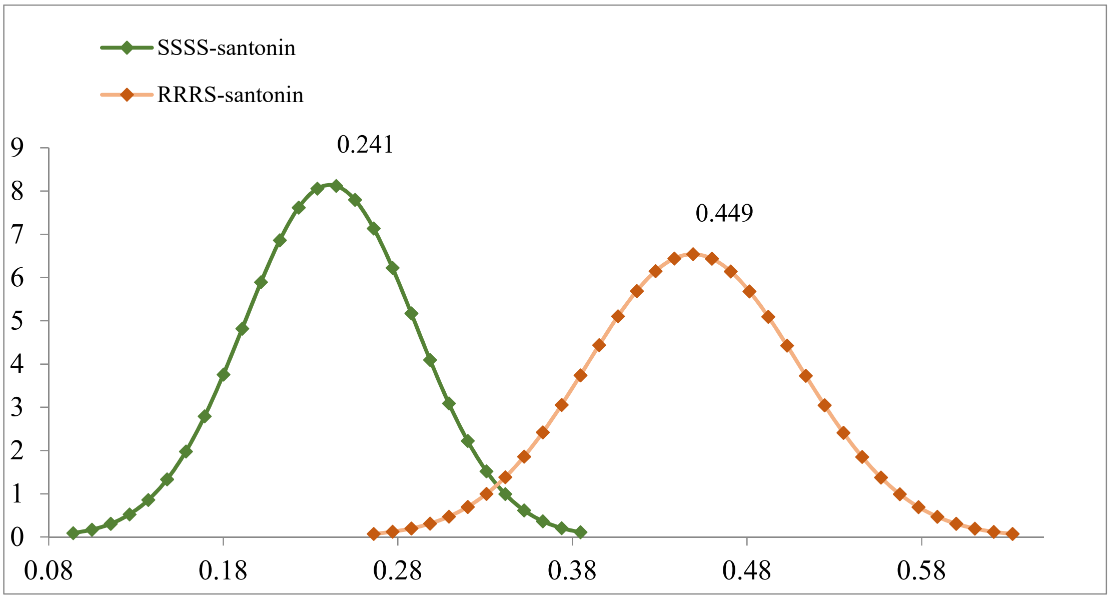

Supplementary Figure 33. Frequency polygons. Q_{CSA} frequency polygons of the closest wrong configuration of (-)-α-santonin (*RRRS*) compared to the correct one (i.e. *SSSS* configuration). Monte Carlo Simulation was done using a Gaussian distribution with $\sigma_{1H\ RCSA}$ from Equation (4) with 512 points. The plot was obtained from the Monte Carlo calculation that yields the standard deviations of the Q_{CSA}s.

Supplementary Note 13

^{1}H RCSA analysis of 45 μg brucine at 600 MHz NMR spectrometer

The ^{1}H RCSAs of brucine were measured for 45 μg sample amount using Hilgenberg's MSD with inner diameters of 2.2 and 1.8 mm. Experiments were performed in a 600 MHz NMR spectrometer equipped with a 5 mm cryo probe. In order to show the reusability of the deuterated PMMA gels, we used the same gel stick for brucine alignment that was previously used for (-)-α-santonin.
Although brucine can have 32 possible relative configurations, only 13 of them were energetically feasible. The configurations were labelled using the *R* or *S* descriptors at C7, C8, C12, C13, C14 and C16 respectively. For instance, *RSSRRS* stands for the correct configuration. The initial structures of each configuration were built up in ChemDraw®. Structures were then optimized at MPW1PW91/6-31+G(d,p) level and chemical shift tensors were computed using GIAO method at DFT level MPW1PW91/6-31+g(2d,p) by using IEFPCM solvation model with $CHCl_3$ parameters.

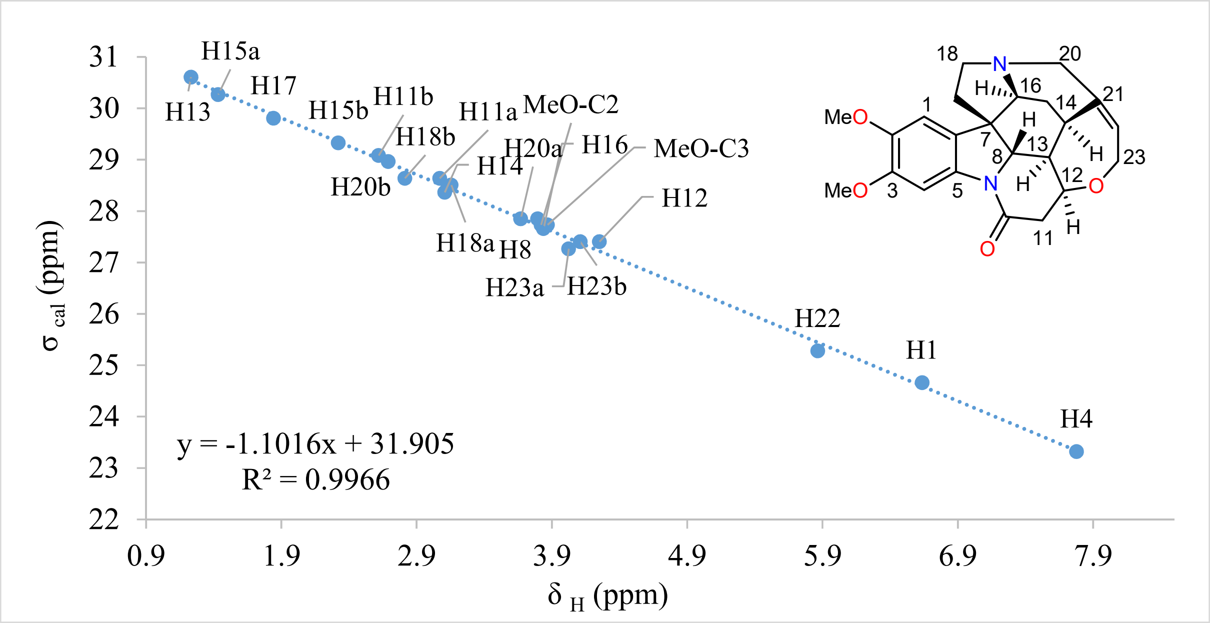

Supplementary Figure 34. Chemical shift correlation. Experimental ^1H chemical shifts (δ_H) and calculated isotropic chemical shifts (σ_{cal}, not referenced) at DFT level GIAO/MPW1PW91/6-31+G (2d,p)/ IEFPCM ($CHCl_3$) of brucine. Fitting curve equation and its coefficient of determination are also as insets

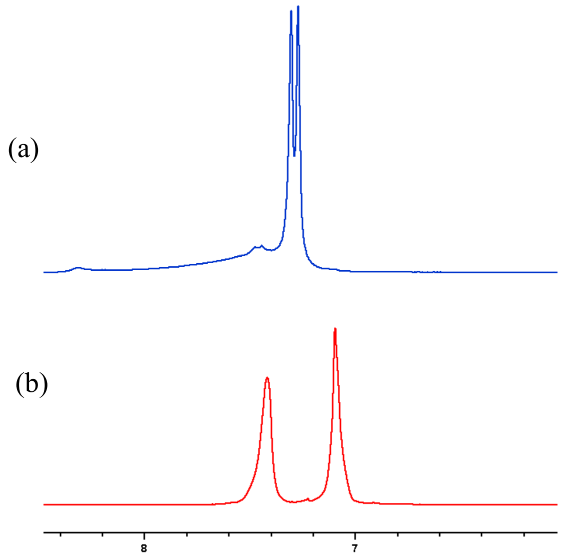

Supplementary Figure 35. Testing of alignment. Residual quadrupolar splitting of $CDCl_3$ in PMMA-d_8 gel when it was stretched inside 2.2- and 1.8-mm diameters, a and b respectively. The deuterium splitting

in the spectra from top to bottom in the figure are 2.9 and 29.6 Hz, respectively. Line width under the anisotropic condition was 5.2 Hz, measured in a 600 MHz Bruker spectrometer.

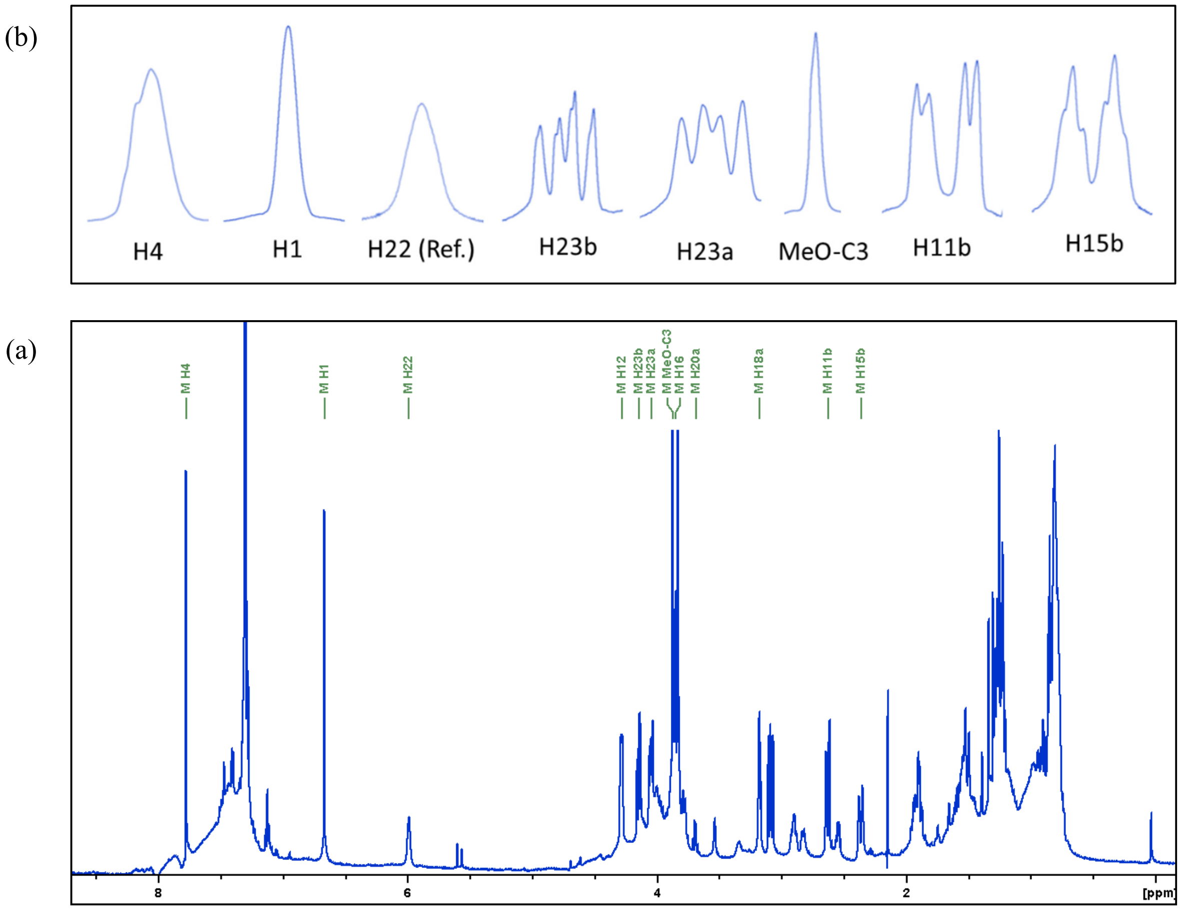

Supplementary Figure 36. Configuration analysis. 1D ^{1}H NMR spectrum of 45 µg brucine when the gel stick (PMMA-d_8) was inside the 2.2 mm inner diameter (a). The spectrum was acquired with spin echo pulse sequence without *J* modulation. Some of the selected resonances are shown in the top (b). (NS: 896).

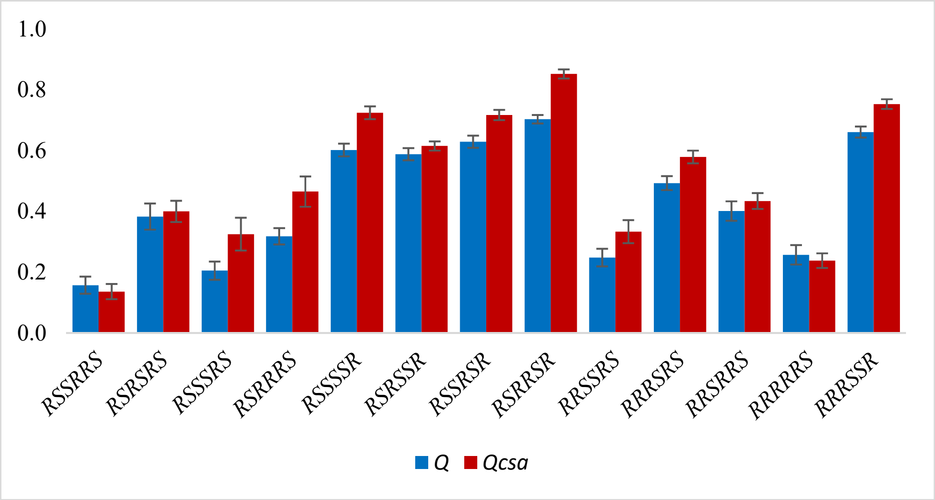

Supplementary Figure 37. Configuration analysis. The Q factors (blue bar) and Q_{CSA} factors (red bar) for brucine obtained from ^{1}H RCSA analysis. The error bars represent the standard deviations.

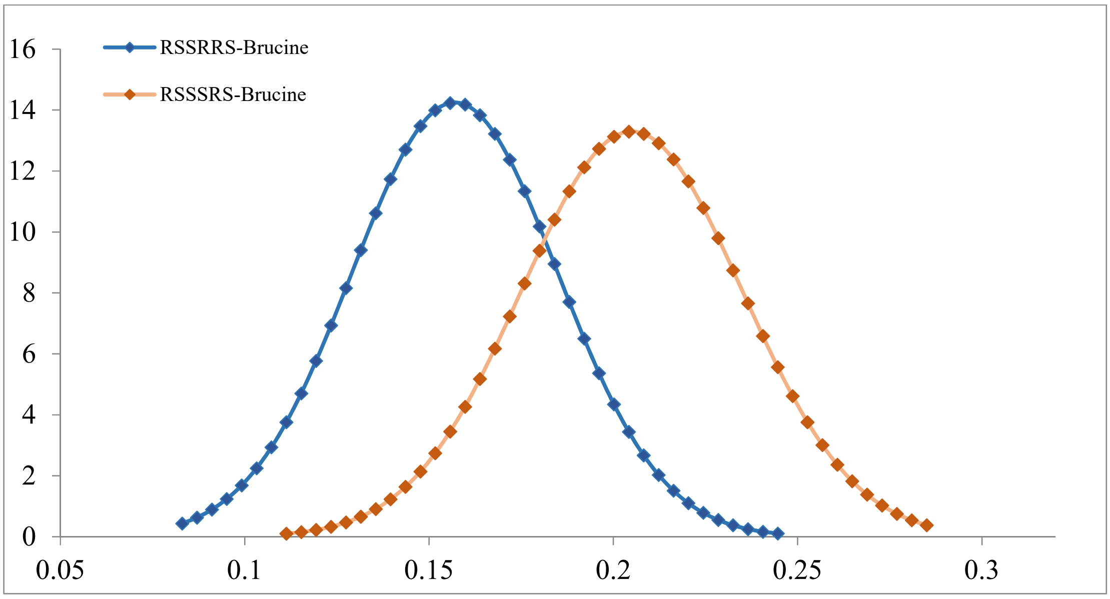

Supplementary Figure 38. Frequency polygons. Q frequency polygons of the closest configuration of brucine including the correct one (i.e. *RSSSRS* configuration). Simulation was done with 512 points and a Gaussian distribution with $\sigma_{1H\ RCSA}$ from Eq. S4. Using the Q values would not assign the configuration correctly beyond reasonable doubt. The plot was obtained from the Monte Carlo calculation that yields the standard deviations of the Qs.

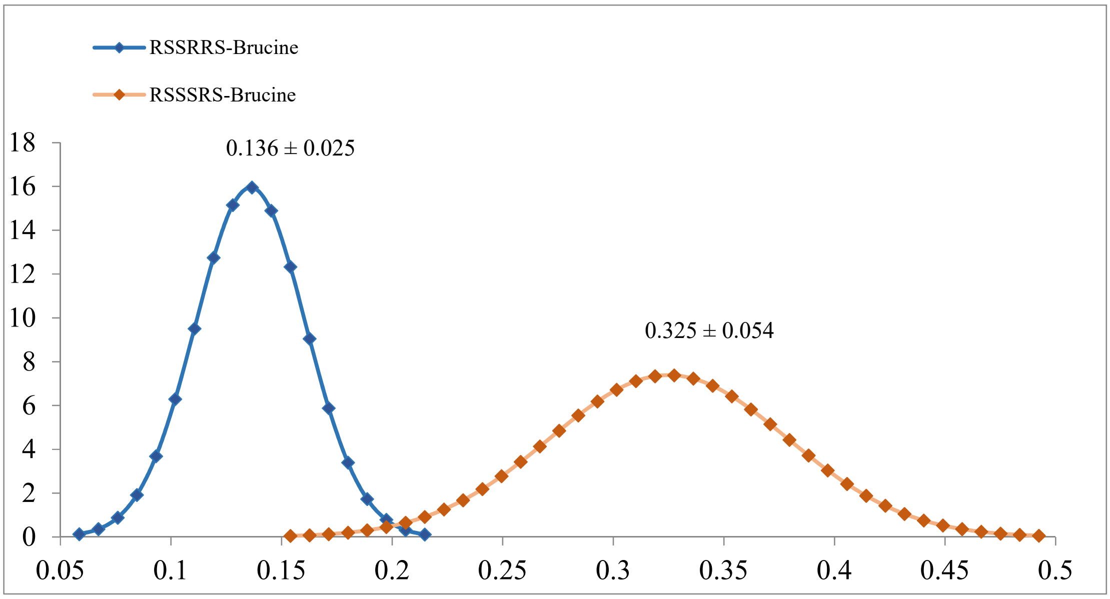

Supplementary Figure 39. Frequency polygons. Q_{CSA} frequency polygons of the closest configuration of brucine including the correct one (i.e. *RSSSRS* configuration). Simulation was done with 512 points and a Gaussian distribution with $\sigma_{1H\ RCSA}$ from (Equation 4). The Q_{CSA} values allow assignment of the configuration. The large difference in Q_{CSA} factor is due to large difference in CSA's of the H14 (i.e., CSA for H14 is 6.7 for *RSSRRS* configuration while it is 1.9 for *RSSSRS* configuration). The plot was obtained from the Monte Carlo calculation that yields the standard deviations of the Q_{CSA}s.

Supplementary Table 17. Proton residual chemical shift anisotropies standard deviation (Hz) associated with brucine data, computed with Eq. S4, and GDO calculated regardless of any error contribution.

Configuration	$\sigma\ {}^{1}H\ _{RCSA}$ (Hz)	GDO
RSSRRS-Brucine	0.244	2.11E-03
RSRSRS-Brucine	0.397	3.47E-03
RSSSRS-Brucine	0.260	2.25E-03
RSRRRS-Brucine	0.234	2.03E-03
RSSSSR-Brucine	0.211	1.82E-03
RSRSSR-Brucine	0.201	1.72E-03
RSSRSR-Brucine	0.215	1.85E-03
RSRRSR-Brucine	0.171	1.46E-03
RRSSRS-Brucine	0.251	2.17E-03
RRRSRS-Brucine	0.208	1.79E-03
RRSRRS-Brucine	0.287	2.49E-03
RRRRRS-Brucine	0.276	2.39E-03
RRRSSR-Brucine	0.205	1.77E-03

Supplementary Note 14

Monte Carlo analysis

We have evaluated the impact for experimental and CSA error on the Q and Q_{CSA} values obtained from the SVD fitting by using a "Montecarlo" module within the MSpin software. First, an appropriate amount of simulated RCSA data sets were generated by sampling a Gaussian distribution centered on each experimental RCSAs with a standard deviation generated as described in Supplementary Equation (4) ($\sigma\ {}^{1}H\ RCSA$) (See page S18-21). Then, a SVD fitting was performed for each set and the back predicted values along with quality factors Q were averaged over all the different computations. Finally, corresponding standard deviations were computed. The frequency polygons for the two configurations for each molecule having lowest Q factors are depicted below.

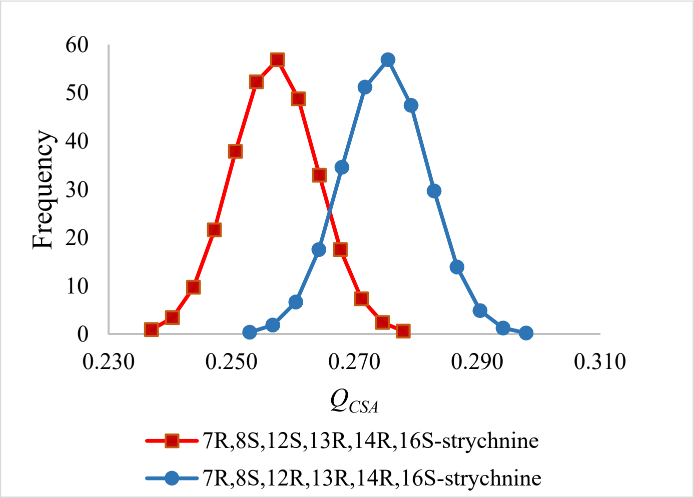

Supplementary Figure 40. Frequency polygons. Monte Carlo analysis for the ^{1}H RCSA analysis for the 8 mg strychnine sample collected in PMMA gel. Q_{CSA} frequency polygons of 7*R*,8*S*,12*S*,13*R*,14*R*,16*S*-strychnine (incorrect configuration: blue circles) and 7*R*,8*S*,12*R*,13*R*,14*R*,16*S*-strychnine (correct configuration: red square). Gaussian distribution with $\sigma\ {}^{1}H\ RCSA$ from Supplementary Equation (4) was used with a sample size of 480. The plot was obtained from the Monte Carlo calculation that yields the standard deviations of the Q_{CSA}.

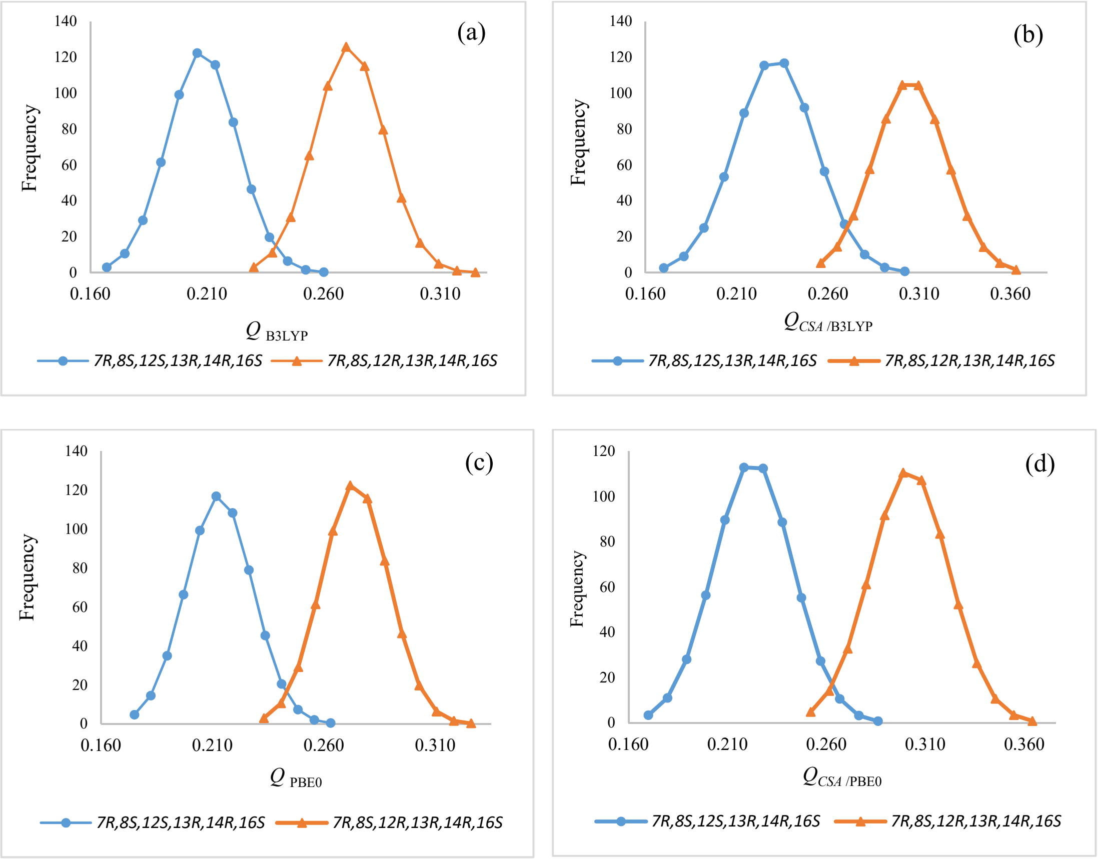

Supplementary Figure 41. Frequency polygons. Monte Carlo analysis for the ^{1}H RCSA analysis for the 80 µg strychnine sample collected in PMMA-d_8 gel. Q and Q_{CSA} frequency polygons of 7*R*,8*S*,12*S*,13*R*,14*R*,16*S*-Strychnine (blue circles) and 7*R*,8*S*,12*R*,13*R*,14*R*,16*S*-Strychnine (orange triangles) computed at levels GIAO/B3LYP/6-311+G(2d,p)/IEFPCM (a and b) and CSGT/PBE0/cc-pVTZ/COSMO (c and d). Solvent parameters used in the calculations were for chloroform. Sample size 600. The error of the RCSAs is derived from the $\sigma_{^{1}H\ RCSA}$ from Supplementary Equation (4). The plot was obtained from the Monte Carlo calculation that yields the standard deviations of the Qs.

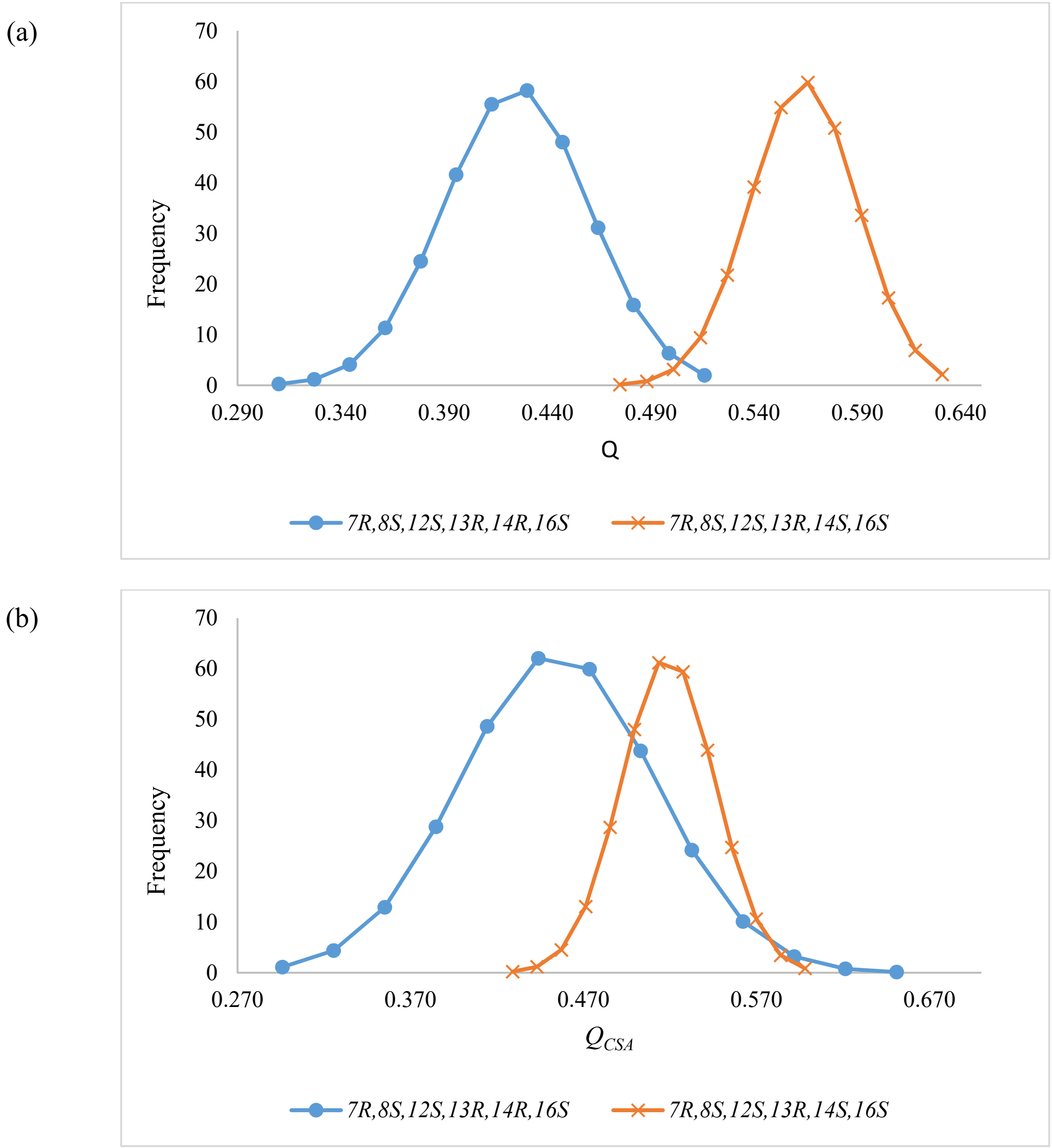

Supplementary Figure 42. Frequency polygons. Q (a) and Q_{CSA} (b) frequency polygons of 7*R*,8*S*,12*S*,13*R*,14*R*,16*S*-strychnine (blue curve) and 7*R*,8*S*,12*S*,13*R*,14*S*,16*S*-strychnine (orange curve), of a 10 µg sample analyzed in a Hilgenberg´s micro stretching device of 2.2/1.8 mm (PMMA-d_8). CSA was computed at GIAO/B3LYP/6-311+G(2d,p)/IEFPCM level of theory. Solvent parameters used in the calculations were for chloroform. Gaussian distribution with $\sigma_{^1H\,RCSA}$ from Supplementary Equation (4) was used in the Monte Carlo analysis with a sample size of 300. The plot was obtained from the Monte Carlo calculation that yields the standard deviations of the Qs.

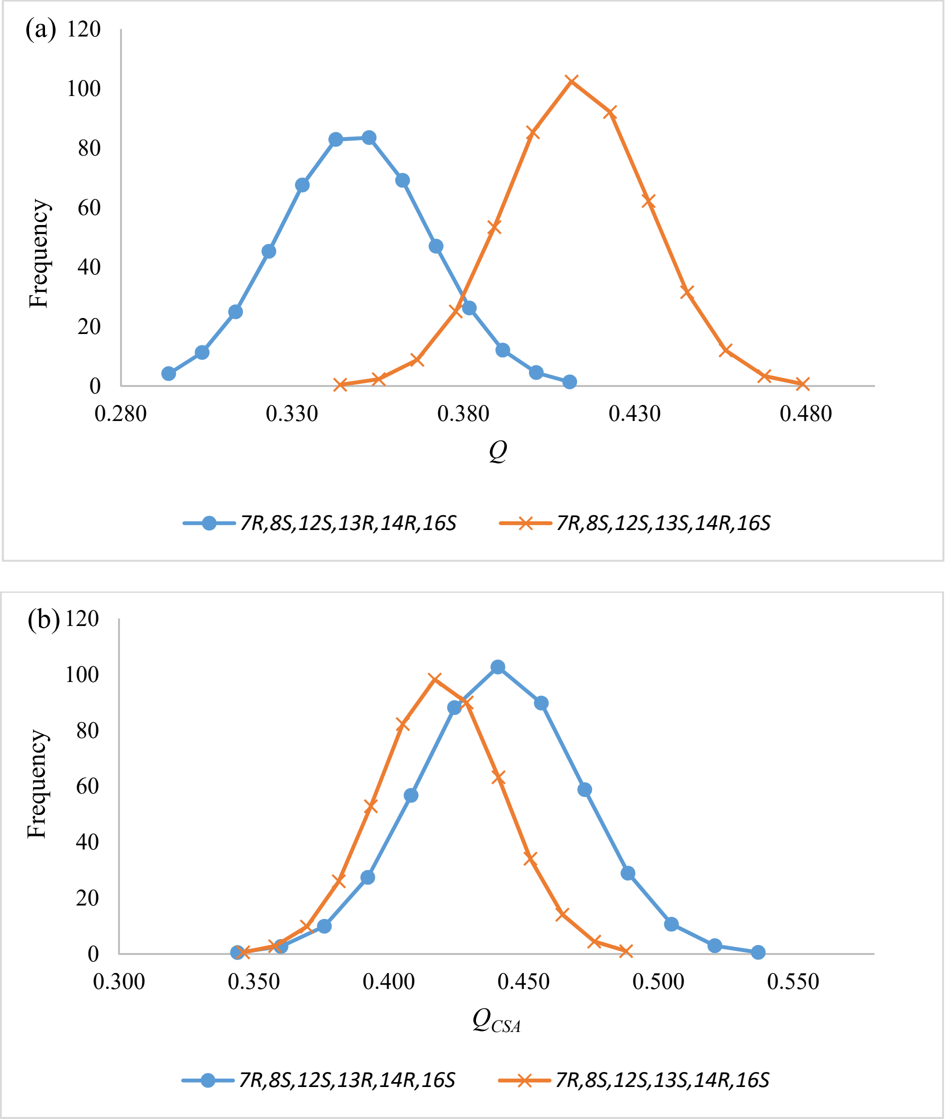

Supplementary Figure 43. Frequency polygons. Q (a) and Q_{CSA} (b) frequency polygons of 7*R*,8*S*,12*S*,13*R*,14*R*,16*S*-strychnine (blue line) and 7*R*,8*S*,12*S*,13*S*,14*R*,16*S*-strychnine (orange line), analyzed in liquid crystal, computed at GIAO/B3LYP/6-311+G(2d,p)/IEFPCM level of theory. Solvent parameters used in the calculations were for chloroform. Gaussian distribution with $\sigma_{^1H\ RCSA}$ from Supplementary Equation (4) was used during Monte Carlo study with a sample size of 480. The plot was obtained from the Monte Carlo calculation that yields the standard deviations of the Qs.

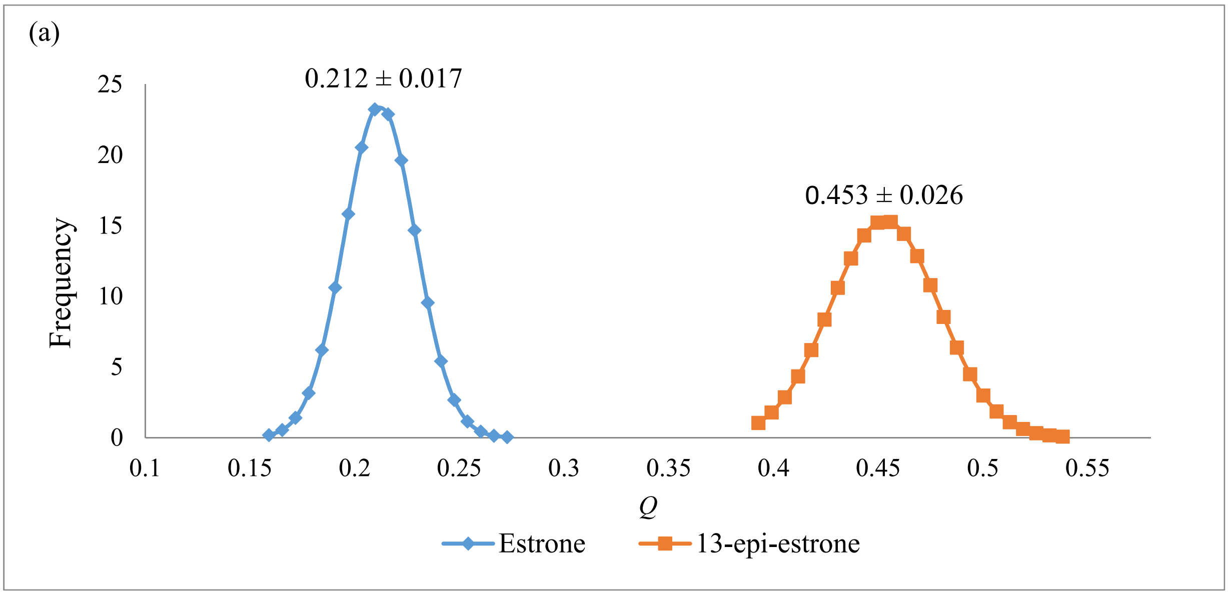

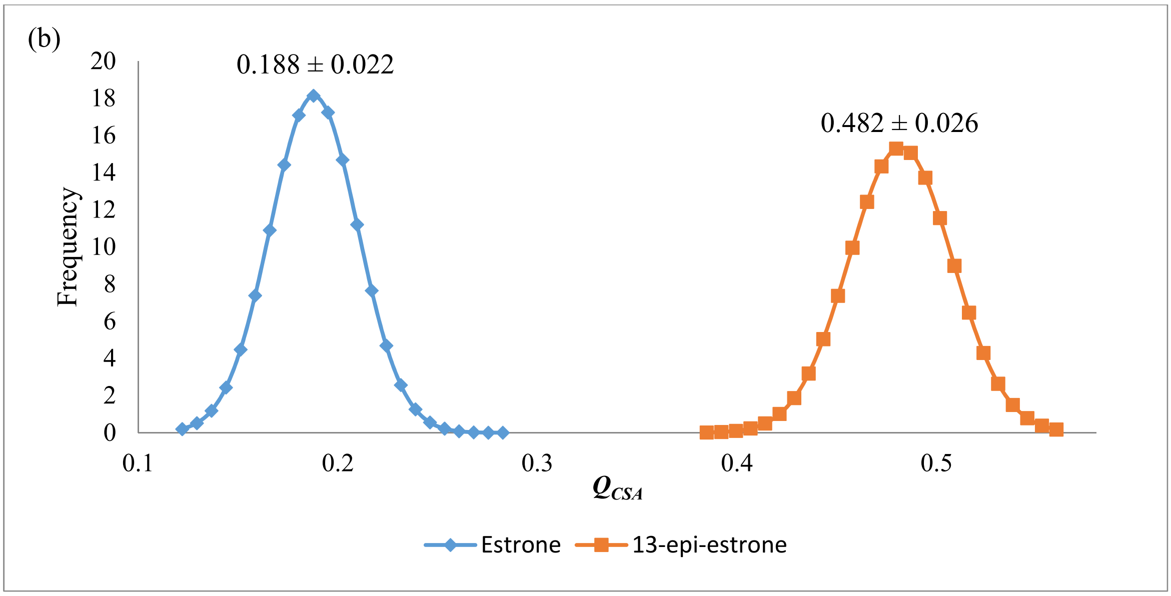

Supplementary Figure 44. Frequency polygons. Q (a) and Q_{CSA} (b) frequency polygons of estrone (blue line) and 13-*epi*-estrone (orange line) computed at GIAO/B3LYP/6-311+G(2d,p)/IEF-PCM level of theory. Solvent parameters used in the calculations were for DMSO. Gaussian distribution with $\sigma_{\ ^{1}H\ RCSA}$ from Supplementary Equation (4) was used during Monte Carlo study with a sample size of 512. The plot was obtained from the Monte Carlo calculation that yields the standard deviations of the Qs.

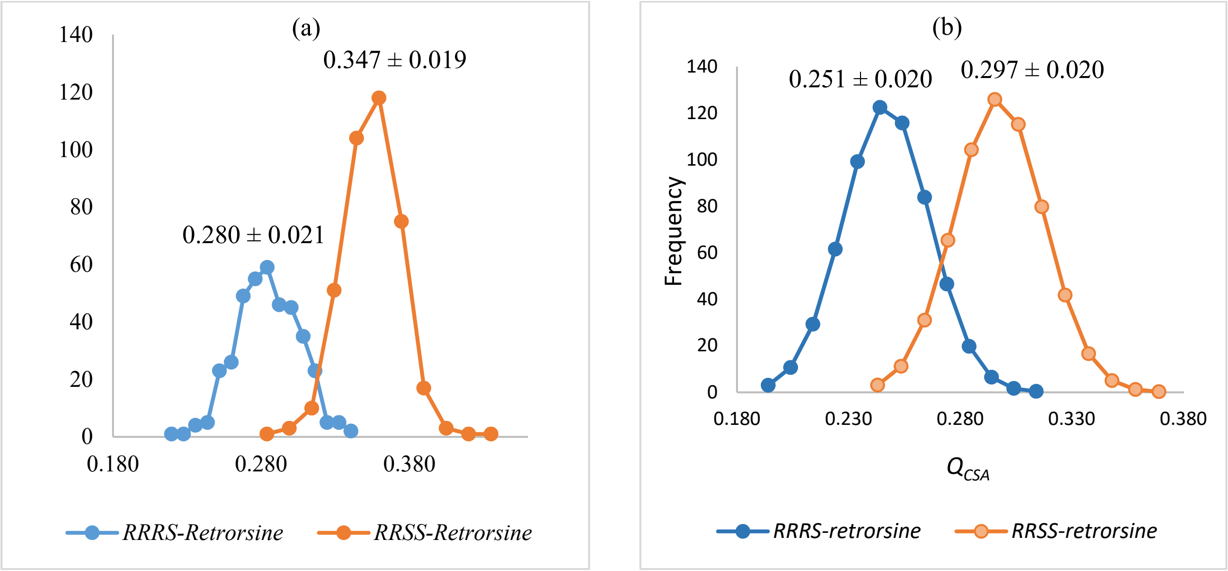

Supplementary Figure 45. Frequency polygons. Monte Carlo simulation of Q (a) and Q_{CSA} (b) for *RRRS*-retrorsine (correct configuration) and *RRSS*-retrorsine (next best incorrect configuration). A Gaussian distribution with $\sigma_{^1H\ RCSA}$ from Supplementary Equation (4) was used during the simulation, with a sample size of 384. The plot was obtained from the Monte Carlo calculation that yields the standard deviations of the Qs.

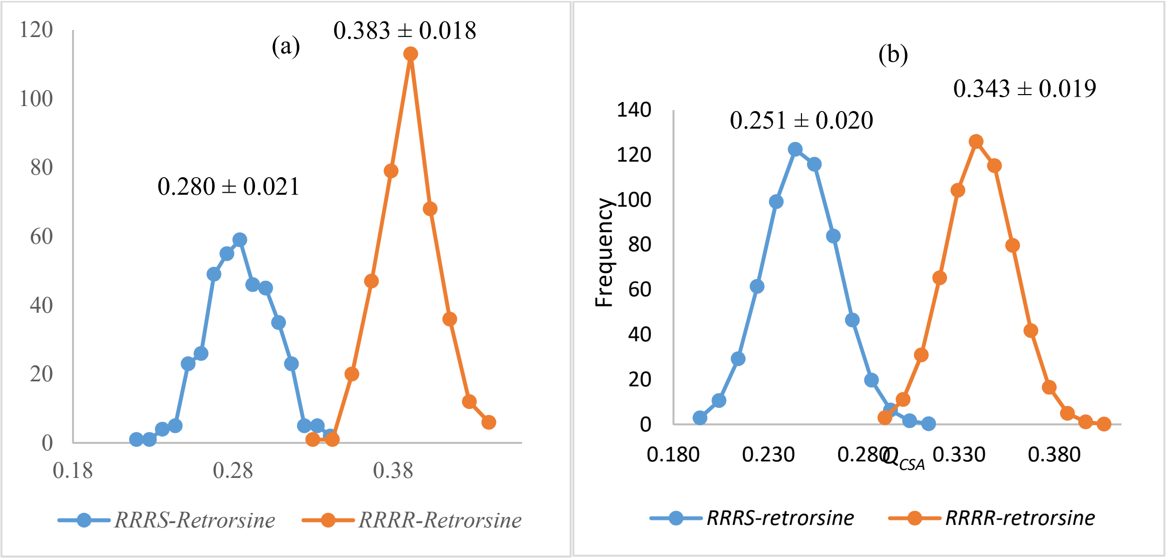

Supplementary Figure 46. Frequency polygons. Monte Carlo simulation of *RRRR*-retrorsine and (a) *RRRS*-retrorsine (b). A a Gaussian distribution with $\sigma_{^1H\ RCSA}$ from Supplementary Equation (4)

was used during the Monte Carlo simulation in all the cases, with a sample size of 384. The plot was obtained from the Monte Carlo calculation that yields the standard deviations of the *Q*s.

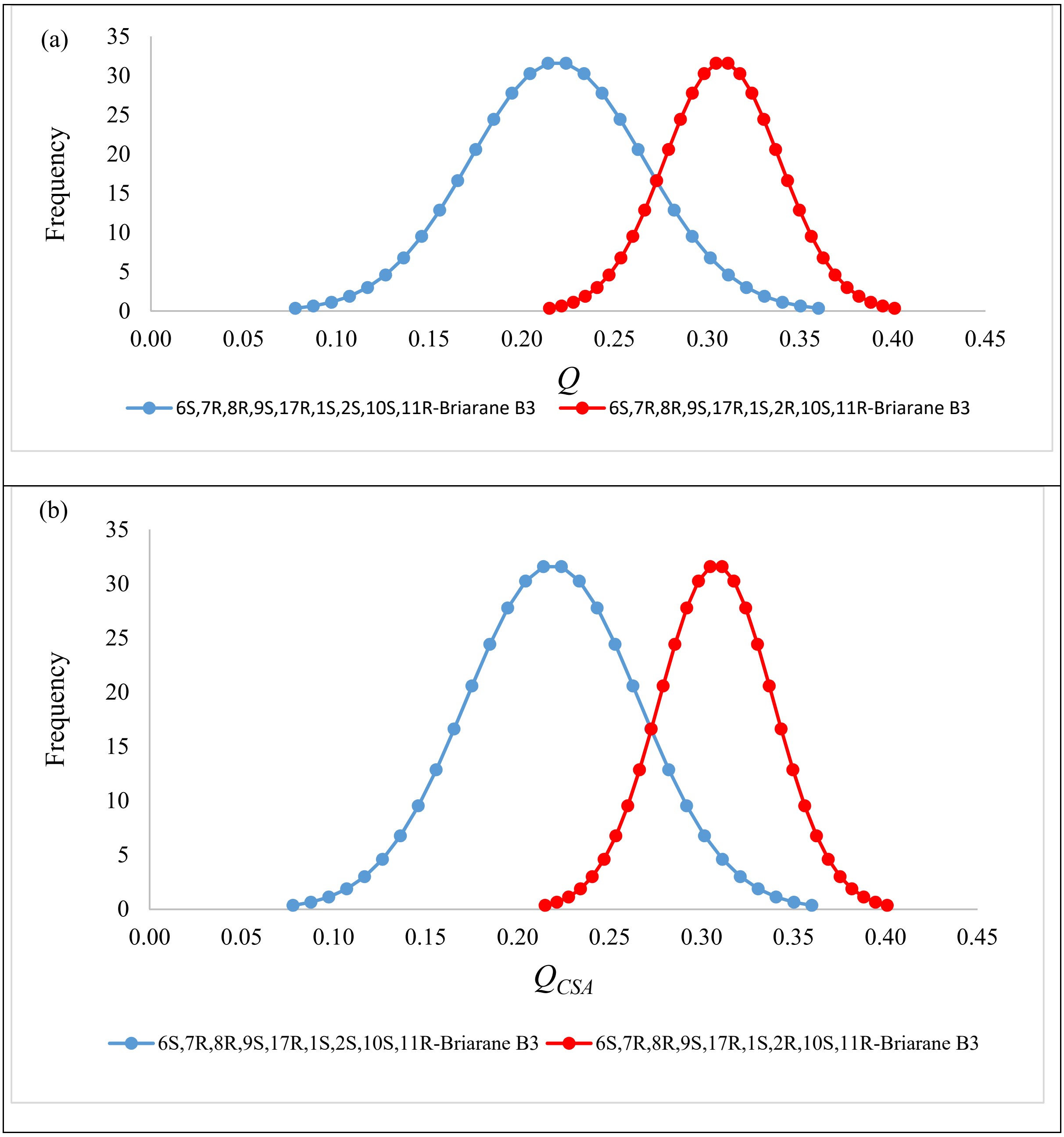

Supplementary Figure 47. Frequency polygons. Monte Carlo simulation of Q (a) *and* Q_{CSA} (b) for 6*S*,7*R*,8*R*,9*S*,17*R*,1*S*,2*S*,10*S*,11*R*-briarane B-3 and the next best configuration 6*S*,7*R*,8*R*,9*S*,17*R*,1*S*,2*R*,10*S*,11*R*-briarane B-3. Gaussian distribution with $\sigma\ {}^{1}H\ RCSA$ from Supplementary

Equation (4) was used during the Monte Carlo simulation in all the cases, with a sample size of 384. The plot was obtained from the Monte Carlo calculation that yields the standard deviations of the *Q*s.

Supplementary Note 15

DP4+ analysis

DP4+ analysis is complementary to anisotropic NMR and the confidence in the result increases when both reach the same conclusion. Therefore, we have also performed a DP4+ analysis for the briarane B-3 using the reported procedure in the literature.[25,20] In DP4+ analysis, the error is converted into probabilities through scaled and unscaled chemical shifts; and configuration assignment is reliable only when the DP4+ probability is 95% or above. For DP4+ probability calculation, we have used the conformers for each configuration which were selected by the analysis of the ^{1}H RCSA data. The populations of the selected conformers were obtained by free energies calculated at B3LYP/6-31+G(d,p) geometries and shielding tensors were calculated using GIAO/B3LYP/6-311+G(d,p) using $CDCl_3$ solvent (PCM model). When only isotropic carbon chemical shifts were used, we get 7.37% DP4 + probability for the correct configuration (*SRRSRSSSR*) while 25.52 and 67.12 % probabilities were obtained for wrong *SRRSRRRRS* and *SRRSRRSRS* configurations, respectively. These probabilities do not assign the correct configuration unambiguously. By using only the isotropic proton chemical shifts, we found a 100 % DP4+ probability for the correct configuration. We also calculated the probabilities from carbon and proton chemical shifts together and 100% for the correct one was found. These results also corroborate the ^{1}H RCSA outcomes.

Supplementary Note 16

Experimental data for the investigated molecules

Supplementary Table 18. Experimental ^{1}H ΔRCSA values for 80 µg of strychnine from PMMA-d_8 (70/0.04) gel in a stretching device (4.2/3.2 mm) collected in an 800 MHz spectrometer.

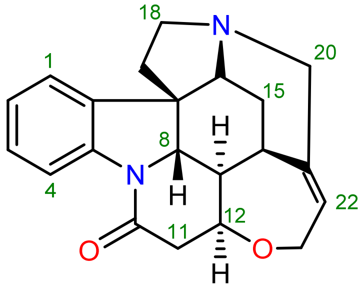

Atom	ΔRCSA, stretched gel (Hz)
H1	-2.4
H2	-2.4
H3	-2.4
H4	-3.2
H11a	-3.2
H11b	-1.6
H12	-1.6
H13	-0.8
H8	-1.6
H14	1.6
H15b	-0.8
H17b	2.4
H17a	-1.6
H16	-3.2
H18b	-0.8
H20a	-1.6
H20b	-2.4
H22	-2.4
H23a	-1.6
H23b	-3.2

Supplementary Table 19. Experimental ^{1}H ΔRCSA values for 10 μg of strychnine from PMMA-d_8 (70/0.003) gel in a micro stretching device (2.2/1.8 mm) collected in a 700 MHz spectrometer. Number of scans 1280.

Atom	ΔRCSA, stretched gel (Hz)
H4	-1.1
H3	-7.1
H1	-7.3
H2	-1.6
H23b	-1.4
H23a	1.2
H8	-2.5
H14	0.7
18a	0.1
H12	Reference

Supplementary Table 20. Experimental ^{1}H ΔRCSA values for strychnine (12 mg) measured in PPA-L-Val$_{dec}$ collected on an 800 MHz spectrometer using a normal 5 mm NMR tube.

Atom	ΔRCSA (Hz)
H1	6.4
H2	-6.4
H3	-6.4
H4	0.8
H11a	-15.2
H11b	-5.6
H12	-8.8
H13	-7.2
H8	-7.2
H14	-0.8
H15a	Reference
H15b	-10.4
H18a	-12
H18b	0.8
H20a	-9.6
H20b	-4
H22	-1.6
H23a	-1.6
H23b	3.2

Supplementary Table 21. Experimental ^{1}H ΔRCSA values for 8 mg strychnine from a PMMA (70/0.04) gel in a stretching device (4.2/3.0 mm) collected at 700 MHz spectrometer.

Atom	ΔRCSA, stretched gel (Hz)
H1	-2.4
H2	-2.4
H3	-2.4
H4	-3.2
H11a	-3.2
H11b	-1.6
H12	-1.6
H13	-0.8
H8	-1.6
H14	1.6
H15a	Reference
H15b	-0.8
H17b	2.4
H17a	-1.6
H16	-3.2
H18b	-0.8
H20a	-1.6
H20b	-2.4
H22	-2.4
H23a	-1.6
H23b	-3.2

Supplementary Table 22. Experimental ^{1}H ΔRCSA values for 3 mg estrone from Poly-HEMA (70/0.04) gel in a stretching device (4.2/3.0) collected in an 800 MHz spectrometer.

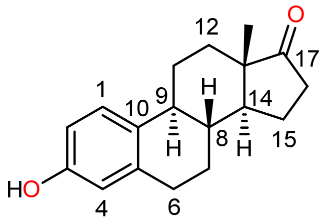

Atom	ΔRCSA, stretched gel (Hz)
H1	-4.4
H4	-5.6
H2	-4.2
H14	-2.5
H9	-2.6
H8	-3.0
H16a	-7.4
H16b	-3.6
H12b	-1.5
Me-C13	-2.6
H7b	-2.8
H7a	-2.9
H15a	-5.0
H11a	Reference

Supplementary Table 23. Experimental ^{1}H ΔRCSA values for 1 mg retrorsine from a PMMA(70/0.04) gel in a stretching device (4.2/3.0 mm) at a resonance frequency of 800 MHz. The configurations were labelled via the *R* or *S* configuration of carbons C2, C3, C11 and C12 respectively, for example *RRRS* for the correct retrorsine configuration.

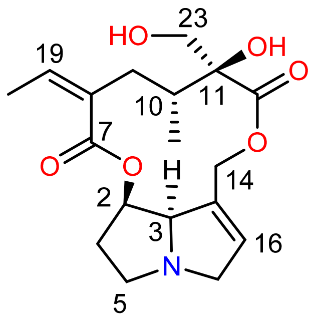

Atom	ΔRCSA, stretched gel (Hz)
H17a	0.8
H17b	-1.6
H5b	1.6
H6a	1.6
H6b	-3.2
H20	-1.6
H21	-1.6
H19	-2.4
H9a	-0.8
H9b	-1.6
H2	Reference
H14a	-0.8
H14b	-2.4
H23a	-1.6
H23b	-0.8
H10	-2.4

Supplementary Table 24. Experimental ^{1}H ΔRCSA values for 35 µg of briarane B-3 aligned in PMMA-d_8 (70/0.04) gel in a 2.2/1.8 mm Hilgenberg´s micro stretching device recorded in a spectrometer running at 800 MHz spectrometer.

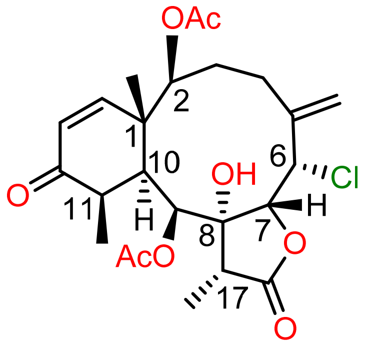

Proton Number	ΔRCSA, stretched gel (Hz)
H14	-1.9
H13	1.1
H6	0.1
H2	0.9
H10	1.7
H11	2.7
H4b	1.1
H17	0.6
Me15	0.8
Me22	0.5
Me20	0.9
Me18	1.3
Me21	-0.7
H16b	Ref.

Supplementary Table 25. Experimental ^{1}H ΔRCSA values for 40 μg of (-)-α-santonin aligned in PMMA-d_8 (70/0.009) gel in a Hilgenberg´s micro stretching device at 600 MHz spectrometer.

Proton	ΔRCSA, stretched gel (Hz)
H6	2.8
H7	Ref.
H9b	3.7
H3	0.5
Me-C9	-1.3
H4a	-2.9
H5a	0.3
Me-C5a	-0.1
Me-C3	0.2

Supplementary Table 26. Experimental ^{1}H ΔRCSA values for 45 μg of brucine aligned in PMMA-d_8 (70/0.009) gel in a Hilgenberg´s micro stretching device at 600 MHz spectrometer.

Proton	ΔRCSA, stretched gel (Hz)
H4	0.3
H1	-2.0
H22	Ref.
H12	0.5
H23b	-0.5
H23a	-1.7
MeO-C3	3.6
H16	-0.7
H20a	3.3
H18a	2.7
H11b	-0.3
H15b	0.8

Supplementary Note 17

Alignment Tensor Parameters from SVD

Output files for SVD fitting of the ^{1}H ΔRCSAs extracted from the stretching device to correct configuration of different molecules have been listed below. The coordinates of the molecules, defining the molecular frame are listed under: “Structural coordinates and CSA tensors from Gaussian DFT calculations” in the next section.

a) For 8 mg of strychnine aligned in protonated PMMA gel:

Alignment tensor information:
A'x=-6.618e-05
A'y=-5.717e-04
A'z= 6.378e-04
Saupe tensor
S'x=-9.926e-05
S'y=-8.575e-04
S'z= 9.568e-04
Alignment tensor eigenvectors
e[x]=(0.246,-0.958,-0.145)
e[y]=(0.874, 0.155, 0.460)
e[z]=(-0.418,-0.240, 0.876)

Alignment tensor in laboratory coordinates:
[-3.294e-04,2.383e-06,-4.612e-04]
[2.383e-06,-3.759e-05,-1.843e-04]
[-4.612e-04,-1.843e-04,3.670e-04]

SVD condition number is 7.824e+00
Axial component Aa = 9.568e-04
Rhombic component Ar = 5.055e-04
Field=800.00 Teslas
rhombicity R = 0.528
Asymmetry parameter etha =7.925e-01
GDO = 1.266e-03

ZY'Z'' Euler Angles (degrees)
Set 1
(-150.1,28.8,72.5)

Set 2
(29.9,-28.8,-107.5)

b) Alignment tensor information for 8 mg of strychnine aligned in protonated PMMA gel after Monte Carlo study:

<A'x> = -6.760e-05 Std.Dev = 1.988e-05
<A'y> = -5.706e-04 Std.Dev = 1.654e-05
<A'z> = 6.382e-04 Std.Dev = 1.143e-05
Conformationally averaged quality factors statistic
<Q> = 0.254
StdDev(Q) = 0.006
Highest Q = 0.276
Lowest Q = 0.237

<Q(CSA)> = 0.257
StdDev(Q(CSA)) = 0.007
Highest Q(CSA) = 0.278
Lowest Q(CSA) = 0.237

c) Alignment tensor information for 80 ug of strychnine aligned in PMMA-d_8 gel (B3LYP-6-311+G(2d,p) in $CDCl_3$):

A'x= 9.032e-05
A'y= 3.426e-04
A'z=-4.329e-04
Saupe tensor
S'x= 1.355e-04
S'y= 5.139e-04
S'z=-6.493e-04
Alignment tensor eigenvectors
e[x]=(0.968,-0.111,-0.227)
e[y]=(0.121, 0.992, 0.033)
e[z]=(0.222,-0.060, 0.973)

Alignment tensor in laboratory coordinates:
[6.829e-05,3.727e-05,-1.119e-04]
[3.727e-05,3.367e-04,3.884e-05]
[-1.119e-04,3.884e-05,-4.050e-04]

SVD condition number is 7.898e+00
Axial component Aa = -6.493e-04

Rhombic component Ar = -2.523e-04
Field=800.00 Tesla (Although it is 800 MHz, the software gives this unit).
rhombicity R = 0.388
Asymmetry parameter etha =5.827e-01
GDO = 8.110e-04

ZY'Z'' Euler Angles (degrees)
Set 1
(-15.1,13.3,8.4)
Set 2
(164.9,-13.3,-171.6)

d) Alignment tensor information for 80 ug of strychnine aligned in PMMA-d_8 gel (PBE0-cc-pVTZ-COSMO in $CDCl_3$):

A'x= 8.778e-05
A'y= 3.219e-04
A'z=-4.097e-04
Saupe tensor
S'x= 1.317e-04
S'y= 4.829e-04
S'z=-6.146e-04
Alignment tensor eigenvectors
e[x]=(0.972,-0.094,-0.215)
e[y]=(0.106, 0.993, 0.046)
e[z]=(0.209,-0.067, 0.976)

Alignment tensor in laboratory coordinates:
[6.865e-05,3.160e-05,-1.004e-04]
[3.160e-05,3.166e-04,4.324e-05]
[-1.004e-04,4.324e-05,-3.852e-04]

SVD condition number is 7.731e+00
Axial component Aa = -6.146e-04
Rhombic component Ar = -2.342e-04
Field=800.00 Tesla
rhombicity R = 0.381
Asymmetry parameter etha =5.715e-01
GDO = 7.654e-04

ZY'Z" Euler Angles (degrees)
Set 1
(-17.8,12.7,12.0)
Set 2
(162.2,-12.7,-168.0)

e) Alignment tensor information for 80 ug of strychnine aligned in PMMA-d_8 gel (B3LYP-6-311+G(2d,p) in $CDCl_3$), using Monte Carlo analysis

<A'x> = 8.979e-05 Std.Dev = 1.210e-05
<A'y> = 3.429e-04 Std.Dev = 1.844e-05
<A'z> = -4.327e-04 Std.Dev = 1.143e-05
Conformationally averaged quality factors statistic
<Q> = 0.208
StdDev(Q) = 0.015
Highest Q = 0.260
Lowest Q = 0.167

<Q(CSA)> = 0.231
StdDev(Q(CSA)) = 0.022
Highest Q(CSA) = 0.302
Lowest Q(CSA) = 0.170

f) Alignment tensor information for 80 ug of strychnine aligned in deuterated PMMA gel (PBE0-cc-pvtz-cosmo in $CDCl_3$), using Monte Carlo analysis

<A'x> = 8.727e-05 Std.Dev = 1.161e-05
<A'y> = 3.224e-04 Std.Dev = 1.751e-05
<A'z> = -4.096e-04 Std.Dev = 1.071e-05
Conformationally averaged quality factors statistic
<Q> = 0.213
StdDev(Q) = 0.015
Highest Q = 0.263
Lowest Q = 0.175

<Q(CSA)> = 0.223
StdDev(Q(CSA)) = 0.020
Highest Q(CSA) = 0.286
Lowest Q(CSA) = 0.170

g) Alignment tensor information for 10 ug of strychnine aligned in PMMA-d_8 gel:

A'x=-6.256e-04

A'y=-8.762e-04
A'z= 1.502e-03
Saupe tensor
S'x=-9.384e-04
S'y=-1.314e-03
S'z= 2.253e-03
Alignment tensor eigenvectors
e[x]=(-0.237,-0.851, 0.469)
e[y]=(0.881, 0.015, 0.473)
e[z]=(-0.409, 0.525, 0.746)

Alignment tensor in laboratory coordinates:
[-4.636e-04,-4.610e-04,-7.539e-04]
[-4.610e-04,-3.826e-05,8.320e-04]
[-7.539e-04,8.320e-04,5.019e-04]

SVD condition number is 7.126e+00
Axial component Aa = 2.253e-03
Rhombic component Ar = 2.505e-04
Field=800.00 Tesla
rhombicity R = 0.111
Asymmetry parameter etha =1.668e-01
GDO = 2.619e-03

ZY'Z" Euler Angles (degrees)
Set 1
(127.9,41.8,134.8)
Set 2
(-52.1,-41.8,-45.2)

h) Alignment tensor information for 10 μg of strychnine aligned in PMMA-d_8 gel, using Monte Carlo analysis.

Averaged Cornilescu Quality factor: 0.425412
Alignment tensor information:
<A'x> = -6.165e-04 Std.Dev = 4.835e-05
<A'y> = -8.876e-04 Std.Dev = 6.473e-05
<A'z> = 1.504e-03 Std.Dev = 9.219e-05
Conformationally averaged quality factors statistic
<Q> = 0.425
StdDev(Q) = 0.035

Highest Q = 0.516
Lowest Q = 0.310

<Q(CSA)> = 0.455
StdDev(Q(CSA)) = 0.056
Highest Q(CSA) = 0.651
Lowest Q(CSA) = 0.296

i) Alignment tensor information for 12 mg of strychnine aligned in L-valine derived polyacetylene.

A'x=-7.675e-04
A'y=-1.406e-03
A'z= 2.173e-03
Saupe tensor
S'x=-1.151e-03
S'y=-2.109e-03
S'z= 3.260e-03
Alignment tensor eigenvectors
e[x]=(0.595,-0.308, 0.743)
e[y]=(0.775,-0.028,-0.632)
e[z]=(0.215, 0.951, 0.221)

Alignment tensor in laboratory coordinates:
[-1.014e-03,6.164e-04,4.526e-04]
[6.164e-04,1.892e-03,6.079e-04]
[4.526e-04,6.079e-04,-8.780e-04]

SVD condition number is 8.824e+00
Axial component Aa = 3.260e-03
Rhombic component Ar = 6.383e-04
Field=800.00 Tesla
rhombicity R = 0.196
Asymmetry parameter etha =2.937e-01
GDO = 3.845e-03

ZY'Z" Euler Angles (degrees)
Set 1
(77.2,77.2,-139.6)
Set 2

(-102.8,-77.2,40.4)

j) Alignment tensor information for 12 mg of strychnine aligned in L-valine derived polyacetylene, using Monte Carlo analysis.

Averaged Cornilescu Quality factor: 0.348089
Alignment tensor information:
<A'x> = -7.663e-04 Std.Dev = 6.329e-05
<A'y> = -1.404e-03 Std.Dev = 6.362e-05
<A'z> = 2.171e-03 Std.Dev = 7.755e-05
Conformationally averaged quality factors statistic
<Q> = 0.348
StdDev(Q) = 0.022
Highest Q = 0.411
Lowest Q = 0.294

<Q(CSA)> = 0.441
StdDev(Q(CSA)) = 0.030
Highest Q(CSA) = 0.537
Lowest Q(CSA) = 0.344

k) For 3 mg of estrone is aligned in poly-HEMA /DMSO-d_6 gel:

Alignment tensor:
A'x=-1.532e-04,
A'y=-6.171e-04 and
A'z= 7.703e-04
Saupe tensor:
S'x=-2.299e-04,
S'y=-9.256e-04 and
S'z= 1.156e-03
Alignment tensor eigenvectors:
e[x]=(0.585, 0.074,-0.808)
e[y]=(0.178,-0.983, 0.038)
e[z]=(0.791, 0.166, 0.588)
Alignment tensor in laboratory coordinates:
[4.106e-04,2.021e-04,4.269e-04]
[2.021e-04,-5.764e-04,1.075e-04]
[4.269e-04,1.075e-04,1.658e-04]
SVD condition number is 6.339e+00

Axial component: Aa = 1.156e-03
Rhombic component: Ar = 4.638e-04
Rhombicity:R = 0.401
Asymmetry parameter: etha =6.021e-01
GDO = 1.450e-03
ZY'Z" Euler Angles (degrees)
Set 1
(11.8,54.0,2.7)
Set 2
(-168.2,-54.0,-177.3)

l) For 3 mg of estrone is aligned in poly-HEMA /DMSO-d_6 gel, using Monte Carlo analysis
Alignment tensor information:
<A'x> = 1.158e-04 Std.Dev = 2.367e-05
<A'y> = 6.415e-04 Std.Dev = 2.443e-05
<A'z> = -7.573e-04 Std.Dev = 2.651e-05
Quality factors statistic
<Q> = 0.211
StdDev(Q) = 0.017
Highest Q = 0.253
Lowest Q = 0.155

m) For 2 mg of retrorsine is aligned in PMMA gel:

Alignment tensor:
A'x=-1.318e-04,
A'y=-3.986e-04 and
A'z= 5.304e-04
Saupe tensor:
S'x=-1.977e-04,
S'y=-5.979e-04 and
S'z= 7.956e-04
Alignment tensor eigenvectors:
e[x]=(0.646,-0.075, 0.759)
e[y]=(0.762, 0.117,-0.637)
e[z]=(-0.042, 0.990, 0.133)
Alignment tensor in laboratory coordinates:
[-2.855e-04,-5.119e-05,1.258e-04]
[-5.119e-05,5.139e-04,1.069e-04]
[1.258e-04,1.069e-04,-2.284e-04]
SVD condition number is 5.461e+00

Axial component:
Aa = 7.956e-04
Rhombic component:
Ar = 2.668e-04
Rhombicity:
R = 0.335
Asymmetry parameter:
etha =5.029e-01
GDO = 9.750e-04
ZY'Z" Euler Angles (degrees)
Set 1
(92.4,82.4,-140.0)
Set 2
(-87.6,-82.4,40.0)

n) For 2 mg of retrorsine is aligned in PMMA gel using Monte Carlo analysis
Averaged Cornilescu Quality factor: 0.383206
Alignment tensor information:
<A'x> = -1.527e-04 Std.Dev = 2.104e-05
<A'y> = -4.213e-04 Std.Dev = 1.967e-05
<A'z> = 5.741e-04 Std.Dev = 2.159e-05
Conformationally averaged quality factors statistic
<Q> = 0.383
StdDev(Q) = 0.018
Highest Q = 0.440
Lowest Q = 0.330
<Q(CSA)> = 0.343
StdDev(Q(CSA)) = 0.019
Highest Q(CSA) = 0.406
Lowest Q(CSA) = 0.291

o) For 35 μg of briarane B-3 aligned in PMMA-d_8 gel:
Alignment tensor information:
A'x=-6.430e-05
A'y=-5.621e-04
A'z= 6.264e-04
Saupe tensor
S'x=-9.645e-05
S'y=-8.431e-04
S'z= 9.396e-04

Alignment tensor eigenvectors
e[x]=(-0.383, 0.885, 0.266)
e[y]=(-0.581,-0.454, 0.676)
e[z]=(0.719, 0.104, 0.687)

Alignment tensor in laboratory coordinates:
[1.247e-04,-7.931e-05,5.366e-04]
[-7.931e-05,-1.593e-04,2.023e-04]
[5.366e-04,2.023e-04,3.459e-05]

SVD condition number is 5.457e+00
Axial component Aa = 9.396e-04
Rhombic component Ar = 4.978e-04
Field=800.00 Tesla
rhombicity R = 0.530
Asymmetry parameter etha =7.947e-01
GDO = 1.244e-03

ZY'Z" Euler Angles (degrees)
Set 1
(8.3,46.6,111.4)
Set 2
(-171.7,-46.6,-68.6)

p) For 35 μg of briarane B-3 aligned in PMMA-d_8 gel using Monte Carlo analysis
Alignment tensor information:
<A'x> = -5.901e-05 Std.Dev = 4.780e-05
<A'y> = -4.283e-04 Std.Dev = 3.703e-04
<A'z> = 4.873e-04 Std.Dev = 4.004e-04
Conformationally averaged quality factors statistic
<Q> = 0.176
StdDev(Q) = 0.036
Highest Q = 0.272
Lowest Q = 0.074

<Q(CSA)> = 0.219
StdDev(Q(CSA)) = 0.047
Highest Q(CSA) = 0.373
Lowest Q(CSA) = 0.083

Supplementary References

1 Gil, R. R., Gayathri, C., Tsarevsky, N. V. & Matyjaszewski, K. Stretched Poly(methyl methacrylate) Gel Aligns Small Organic Molecules in Chloroform. Stereochemical Analysis and Diastereotopic Proton NMR Assignment in Ludartin Using Residual Dipolar Couplings and 3J Coupling Constant Analysis. *J. Org. Chem.* **73**, 840-848 (2008).

2 Gayathri, C., Tsarevsky, N. V. & Gil, R. R. Residual Dipolar Couplings (RDCs) Analysis of Small Molecules Made Easy: Fast and Tuneable Alignment by Reversible Compression/Relaxation of Reusable PMMA Gels. *Chem. Eur. J.* **16**, 3622-3626 (2010).

3 Nath, N. *et al.* Determination of Relative Configuration from Residual Chemical Shift Anisotropy. *J. Am. Chem. Soc.* **138**, 9548-9556 (2016).

4 Gil-Silva, L. F., Santamaria-Fernandez, R., Navarro-Vazquez, A. & Gil, R. R. Collection of NMR Scalar and Residual Dipolar Couplings Using a Single Experiment. *Chem. Eur. J.* **22**, 472-476 (2016).

5 Meyer, N. C., Krupp, A., Schmidts, V., Thiele, C. M. & Reggelin, M. Polyacetylenes as Enantiodifferentiating Alignment Media. *Angew. Chem. Int. Edit.* **51**, 8334-8338 (2012).

6 Lesot, P. *et al.* H-2 and C-13 NMR-Based Enantiodetection Using Polyacetylene versus Polypeptide Aligning Media: Versatile and Complementary Tools for Chemists. *Chempluschem* **84**, 144-153 (2019).

7 https://www.youtube.com/watch?v=C8cNWgJViGw

8 Nath, N., Fuentes, J. C., Reggelin, M. & Griesinger, C. Uniequivocal determination of 3D molecular structure using proton residual chemical shift anisotropy. *SMASH Conference,* Philadelphia, USA (2018).

9 Liu, Y. Z. & Prestegard, J. H. A device for the measurement of residual chemical shift anisotropy and residual dipolar coupling in soluble and membrane-associated proteins. *J. Biomol. NMR* **47**, 249-258 (2010).

10 Garcia, M. E., Woodruff, S. R., Hellemann, E., Tsarevsky, N. V. & Gil, R. R. Di(ethylene glycol) methyl ether methacrylate (DEGMEMA)-derived gels align small organic molecules in methanol. *Magn. Reson. Chem.* **55**, 206-209 (2017).

11 Wolinski, K., Hinton, J. F. & Pulay, P. Efficient implementation of the gauge independent atomic orbital method for NMR chemical shift calculations. *J. Am. Chem. Soc.* **112**, 8251-8260 (1990).

12 Tomasi, J., Mennucci, B. & Cammi, R. Quantum Mechanical Continuum Solvation Models. *Chem. Rev.* **105**, 2999-3094 (2005).

13 Gaussian 09, Revision A.02, Frisch, M. J. et al. Gaussian, Inc., Wallingford CT, 2016.

14 Halgren, T. A. Merck Molecular Force Field. I. Basis, Form, Scope, Parameterization, and Performance of MMFF94. *J. Comput. Chem.* **17**, 490-519 (1996).

15 Pescitelli, G. & Bruhn, T. Good Computational Practice in the Assignment of Absolute Configurations by TDDFT Calculations of ECD Spectra. *Chirality* **28**, 466-476 (2016).

16 Keith, T. A. & Bader, R. F. W. Calculation of magnetic response properties using a continuous set of gauge transformations. *Chem. Phys. Lett.* **210**, 223-231(1993).

17 Tirado-Rives, J. & Jorgensen, W. L. Performance of B3LYP Density Functional Methods for a Large Set of Organic Molecules. *J. Chem. Theory Comput.* **4**, 297-306 (2008).

18 Adamo, C. Toward reliable density functional methods without adjustable parameters: The PBE0 model *J. Chem. Phys.* **110**, 6158-6170 (1999).

19 H., D. J. T. Gaussian basis sets for use in correlated molecular calculations. I. The atoms boron through neon and hydrogen *J. Chem. Phys.* **90**, 1007-1023 (1989).

20 Klamt, A. & Schüürmann, G. COSMO: a new approach to dielectric screening in solvents with explicit expressions for the screening energy and its gradient *J. Chem. Soc., Perkin Trans.* ***2,*** 799-805 (1993).

21 Toomsalu, E. & Burk, P. Critical test of some computational methods for prediction of NMR ^{1}H and ^{13}C chemical shifts. *J. Mol. Model.* **9**, 244-265 (2015).

22 Willoughby, P. H., Jansma, M. J. & Hoye, T. R. A guide to small-molecule structure assignment through computation of (^{1}H and ^{13}C) NMR chemical shifts. *Nat Protoc.* **9**, 643-660 (2017).

23 Iron, M. A. Evaluation of the Factors Impacting the Accuracy of 13C NMR Chemical Shift Predictions using Density Functional Theory—The Advantage of Long-Range Corrected Functionals. *J. Chem. Theory Comput.* **13**, 5798-5819 (2017).

24 Hallwass, F. *et al.* Residual Chemical Shift Anisotropy (RCSA): A Tool for the Analysis of the Configuration of Small Molecules. *Angew. Chem. Int. Edit.* **50**, 9487-9490 (2011).

25 Grimblat, N., Zanardi, M. M. & Sarotti, A. M. Beyond DP4: an Improved Probability for the Stereochemical Assignment of Isomeric Compounds using Quantum Chemical Calculations of NMR Shifts. *J. Org. Chem.* **80**, 12526-12534 (2015).

26. Aguilar, J. A., Nilsson, M., Bodenhausen, G., Morris, G. A. Spin Echo NMR Spectra without J Modulation. *Chem. Commun.* **48**, 811-813 (2012)
