# Supplementary material for: Relative configuration of micrograms of natural compounds using proton residual chemical shift anisotropy
Source: Nat Commun. 2020 Sep 1;11:4372. doi: 10.1038/s41467-020-18093-5 (PMC7463026; doi:10.1038/s41467-020-18093-5)
Supplement: Supplementary file 2 — Description of Additional Supplementary Files [file 41467_2020_18093_MOESM2_ESM.pdf]

Description of Additional Supplementary Files

File Name: Supplementary Data 1

Description: Structural coordinates and CSA tensors from Gaussian DFT calculations
