# Supplementary material for: Relative configuration of micrograms of natural compounds using proton residual chemical shift anisotropy
Source: Nat Commun. 2020 Sep 1;11:4372. doi: 10.1038/s41467-020-18093-5 (PMC7463026; doi:10.1038/s41467-020-18093-5)
Supplement: Supplementary file 3 — Supplementary Data 1 [file 41467_2020_18093_MOESM3_ESM.zip › 225386_2_data_set_4790222_qdyrnz (3).docx]

**Supplementary Data 1**

**Structural coordinates and CSA tensors from Gaussian DFT calculations**

It may be noted that the numbering given in the molecular structures of all molecules and the DFT numbering generated inside Gaussian are different. The three-dimensional structures of all molecules with DFT positions (i.e. C1, C2 etc.) are listed just above the Cartesian co-ordinates. The four columns of the structural co-ordinates represent the atom’s type, x, y, z Cartesian c-ordinates, respectively. The chemical shielding tensor, which is represented by

$$\left( \begin{matrix} \sigma_{xx} & \sigma_{xy} & \sigma_{xz} \\ \sigma_{yx} & \sigma_{yy} & \sigma_{yz} \\ \sigma_{zx} & \sigma_{zy} & \sigma_{zz} \end{matrix} \right)$$

in the matrix form, is reported by the values of the corresponding matrix elements in the molecular frame that results from the DFT geometry optimization. In case of ensembles, all structures were superimposed on the first member of the ensemble. The Eigenvalues represent$\sigma_{11}, \sigma_{22}$, and $\sigma_{33}$, respectively. The isotropic chemical shift is given by ($\sigma_{11}+\sigma_{22}+\sigma_{33})/3)$ and the anisotropy of the tensor is given by ($\sigma_{33}-(\sigma_{11}+\sigma_{22})/2)$.

a) Strychnine

**
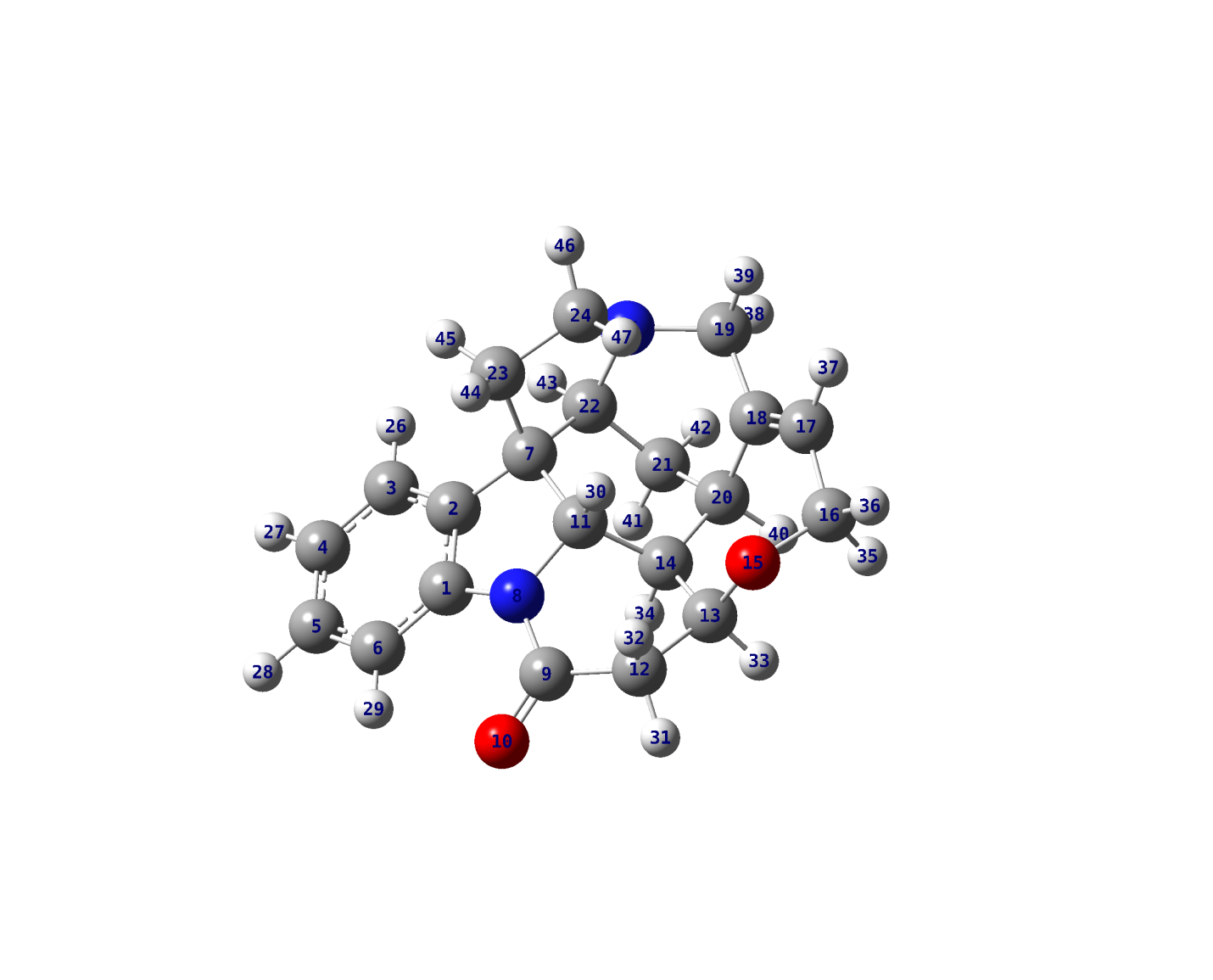
**

C 2.35889 0.43918 0.17206

C 2.01301 -0.91285 0.02306

C 2.98909 -1.84477 -0.31594

C 4.30706 -1.41665 -0.51194

C 4.63594 -0.06862 -0.35294

C 3.67086 0.88229 -0.00494

C 0.55303 -1.12798 0.36506

N 1.20883 1.19808 0.48806

C 1.08171 2.56307 0.31406

O 2.03764 3.31715 0.19406

C 0.0009 0.33597 0.41506

C -0.35934 3.07494 0.31706

C -1.42026 2.20385 -0.41194

C -0.81414 0.8359 -0.79294

O -2.52326 2.14175 0.48806

C -3.7142 1.53565 -0.02294

C -3.71707 0.03465 0.13906

C -2.821 -0.76728 -0.44794

C -2.72087 -2.25127 -0.15494

C -1.73905 -0.24318 -1.39294

C -0.81994 -1.4051 -1.81694

C -0.26889 -2.07905 -0.55794

C 0.36609 -1.839 1.73006

C -1.09887 -2.28112 1.70306

N -1.36784 -2.61915 0.29306

H 2.73419 -2.89579 -0.42594

H 5.07512 -2.13658 -0.78094

H 5.66291 0.25547 -0.50194

H 3.91376 1.92832 0.11406

H -0.60111 0.47892 1.31906

H -0.32743 4.09294 -0.07694

H -0.68734 3.14791 1.36206

H -1.74931 2.70682 -1.33594

H -0.08616 1.06096 -1.58694

H -3.85323 1.82663 -1.07794

H -4.53424 1.98157 0.54906

H -4.45903 -0.39342 0.81206

H -2.97382 -2.84329 -1.04494

H -3.43284 -2.54633 0.62306

H -2.22308 0.18678 -2.28394

H 0.01002 -1.03403 -2.42894

H -1.35788 -2.13715 -2.42994

H 0.38919 -2.90899 -0.85394

H 0.60604 -1.19198 2.58006

H 1.03217 -2.70794 1.77406

H -1.28679 -3.14414 2.35406

H -1.75094 -1.46218 2.05006

SCF GIAO Magnetic shielding tensor (ppm):

1 C Isotropic = 36.8907 Anisotropy = 128.3778

XX= -0.8024 YX= 41.5079 ZX= 29.0385

XY= 52.6198 YY= -0.3739 ZY= -27.0483

XZ= 40.7251 YZ= -17.4130 ZZ= 111.8485

Eigenvalues: -57.3448 45.5410 122.4759

2 C Isotropic = 46.3824 Anisotropy = 150.5542

XX= -14.9399 YX= -17.8670 ZX= 33.0778

XY= -24.6017 YY= 15.8356 ZY= -17.6773

XZ= 26.1237 YZ= -14.2874 ZZ= 138.2516

Eigenvalues: -28.1694 20.5647 146.7519

3 C Isotropic = 59.0145 Anisotropy = 179.6285

XX= 42.1097 YX= -25.3512 ZX= 24.8971

XY= -24.8889 YY= -34.7783 ZY= -28.6860

XZ= 24.8244 YZ= -23.7163 ZZ= 169.7122

Eigenvalues: -43.8562 42.1329 178.7669

4 C Isotropic = 58.6576 Anisotropy = 176.7294

XX= 6.7961 YX= 38.5192 ZX= 40.2977

XY= 41.7532 YY= 7.2965 ZY= -35.9926

XZ= 43.1329 YZ= -36.4024 ZZ= 161.8802

Eigenvalues: -47.5966 47.0921 176.4772

5 C Isotropic = 54.0198 Anisotropy = 182.8481

XX= -38.3415 YX= -29.9716 ZX= 37.8664

XY= -31.6207 YY= 34.4690 ZY= -17.7109

XZ= 31.3170 YZ= -21.4643 ZZ= 165.9318

Eigenvalues: -52.7854 38.9262 175.9185

6 C Isotropic = 65.5218 Anisotropy = 165.1604

XX= 39.4696 YX= -17.7109 ZX= 24.7319

XY= -20.5048 YY= -11.3022 ZY= -28.4536

XZ= 9.5720 YZ= -27.8602 ZZ= 168.3981

Eigenvalues: -20.2162 41.1530 175.6287

7 C Isotropic = 128.9863 Anisotropy = 26.8846

XX= 140.0167 YX= 7.8632 ZX= -2.3431

XY= 15.0907 YY= 123.4281 ZY= 0.7757

XZ= -7.0473 YZ= -3.9315 ZZ= 123.5142

Eigenvalues: 117.4593 122.5903 146.9094

8 N Isotropic = 78.8722 Anisotropy = 91.4552

XX= 0.4330 YX= -41.2337 ZX= 11.5469

XY= -82.5325 YY= 111.9284 ZY= 6.6640

XZ= 13.6659 YZ= 9.5956 ZZ= 124.2553

Eigenvalues: -28.5481 125.3225 139.8424

9 C Isotropic = 11.0629 Anisotropy = 113.4971

XX= -13.9128 YX= 32.9735 ZX= -2.2629

XY= 54.0170 YY= -38.7285 ZY= 12.9327

XZ= -2.6379 YZ= 8.0929 ZZ= 85.8300

Eigenvalues: -72.1682 18.6293 86.7276

10 O Isotropic = -87.2934 Anisotropy = 607.3544

XX= -256.0735 YX= -88.2330 ZX= 22.0562

XY= -103.3468 YY= -318.0241 ZY= 88.9190

XZ= 7.3398 YZ= 27.4863 ZZ= 312.2173

Eigenvalues: -392.1440 -187.3458 317.6095

11 C Isotropic = 122.3029 Anisotropy = 39.6743

XX= 126.1170 YX= 14.3865 ZX= -0.5456

XY= 8.1548 YY= 142.3799 ZY= 3.4134

XZ= 12.9080 YZ= 2.5974 ZZ= 98.4117

Eigenvalues: 97.0866 121.0696 148.7524

12 C Isotropic = 140.1649 Anisotropy = 39.3993

XX= 166.2259 YX= 0.7523 ZX= 2.7813

XY= 4.4963 YY= 132.1914 ZY= 8.2053

XZ= -2.8008 YZ= 2.9697 ZZ= 122.0774

Eigenvalues: 119.5734 134.4903 166.4311

13 C Isotropic = 105.4469 Anisotropy = 54.7342

XX= 121.8778 YX= 6.6650 ZX= -27.8431

XY= -0.1677 YY= 89.3702 ZY= -9.0433

XZ= -25.5184 YZ= 0.8944 ZZ= 105.0927

Eigenvalues: 85.0373 89.3670 141.9364

14 C Isotropic = 135.3807 Anisotropy = 18.9412

XX= 139.3441 YX= 17.1120 ZX= 6.3343

XY= 4.1128 YY= 128.4622 ZY= -7.4225

XZ= -13.1934 YZ= 1.0678 ZZ= 138.3356

Eigenvalues: 121.9224 136.2114 148.0081

15 O Isotropic = 258.8915 Anisotropy = 97.5184

XX= 275.5265 YX= 32.0349 ZX= 43.9535

XY= 22.6132 YY= 244.2736 ZY= 12.2092

XZ= 52.3761 YZ= 3.3719 ZZ= 256.8745

Eigenvalues: 212.0871 240.6837 323.9038

16 C Isotropic = 119.4988 Anisotropy = 71.9380

XX= 150.0530 YX= 20.0732 ZX= 19.1379

XY= 23.1401 YY= 110.0487 ZY= 8.2135

XZ= 26.1257 YZ= 5.2279 ZZ= 98.3948

Eigenvalues: 89.7479 101.2911 167.4575

17 C Isotropic = 52.6240 Anisotropy = 132.9887

XX= 73.0678 YX= -45.0597 ZX= 66.1691

XY= -43.7114 YY= 13.3636 ZY= 52.4965

XZ= 71.8777 YZ= 36.5648 ZZ= 71.4406

Eigenvalues: -54.7821 71.3710 141.2831

18 C Isotropic = 33.1360 Anisotropy = 170.2952

XX= 65.6820 YX= -28.0373 ZX= 77.9630

XY= -16.2819 YY= -42.0358 ZY= 51.2857

XZ= 69.5826 YZ= 41.0825 ZZ= 75.7619

Eigenvalues: -75.2346 27.9765 146.6661

19 C Isotropic = 131.2794 Anisotropy = 28.0306

XX= 144.3913 YX= -1.8338 ZX= 12.0175

XY= -10.7759 YY= 127.9763 ZY= -4.9693

XZ= 6.2314 YZ= -2.7868 ZZ= 121.4708

Eigenvalues: 117.9784 125.8934 149.9665

20 C Isotropic = 150.4183 Anisotropy = 11.4807

XX= 148.3766 YX= 9.7340 ZX= 1.0879

XY= 8.7439 YY= 145.0551 ZY= -1.1592

XZ= -4.1944 YZ= 4.0832 ZZ= 157.8231

Eigenvalues: 137.1122 156.0706 158.0720

21 C Isotropic = 156.2724 Anisotropy = 13.4414

XX= 158.8389 YX= -2.8197 ZX= -9.6969

XY= 1.9286 YY= 164.4446 ZY= 3.5909

XZ= -3.5472 YZ= 1.7247 ZZ= 145.5337

Eigenvalues: 142.5596 161.0243 165.2333

22 C Isotropic = 121.2675 Anisotropy = 39.8114

XX= 131.1363 YX= 20.1341 ZX= -15.4058

XY= 20.2383 YY= 110.8506 ZY= 6.5585

XZ= -12.3470 YZ= 0.1525 ZZ= 121.8156

Eigenvalues: 94.3558 121.6383 147.8084

23 C Isotropic = 140.6153 Anisotropy = 43.2979

XX= 138.6581 YX= 13.2125 ZX= -4.9167

XY= 2.3523 YY= 130.3641 ZY= -19.1179

XZ= -13.6604 YZ= -21.5714 ZZ= 152.8236

Eigenvalues: 118.1311 134.2342 169.4805

24 C Isotropic = 134.4511 Anisotropy = 57.5836

XX= 130.1489 YX= 20.2361 ZX= 9.3338

XY= 12.3844 YY= 106.1520 ZY= 9.6754

XZ= 12.4098 YZ= 9.0378 ZZ= 167.0525

Eigenvalues: 97.7188 132.7944 172.8402

25 N Isotropic = 204.0077 Anisotropy = 62.5269

XX= 234.0940 YX= 25.8708 ZX= -2.2039

XY= 42.0248 YY= 146.2449 ZY= -14.5063

XZ= 10.6042 YZ= -12.1277 ZZ= 231.6843

Eigenvalues: 132.6868 233.6440 245.6923

26 H Isotropic = 24.1408 Anisotropy = 10.2606

XX= 29.7256 YX= -2.9378 ZX= -1.9953

XY= -2.1749 YY= 22.6988 ZY= 0.9242

XZ= -1.8755 YZ= 1.1187 ZZ= 19.9980

Eigenvalues: 19.5176 21.9235 30.9812

27 H Isotropic = 24.3432 Anisotropy = 4.9450

XX= 26.9317 YX= 1.1330 ZX= -0.9128

XY= 1.3311 YY= 25.2412 ZY= 0.4520

XZ= -0.9178 YZ= 0.3421 ZZ= 20.8567

Eigenvalues: 20.6471 24.7427 27.6398

28 H Isotropic = 24.1179 Anisotropy = 4.3674

XX= 25.2575 YX= -0.7716 ZX= -0.9113

XY= -0.8227 YY= 26.2555 ZY= 1.0559

XZ= -1.3535 YZ= 0.9388 ZZ= 20.8406

Eigenvalues: 20.4579 24.8663 27.0295

29 H Isotropic = 23.2041 Anisotropy = 9.2160

XX= 29.0221 YX= -1.0319 ZX= -1.3675

XY= -1.3649 YY= 23.6743 ZY= 0.6663

XZ= -0.1187 YZ= 1.2519 ZZ= 16.9160

Eigenvalues: 16.7554 23.5088 29.3481

30 H Isotropic = 27.7524 Anisotropy = 3.3801

XX= 27.2145 YX= -1.3119 ZX= -0.6958

XY= -0.6675 YY= 29.6433 ZY= -0.0807

XZ= -1.4226 YZ= -0.2842 ZZ= 26.3995

Eigenvalues: 25.5422 27.7092 30.0058

31 H Isotropic = 28.6446 Anisotropy = 7.9332

XX= 28.2962 YX= 0.7834 ZX= 1.1323

XY= 2.4059 YY= 33.3573 ZY= -1.1908

XZ= 1.3087 YZ= -1.6661 ZZ= 24.2803

Eigenvalues: 23.5953 28.4051 33.9334

32 H Isotropic = 29.0611 Anisotropy = 5.0738

XX= 28.9041 YX= 0.8175 ZX= -0.8395

XY= 0.1437 YY= 29.1650 ZY= 3.4227

XZ= -1.0894 YZ= 3.1174 ZZ= 29.1143

Eigenvalues: 25.5553 29.1844 32.4436

33 H Isotropic = 27.4334 Anisotropy = 6.5862

XX= 25.8979 YX= 0.3821 ZX= -1.6442

XY= -1.4639 YY= 29.3271 ZY= -3.5775

XZ= 1.4686 YZ= -3.2566 ZZ= 27.0752

Eigenvalues: 24.4944 25.9815 31.8242

34 H Isotropic = 30.6659 Anisotropy = 3.2678

XX= 30.0073 YX= 0.4189 ZX= -3.0718

XY= 1.3440 YY= 30.7394 ZY= -1.2409

XZ= 0.6298 YZ= -0.7973 ZZ= 31.2510

Eigenvalues: 29.2057 29.9476 32.8445

35 H Isotropic = 27.4197 Anisotropy = 7.4688

XX= 29.6133 YX= -2.3392 ZX= 5.2908

XY= -0.5310 YY= 26.9132 ZY= 0.0660

XZ= 2.3447 YZ= -1.3499 ZZ= 25.7328

Eigenvalues: 23.3778 26.4825 32.3989

36 H Isotropic = 27.5383 Anisotropy = 9.9548

XX= 33.5732 YX= -1.7714 ZX= -3.1572

XY= -0.6422 YY= 26.5601 ZY= 1.1464

XZ= -0.8698 YZ= 0.9214 ZZ= 22.4816

Eigenvalues: 21.9794 26.4607 34.1749

37 H Isotropic = 25.5095 Anisotropy = 5.6610

XX= 27.5838 YX= -0.8705 ZX= -1.5291

XY= -2.6192 YY= 26.2888 ZY= 1.4686

XZ= -0.9389 YZ= 1.2508 ZZ= 22.6559

Eigenvalues: 22.1191 25.1259 29.2835

38 H Isotropic = 27.8856 Anisotropy = 9.7603

XX= 29.0042 YX= 3.9035 ZX= 3.9696

XY= 2.5017 YY= 28.0327 ZY= 2.8766

XZ= 2.6586 YZ= 3.2939 ZZ= 26.6197

Eigenvalues: 23.9676 25.2966 34.3924

39 H Isotropic = 29.0814 Anisotropy = 8.3395

XX= 33.5096 YX= 1.9031 ZX= -1.8093

XY= 2.1521 YY= 28.0909 ZY= -1.7461

XZ= -1.1171 YZ= -2.1142 ZZ= 25.6437

Eigenvalues: 24.5698 28.0334 34.6411

40 H Isotropic = 28.5170 Anisotropy = 8.9537

XX= 27.2562 YX= -2.3310 ZX= 3.2216

XY= -2.2765 YY= 26.9845 ZY= -0.4514

XZ= 4.6513 YZ= -2.3266 ZZ= 31.3104

Eigenvalues: 24.1612 26.9037 34.4862

41 H Isotropic = 30.3619 Anisotropy = 6.9240

XX= 30.2634 YX= 0.6422 ZX= -3.6095

XY= 0.0576 YY= 27.0749 ZY= -0.1653

XZ= -1.1920 YZ= 1.0193 ZZ= 33.7475

Eigenvalues: 26.9466 29.1613 34.9779

42 H Isotropic = 29.4367 Anisotropy = 10.7295

XX= 26.2626 YX= 1.5994 ZX= 1.9816

XY= 1.6445 YY= 30.1270 ZY= 4.6895

XZ= 2.3353 YZ= 4.8668 ZZ= 31.9206

Eigenvalues: 25.5331 26.1873 36.5897

43 H Isotropic = 27.6585 Anisotropy = 9.4675

XX= 29.8147 YX= -3.4558 ZX= -2.8991

XY= -2.8751 YY= 29.1354 ZY= 1.7601

XZ= -2.2657 YZ= 3.2973 ZZ= 24.0254

Eigenvalues: 22.7083 26.2970 33.9702

44 H Isotropic = 29.9158 Anisotropy = 7.2826

XX= 27.8699 YX= 0.7845 ZX= 3.1020

XY= 1.6830 YY= 27.8012 ZY= 0.3504

XZ= 0.9180 YZ= 0.6480 ZZ= 34.0762

Eigenvalues: 26.4320 28.5444 34.7708

45 H Isotropic = 29.8649 Anisotropy = 9.3393

XX= 31.0993 YX= -3.3647 ZX= 1.5502

XY= -3.3999 YY= 30.9242 ZY= -3.6326

XZ= -0.1078 YZ= -5.2280 ZZ= 27.5714

Eigenvalues: 24.2089 29.2948 36.0911

46 H Isotropic = 28.6078 Anisotropy = 11.4189

XX= 25.2522 YX= 2.2440 ZX= -0.9112

XY= 2.2138 YY= 28.9686 ZY= -4.3850

XZ= -1.8869 YZ= -6.0342 ZZ= 31.6025

Eigenvalues: 23.9181 25.6849 36.2204

47 H Isotropic = 28.7757 Anisotropy = 5.9352

XX= 28.7106 YX= -0.5746 ZX= -1.7456

XY= -1.9958 YY= 26.8714 ZY= 0.7867

XZ= -2.8488 YZ= 1.5824 ZZ= 30.7450

Eigenvalues: 26.2107 27.3839 32.7325

**Estrone**

**
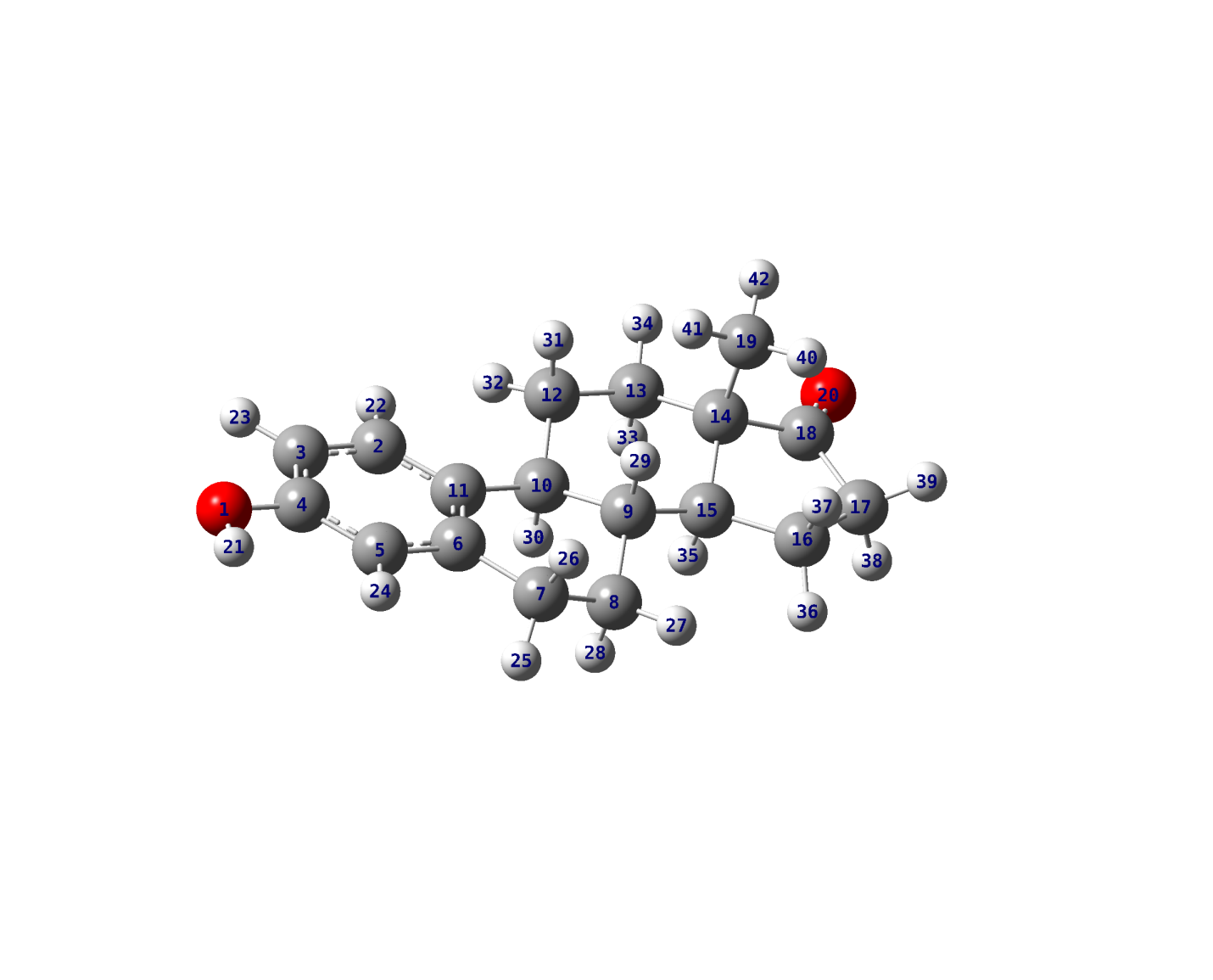
**

O 5.95328 -0.23977 0.27778

C 2.59867 -1.51728 -0.34518

C 3.97805 -1.47376 -0.16328

C 4.59042 -0.24758 0.10546

C 3.81214 0.90618 0.18223

C 2.42155 0.8608 -0.00033

C 1.64175 2.16025 0.11751

C 0.21783 2.06973 -0.4396

C -0.4634 0.79001 0.05864

C 0.27869 -0.44614 -0.52155

C 1.78762 -0.37052 -0.2645

C -0.37434 -1.77565 -0.06694

C -1.89584 -1.8331 -0.33185

C -2.60185 -0.60421 0.25237

C -1.94384 0.68144 -0.31338

C -2.94538 1.80563 0.02668

C -4.31399 1.13301 -0.24053

C -4.06077 -0.38383 -0.15948

C -2.63083 -0.67162 1.80474

O -4.88686 -1.24517 -0.38713

H 6.25581 0.6643 0.45597

H 2.14806 -2.48096 -0.56062

H 4.58437 -2.37217 -0.22947

H 4.28887 1.86586 0.38547

H 2.19521 2.96714 -0.38064

H 1.58944 2.44415 1.1803

H -0.34914 2.95924 -0.13754

H 0.24035 2.06595 -1.53976

H -0.36819 0.77201 1.15596

H 0.13536 -0.39109 -1.61596

H -0.16596 -1.93454 0.99971

H 0.08838 -2.61806 -0.59268

H -2.07634 -1.86804 -1.41549

H -2.31464 -2.75871 0.08136

H -1.97865 0.5742 -1.41305

H -2.79818 2.6988 -0.58886

H -2.85297 2.1172 1.07452

H -4.68964 1.35558 -1.24765

H -5.10808 1.42167 0.45766

H -3.12615 0.1955 2.25515

H -1.625 -0.73258 2.22977

SCF GIAO Magnetic shielding tensor (ppm):

1 O Isotropic = 213.6227 Anisotropy = 73.6521

XX= 261.0246 YX= 6.3627 ZX= 7.2010

XY= 15.6563 YY= 166.7534 ZY= -5.9002

XZ= 3.8270 YZ= -9.5887 ZZ= 213.0902

Eigenvalues: 164.0649 214.0791 262.7241

2 C Isotropic = 50.6995 Anisotropy = 177.7320

XX= 26.0789 YX= -29.1339 ZX= -23.4901

XY= -32.0642 YY= -33.5765 ZY= -33.9002

XZ= -32.5250 YZ= -34.8735 ZZ= 159.5962

Eigenvalues: -54.9900 37.9010 169.1875

3 C Isotropic = 67.2286 Anisotropy = 142.3520

XX= 36.5782 YX= 40.1188 ZX= -10.3703

XY= 33.4835 YY= 8.0434 ZY= -19.4900

XZ= -13.3177 YZ= -21.1405 ZZ= 157.0641

Eigenvalues: -17.7587 57.3144 162.1299

4 C Isotropic = 20.9682 Anisotropy = 138.3258

XX= -63.9254 YX= -7.6709 ZX= -23.8349

XY= -0.9368 YY= 18.8213 ZY= -14.2260

XZ= -20.4575 YZ= -16.9781 ZZ= 108.0088

Eigenvalues: -67.1903 16.9096 113.1854

5 C Isotropic = 65.9551 Anisotropy = 113.9366

XX= 52.8815 YX= -37.8539 ZX= -16.1969

XY= -25.5307 YY= 8.5794 ZY= -23.2802

XZ= -10.4897 YZ= -27.6073 ZZ= 136.4044

Eigenvalues: -13.4440 69.3965 141.9128

6 C Isotropic = 37.1581 Anisotropy = 189.3309

XX= -10.6418 YX= 30.2853 ZX= -16.4970

XY= 31.0282 YY= -35.7197 ZY= -24.9656

XZ= -21.7835 YZ= -19.8997 ZZ= 157.8358

Eigenvalues: -56.6154 4.7110 163.3787

7 C Isotropic = 148.8782 Anisotropy = 22.6403

XX= 160.4990 YX= -1.7109 ZX= 5.0103

XY= -12.4679 YY= 149.1811 ZY= -2.3469

XZ= -1.8733 YZ= 2.9675 ZZ= 136.9545

Eigenvalues: 136.7916 145.8713 163.9717

8 C Isotropic = 152.4575 Anisotropy = 16.7655

XX= 150.5394 YX= 10.0752 ZX= -0.2151

XY= 9.1941 YY= 155.4697 ZY= -3.8297

XZ= -2.9677 YZ= -1.0391 ZZ= 151.3633

Eigenvalues: 143.0525 150.6855 163.6345

9 C Isotropic = 139.8702 Anisotropy = 7.0339

XX= 140.5857 YX= -6.5966 ZX= 0.3400

XY= 2.5114 YY= 137.0307 ZY= -0.5540

XZ= 5.5886 YZ= 0.5727 ZZ= 141.9942

Eigenvalues: 135.7904 139.2607 144.5595

10 C Isotropic = 133.5334 Anisotropy = 10.1350

XX= 129.5828 YX= -11.4428 ZX= -5.1850

XY= -4.9616 YY= 130.9562 ZY= -0.8446

XZ= 2.6016 YZ= -2.0785 ZZ= 140.0612

Eigenvalues: 121.8321 138.4780 140.2901

11 C Isotropic = 44.6561 Anisotropy = 178.7186

XX= -39.9117 YX= 0.5513 ZX= -26.8165

XY= 0.2017 YY= 17.5558 ZY= -21.2643

XZ= -22.9883 YZ= -29.5700 ZZ= 156.3243

Eigenvalues: -43.1565 13.3230 163.8018

12 C Isotropic = 152.9718 Anisotropy = 19.2577

XX= 158.2974 YX= 8.1797 ZX= -0.3148

XY= 6.5922 YY= 155.7118 ZY= -7.7202

XZ= 1.7540 YZ= -9.0718 ZZ= 144.9062

Eigenvalues: 139.3134 153.7917 165.8102

13 C Isotropic = 147.4797 Anisotropy = 18.8802

XX= 144.3211 YX= -7.6409 ZX= -8.8812

XY= -1.9654 YY= 150.9728 ZY= 9.7719

XZ= -5.7103 YZ= 2.8395 ZZ= 147.1450

Eigenvalues: 138.2908 144.0817 160.0665

14 C Isotropic = 128.0888 Anisotropy = 21.9903

XX= 139.2645 YX= -15.0626 ZX= -6.1965

XY= -3.1392 YY= 118.9784 ZY= -0.5972

XZ= 4.2227 YZ= -4.7466 ZZ= 126.0234

Eigenvalues: 114.7729 126.7444 142.7490

15 C Isotropic = 127.6932 Anisotropy = 22.1041

XX= 142.3348 YX= 6.3274 ZX= 4.1868

XY= -5.0738 YY= 128.9297 ZY= 2.5615

XZ= -7.3095 YZ= 4.7417 ZZ= 111.8151

Eigenvalues: 110.9812 129.6691 142.4293

16 C Isotropic = 158.4264 Anisotropy = 24.2856

XX= 172.1376 YX= -9.1266 ZX= 1.1021

XY= -4.1020 YY= 156.7215 ZY= 4.0134

XZ= 3.4898 YZ= 3.1375 ZZ= 146.4202

Eigenvalues: 144.6100 156.0525 174.6168

17 C Isotropic = 142.8608 Anisotropy = 35.9249

XX= 139.7417 YX= -0.1901 ZX= 7.3072

XY= -0.2073 YY= 166.7374 ZY= 1.2830

XZ= 0.1070 YZ= 2.3366 ZZ= 122.1033

Eigenvalues: 121.2835 140.4882 166.8107

18 C Isotropic = -51.0662 Anisotropy = 173.4037

XX= -84.5407 YX= 11.8733 ZX= -41.2754

XY= 5.1796 YY= -123.1252 ZY= -2.6188

XZ= -35.9658 YZ= 0.1379 ZZ= 54.4675

Eigenvalues: -125.2396 -92.4952 64.5363

19 C Isotropic = 168.4988 Anisotropy = 25.0277

XX= 157.4975 YX= 7.7493 ZX= -3.7245

XY= 8.9291 YY= 164.7968 ZY= -6.1227

XZ= -3.5876 YZ= -2.0276 ZZ= 183.2022

Eigenvalues: 152.0210 168.2915 185.1840

20 O Isotropic = -245.7818 Anisotropy = 911.1947

XX= -490.3389 YX= -185.4415 ZX= -239.0082

XY= -185.6989 YY= -537.1132 ZY= -62.6717

XZ= -253.4254 YZ= -82.6888 ZZ= 290.1067

Eigenvalues: -748.2899 -350.7369 361.6813

21 H Isotropic = 27.8504 Anisotropy = 12.7422

XX= 30.0751 YX= 5.2510 ZX= 2.0787

XY= 2.3506 YY= 33.0821 ZY= 2.4241

XZ= 1.7959 YZ= 2.9018 ZZ= 20.3940

Eigenvalues: 19.7135 27.4925 36.3452

22 H Isotropic = 24.4762 Anisotropy = 11.2450

XX= 30.8524 YX= -3.5973 ZX= -0.3343

XY= -2.6200 YY= 23.2639 ZY= 0.5116

XZ= -0.1716 YZ= 0.1425 ZZ= 19.3122

Eigenvalues: 19.2853 22.1704 31.9728

23 H Isotropic = 25.0686 Anisotropy = 5.2882

XX= 27.9608 YX= 1.6178 ZX= 0.8827

XY= 0.7263 YY= 25.5038 ZY= 0.6022

XZ= 0.8417 YZ= 0.7792 ZZ= 21.7411

Eigenvalues: 21.5492 25.0626 28.5940

24 H Isotropic = 25.2561 Anisotropy = 8.3048

XX= 29.7665 YX= -2.4384 ZX= 0.5430

XY= -2.5840 YY= 24.5034 ZY= 0.0699

XZ= 0.4411 YZ= 0.0582 ZZ= 21.4983

Eigenvalues: 21.4495 23.5261 30.7926

25 H Isotropic = 29.0610 Anisotropy = 9.1281

XX= 32.1874 YX= 2.8402 ZX= -0.5539

XY= 3.3193 YY= 31.1093 ZY= -1.8333

XZ= -0.1922 YZ= -3.4978 ZZ= 23.8864

Eigenvalues: 22.9641 29.0726 35.1464

26 H Isotropic = 28.9793 Anisotropy = 7.3435

XX= 29.7773 YX= -0.2999 ZX= 2.1027

XY= 0.6373 YY= 27.9930 ZY= 3.9167

XZ= 0.9374 YZ= 5.8258 ZZ= 29.1676

Eigenvalues: 23.5457 29.5174 33.8750

27 H Isotropic = 29.9834 Anisotropy = 7.2941

XX= 32.2295 YX= -0.9293 ZX= -0.2115

XY= -1.4122 YY= 34.2227 ZY= 0.8743

XZ= -0.2851 YZ= 1.0277 ZZ= 23.4980

Eigenvalues: 23.4119 31.6922 34.8462

28 H Isotropic = 30.5402 Anisotropy = 6.4592

XX= 29.8578 YX= 1.6022 ZX= 0.2630

XY= 1.2180 YY= 29.0798 ZY= -3.1622

XZ= 1.0015 YZ= -3.8832 ZZ= 32.6831

Eigenvalues: 26.2692 30.5051 34.8463

29 H Isotropic = 30.3848 Anisotropy = 4.5889

XX= 31.2817 YX= 2.6987 ZX= -0.2887

XY= 2.7583 YY= 28.8796 ZY= -0.2518

XZ= -2.1186 YZ= 0.4628 ZZ= 30.9931

Eigenvalues: 26.9564 30.7540 33.4441

30 H Isotropic = 29.6560 Anisotropy = 2.3514

XX= 29.7372 YX= 0.6478 ZX= -1.7629

XY= -0.2458 YY= 28.1323 ZY= 0.5866

XZ= 1.2184 YZ= 0.4476 ZZ= 31.0984

Eigenvalues: 28.0100 29.7343 31.2236

31 H Isotropic = 30.5067 Anisotropy = 5.2833

XX= 31.3773 YX= -1.1506 ZX= 1.3450

XY= -1.7385 YY= 29.4263 ZY= -3.3576

XZ= -1.1343 YZ= -3.6333 ZZ= 30.7165

Eigenvalues: 26.2969 31.1943 34.0289

32 H Isotropic = 29.5629 Anisotropy = 10.1879

XX= 32.1976 YX= -2.4436 ZX= -1.3225

XY= -3.0229 YY= 33.3249 ZY= 3.0379

XZ= -1.4775 YZ= 3.0406 ZZ= 23.1663

Eigenvalues: 22.2842 30.0497 36.3548

33 H Isotropic = 30.5766 Anisotropy = 7.8208

XX= 29.7675 YX= 0.4181 ZX= 1.6964

XY= 0.6188 YY= 29.4244 ZY= 4.1417

XZ= 1.5890 YZ= 3.9967 ZZ= 32.5378

Eigenvalues: 26.5373 29.4020 35.7905

34 H Isotropic = 30.0621 Anisotropy = 9.0400

XX= 30.1848 YX= 2.4772 ZX= -0.7315

XY= 3.2524 YY= 34.6344 ZY= -0.3687

XZ= -1.0328 YZ= -0.4256 ZZ= 25.3672

Eigenvalues: 25.2092 28.8884 36.0888

35 H Isotropic = 30.3523 Anisotropy = 2.4004

XX= 30.1271 YX= -1.2257 ZX= 1.2722

XY= -1.8269 YY= 29.3306 ZY= 0.0208

XZ= -0.7435 YZ= -0.8812 ZZ= 31.5991

Eigenvalues: 28.1420 30.9623 31.9525

36 H Isotropic = 29.9232 Anisotropy = 8.4044

XX= 29.9550 YX= -0.8277 ZX= 0.1812

XY= -0.1230 YY= 33.8063 ZY= -3.6602

XZ= 0.2379 YZ= -4.2964 ZZ= 26.0081

Eigenvalues: 24.3368 29.9066 35.5261

37 H Isotropic = 30.2845 Anisotropy = 6.7372

XX= 29.0879 YX= -1.2351 ZX= -0.8875

XY= -0.8693 YY= 30.1920 ZY= 3.4383

XZ= -0.4490 YZ= 3.7203 ZZ= 31.5736

Eigenvalues: 27.1521 28.9255 34.7760

38 H Isotropic = 29.6874 Anisotropy = 8.7834

XX= 31.4285 YX= 0.3817 ZX= 4.2750

XY= -0.8478 YY= 27.7023 ZY= -1.9052

XZ= 4.7503 YZ= -2.1379 ZZ= 29.9314

Eigenvalues: 25.2417 28.2775 35.5430

39 H Isotropic = 29.4097 Anisotropy = 8.5882

XX= 33.9048 YX= -1.3639 ZX= -2.0494

XY= -1.9709 YY= 27.1044 ZY= 1.2608

XZ= -2.5436 YZ= 1.6670 ZZ= 27.2200

Eigenvalues: 25.6699 27.4241 35.1352

40 H Isotropic = 30.9511 Anisotropy = 8.5038

XX= 28.2072 YX= -2.1871 ZX= -4.1496

XY= -2.4227 YY= 30.9461 ZY= 0.4261

XZ= -3.5865 YZ= 2.2537 ZZ= 33.7000

Eigenvalues: 25.7696 30.4633 36.6202

41 H Isotropic = 30.7343 Anisotropy = 5.2143

XX= 32.5758 YX= 0.0378 ZX= 1.0463

XY= 0.2001 YY= 26.6050 ZY= -1.1419

XZ= 1.5861 YZ= -1.0274 ZZ= 33.0219

Eigenvalues: 26.4082 31.5842 34.2104

42 H Isotropic = 31.4932 Anisotropy = 10.8441

XX= 29.9158 YX= 3.8971 ZX= -3.0175

XY= 3.3717 YY= 32.7251 ZY= -3.2516

XZ= -3.2649 YZ= -4.3386 ZZ= 31.8388

Eigenvalues: 27.2929 28.4642 38.7226

**Retrorsine:** The co-ordinates are reported after superimposing all conformers with respect to the frame of the first conformer.

**
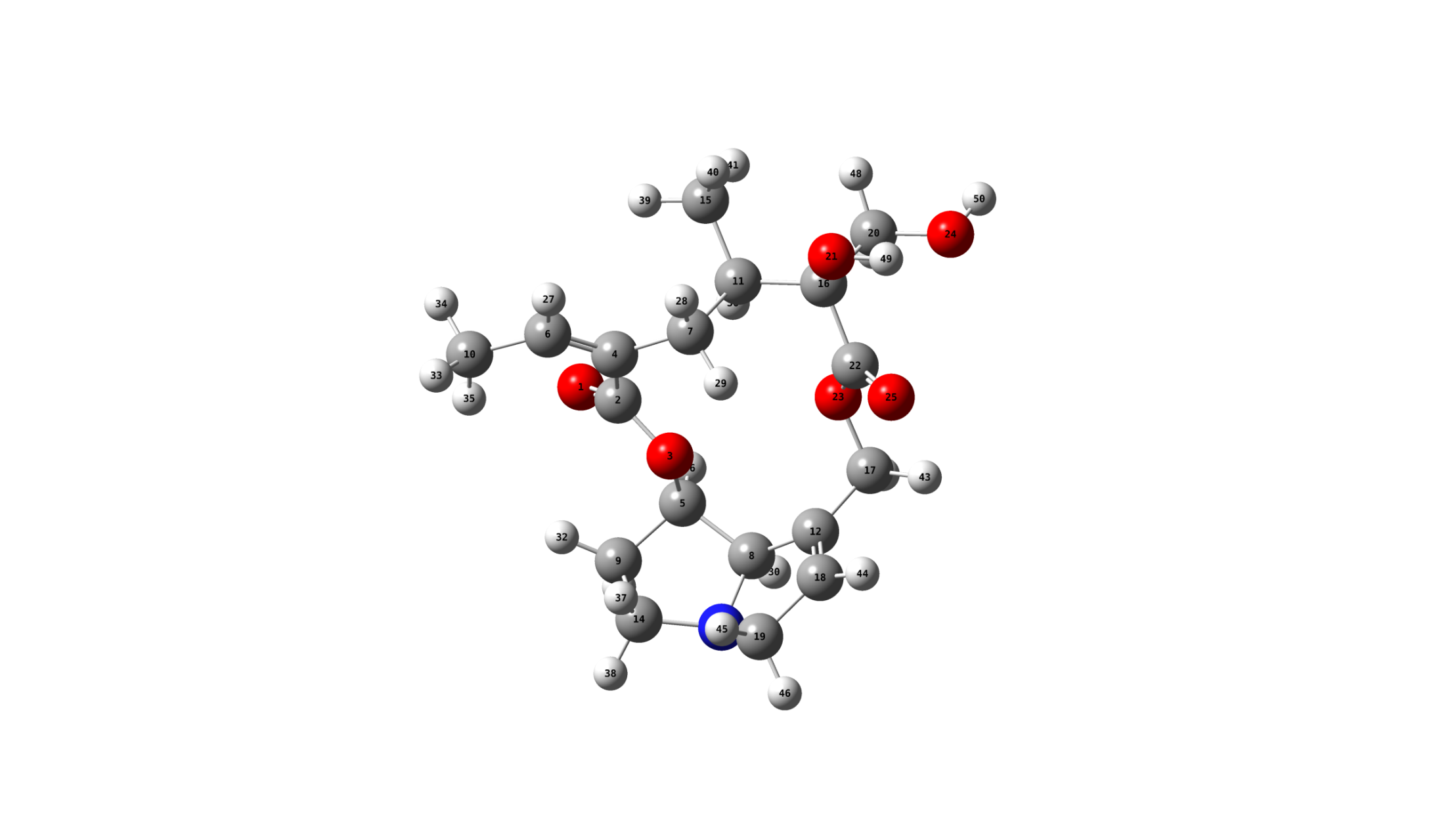
**

O -0.999293 2.257719 -1.543128

C -0.651748 1.652004 -0.542337

O -0.995164 0.383384 -0.211564

C 0.267249 2.189708 0.497493

C -1.836055 -0.302284 -1.158755

C 0.102138 3.428193 1.001541

C 1.394650 1.276421 0.927199

C -1.969113 -1.798711 -0.809841

C -3.278900 0.170393 -1.011039

C -0.980571 4.411589 0.683502

C 2.350058 0.907159 -0.241177

C -0.861971 -2.501120 -0.095088

N -3.156409 -1.912146 0.094416

C -3.735265 -0.571987 0.235725

C 3.264428 2.107207 -0.545184

C 3.142167 -0.391807 0.078840

C 0.438604 -2.831442 -0.741330

C -1.274263 -2.889971 1.117223

C -2.683214 -2.479463 1.370764

C 4.134683 -0.785809 -1.035347

O 3.904485 -0.176140 1.278746

C 2.177127 -1.590076 0.267810

O 1.302914 -1.692088 -0.777121

O 4.888161 -1.934984 -0.629440

O 2.214954 -2.349522 1.230785

H -1.446853 -0.168899 -2.174865

H 0.818570 3.781882 1.742188

H 1.973833 1.739611 1.737231

H 0.948241 0.379919 1.372745

H -2.225968 -2.352617 -1.722435

H -3.873609 -0.138847 -1.879139

H -3.380035 1.251381 -0.881016

H -1.402443 4.800786 1.615965

H -0.566054 5.253156 0.120551

H -1.806337 3.981160 0.112407

H 1.761210 0.726899 -1.149714

H -3.364200 -0.040884 1.121282

H -4.826497 -0.619532 0.300406

H 2.679838 3.015942 -0.722126

H 3.953296 2.307653 0.281832

H 3.854727 1.938078 -1.450493

H 0.262714 -3.136808 -1.779027

H 0.924696 -3.689302 -0.260089

H -0.680514 -3.446954 1.828912

H -2.715641 -1.775423 2.209435

H -3.293774 -3.350704 1.628278

H 3.626012 -1.017267 -1.976575

H 4.869326 0.005772 -1.206716

H 4.384013 -1.019334 1.420630

H 5.543984 -2.105688 -1.330841

The DFT computed chemical shift tensors for the four conformers are given below.

Conformer 1

SCF GIAO Magnetic shielding tensor (ppm):

1 O Isotropic = -76.1829 Anisotropy = 575.9985

XX= 127.4224 YX= -135.3902 ZX= -189.7778

XY= -141.4361 YY= -201.1993 ZY= 142.7586

XZ= -198.5455 YZ= 198.5260 ZZ= -154.7717

Eigenvalues: -351.9350 -184.4297 307.8161

2 C Isotropic = 23.5858 Anisotropy = 85.3262

XX= 38.9323 YX= -52.1410 ZX= -30.9658

XY= -68.5103 YY= -22.8876 ZY= -31.2536

XZ= -13.1162 YZ= -3.0072 ZZ= 54.7127

Eigenvalues: -65.4671 55.7546 80.4700

3 O Isotropic = 123.4882 Anisotropy = 145.7602

XX= 155.0428 YX= -40.0828 ZX= -73.3476

XY= 21.3528 YY= 205.5467 ZY= 61.6376

XZ= -144.9145 YZ= -52.6092 ZZ= 9.8752

Eigenvalues: -48.6066 198.4096 220.6617

4 C Isotropic = 55.4673 Anisotropy = 120.3127

XX= 22.6497 YX= 27.7549 ZX= -79.0239

XY= 27.3917 YY= 78.1225 ZY= -10.9356

XZ= -73.7577 YZ= -12.9813 ZZ= 65.6297

Eigenvalues: -37.1780 67.9041 135.6758

5 C Isotropic = 115.7275 Anisotropy = 34.5775

XX= 127.5048 YX= 3.6867 ZX= 11.4136

XY= 6.8522 YY= 118.0761 ZY= 14.5755

XZ= 14.7750 YZ= 13.2209 ZZ= 101.6016

Eigenvalues: 91.3279 117.0755 138.7791

6 C Isotropic = 56.9742 Anisotropy = 141.5187

XX= 27.8704 YX= 11.3014 ZX= -90.9424

XY= 15.2523 YY= 72.3120 ZY= -33.4783

XZ= -86.2760 YZ= -20.0478 ZZ= 70.7401

Eigenvalues: -42.2004 61.8030 151.3200

7 C Isotropic = 149.1987 Anisotropy = 20.7233

XX= 158.7112 YX= -5.8578 ZX= -2.9824

XY= -9.5509 YY= 147.8926 ZY= 4.2050

XZ= -1.6832 YZ= -2.0610 ZZ= 140.9922

Eigenvalues: 140.6888 143.8930 163.0142

8 C Isotropic = 110.2770 Anisotropy = 47.0468

XX= 131.4014 YX= -6.5755 ZX= -20.1567

XY= -13.7848 YY= 98.6031 ZY= -18.1476

XZ= -20.3998 YZ= -12.8616 ZZ= 100.8264

Eigenvalues: 75.7970 113.3924 141.6415

9 C Isotropic = 152.4887 Anisotropy = 28.5922

XX= 165.6423 YX= -11.4851 ZX= -7.3618

XY= -6.9923 YY= 151.5978 ZY= -2.1476

XZ= -13.4483 YZ= -13.4768 ZZ= 140.2260

Eigenvalues: 130.9481 154.9679 171.5502

10 C Isotropic = 173.2176 Anisotropy = 23.1518

XX= 180.9351 YX= -11.5185 ZX= -1.0840

XY= -11.2950 YY= 171.1869 ZY= -6.3241

XZ= -2.1791 YZ= -5.5886 ZZ= 167.5309

Eigenvalues: 159.3688 171.6320 188.6522

11 C Isotropic = 152.1095 Anisotropy = 23.7501

XX= 160.4761 YX= -9.6059 ZX= 2.5539

XY= -10.4218 YY= 153.8728 ZY= -1.6554

XZ= 4.9877 YZ= 3.9195 ZZ= 141.9797

Eigenvalues: 140.2410 148.1447 167.9429

12 C Isotropic = 59.8764 Anisotropy = 128.1053

XX= 3.7833 YX= 64.4707 ZX= -1.6849

XY= 79.0653 YY= 102.3526 ZY= 18.9454

XZ= -24.3424 YZ= 37.1829 ZZ= 73.4934

Eigenvalues: -39.4408 73.7901 145.2800

13 N Isotropic = 196.0102 Anisotropy = 67.2137

XX= 203.0942 YX= -43.5571 ZX= -33.8049

XY= -36.4851 YY= 179.8516 ZY= -34.9679

XZ= -39.6243 YZ= -42.4367 ZZ= 205.0849

Eigenvalues: 117.5838 229.6275 240.8194

14 C Isotropic = 134.8163 Anisotropy = 64.0560

XX= 116.6569 YX= -26.4355 ZX= 6.1364

XY= -26.3332 YY= 165.4487 ZY= -2.5663

XZ= -3.6946 YZ= -8.2017 ZZ= 122.3434

Eigenvalues: 105.0543 121.8744 177.5203

15 C Isotropic = 176.8559 Anisotropy = 23.4871

XX= 180.6640 YX= 11.4910 ZX= -4.2224

XY= 13.7427 YY= 176.3717 ZY= -2.9890

XZ= -6.6133 YZ= 1.1528 ZZ= 173.5319

Eigenvalues: 164.7383 173.3153 192.5140

16 C Isotropic = 106.8023 Anisotropy = 25.8645

XX= 110.0546 YX= 3.1092 ZX= 14.5603

XY= 1.7092 YY= 102.1455 ZY= 6.5243

XZ= 12.2902 YZ= 4.7902 ZZ= 108.2068

Eigenvalues: 94.6917 101.6698 124.0453

17 C Isotropic = 132.7849 Anisotropy = 51.4696

XX= 143.3247 YX= 25.3255 ZX= -10.7591

XY= 22.4225 YY= 143.1127 ZY= 7.8265

XZ= -7.3859 YZ= 11.8358 ZZ= 111.9173

Eigenvalues: 101.7541 129.5026 167.0980

18 C Isotropic = 55.8471 Anisotropy = 113.7067

XX= -2.0668 YX= 57.9393 ZX= -18.8813

XY= 54.3963 YY= 90.1024 ZY= 49.2609

XZ= 3.3818 YZ= 18.1847 ZZ= 79.5057

Eigenvalues: -32.9075 68.7971 131.6515

19 C Isotropic = 126.4243 Anisotropy = 59.3057

XX= 113.6458 YX= -19.8459 ZX= 18.4654

XY= -21.4966 YY= 120.0907 ZY= -14.7154

XZ= 15.5514 YZ= -19.2401 ZZ= 145.5363

Eigenvalues: 95.8494 117.4620 165.9614

20 C Isotropic = 121.0884 Anisotropy = 39.7055

XX= 116.7666 YX= -22.4411 ZX= -2.2199

XY= -19.9720 YY= 132.3666 ZY= 0.9624

XZ= 3.0856 YZ= -9.2264 ZZ= 114.1319

Eigenvalues: 101.6402 114.0662 147.5587

21 O Isotropic = 278.2872 Anisotropy = 42.1848

XX= 283.7404 YX= 22.6872 ZX= 6.2662

XY= 21.5109 YY= 282.3020 ZY= 11.4155

XZ= 8.4792 YZ= -6.7836 ZZ= 268.8192

Eigenvalues: 259.4663 268.9849 306.4104

22 C Isotropic = 13.0450 Anisotropy = 89.7072

XX= -15.6894 YX= -64.5396 ZX= -42.3146

XY= -42.2067 YY= 37.0933 ZY= -16.4040

XZ= -65.9873 YZ= -31.1280 ZZ= 17.7311

Eigenvalues: -83.0114 49.2966 72.8498

23 O Isotropic = 135.7402 Anisotropy = 157.5539

XX= 193.1295 YX= -56.7666 ZX= -24.5021

XY= -107.7293 YY= 55.8106 ZY= 39.8882

XZ= 70.6957 YZ= 150.5774 ZZ= 158.2804

Eigenvalues: -31.6178 198.0622 240.7761

24 O Isotropic = 305.6288 Anisotropy = 106.1339

XX= 312.0434 YX= -14.4145 ZX= -45.3493

XY= -25.9817 YY= 290.9844 ZY= 9.6856

XZ= -62.6705 YZ= 30.1321 ZZ= 313.8586

Eigenvalues: 258.9255 281.5762 376.3847

25 O Isotropic = -80.2892 Anisotropy = 616.5417

XX= 3.4021 YX= -190.1142 ZX= -215.0946

XY= -184.0522 YY= -25.3403 ZY= 171.6061

XZ= -233.5188 YZ= 200.1881 ZZ= -218.9293

Eigenvalues: -374.0403 -197.5659 330.7386

26 H Isotropic = 26.7004 Anisotropy = 3.1344

XX= 26.1233 YX= 0.9765 ZX= 0.2914

XY= 1.2207 YY= 25.3519 ZY= 1.3997

XZ= -0.7312 YZ= 0.1014 ZZ= 28.6260

Eigenvalues: 24.4418 26.8694 28.7900

27 H Isotropic = 25.6186 Anisotropy = 7.5764

XX= 24.7696 YX= -3.2032 ZX= -0.8504

XY= -3.2913 YY= 28.5845 ZY= 0.8388

XZ= -0.6524 YZ= 1.2365 ZZ= 23.5017

Eigenvalues: 22.8666 23.3196 30.6696

28 H Isotropic = 29.6365 Anisotropy = 4.7441

XX= 29.1288 YX= -1.3084 ZX= 1.2063

XY= -1.7064 YY= 28.8149 ZY= 2.4314

XZ= 1.1973 YZ= 2.9584 ZZ= 30.9657

Eigenvalues: 25.8581 30.2520 32.7992

29 H Isotropic = 29.1151 Anisotropy = 4.1541

XX= 30.3337 YX= 0.6674 ZX= -1.1068

XY= 1.2227 YY= 30.4973 ZY= -0.9271

XZ= -1.2689 YZ= -1.4297 ZZ= 26.5143

Eigenvalues: 25.9925 29.4683 31.8845

30 H Isotropic = 27.0621 Anisotropy = 6.3704

XX= 26.0344 YX= 0.4631 ZX= -0.6821

XY= 1.0814 YY= 26.0785 ZY= 2.9952

XZ= -0.7838 YZ= 3.8282 ZZ= 29.0735

Eigenvalues: 23.4284 26.4490 31.3091

31 H Isotropic = 29.5342 Anisotropy = 10.2452

XX= 31.8846 YX= -0.0338 ZX= 5.1454

XY= 0.7419 YY= 25.3988 ZY= -0.5423

XZ= 4.3186 YZ= -1.6331 ZZ= 31.3192

Eigenvalues: 24.8468 27.3914 36.3643

32 H Isotropic = 29.3112 Anisotropy = 7.6783

XX= 29.6841 YX= -1.2546 ZX= 0.5810

XY= -1.1470 YY= 33.8091 ZY= -2.0089

XZ= -0.5869 YZ= -1.5522 ZZ= 24.4404

Eigenvalues: 24.1045 29.3990 34.4300

33 H Isotropic = 30.2008 Anisotropy = 9.2991

XX= 29.2042 YX= -3.6679 ZX= -3.3159

XY= -2.4766 YY= 29.9554 ZY= 3.4090

XZ= -1.8718 YZ= 3.4642 ZZ= 31.4428

Eigenvalues: 26.4301 27.7721 36.4002

34 H Isotropic = 29.8495 Anisotropy = 9.0188

XX= 27.3656 YX= 0.1589 ZX= -0.4862

XY= 0.7004 YY= 34.5108 ZY= -3.0551

XZ= -1.0133 YZ= -3.4418 ZZ= 27.6719

Eigenvalues: 26.1318 27.5545 35.8620

35 H Isotropic = 29.4406 Anisotropy = 4.5237

XX= 30.5157 YX= 0.0330 ZX= 2.8994

XY= -1.1553 YY= 30.8214 ZY= 1.8733

XZ= 1.8230 YZ= 3.4049 ZZ= 26.9845

Eigenvalues: 24.6798 31.1855 32.4563

36 H Isotropic = 29.7533 Anisotropy = 6.4029

XX= 32.2459 YX= 1.4314 ZX= 2.8841

XY= 1.2096 YY= 28.9186 ZY= 0.3064

XZ= 2.3138 YZ= 1.7453 ZZ= 28.0955

Eigenvalues: 26.7741 28.4641 34.0219

37 H Isotropic = 29.3385 Anisotropy = 5.7549

XX= 27.5846 YX= -0.1027 ZX= -1.6507

XY= -0.2655 YY= 30.0691 ZY= 3.2425

XZ= -2.6209 YZ= 1.5934 ZZ= 30.3619

Eigenvalues: 26.1046 28.7359 33.1751

38 H Isotropic = 28.4224 Anisotropy = 9.7130

XX= 34.2854 YX= -0.8165 ZX= -2.0184

XY= -2.6935 YY= 26.6891 ZY= -0.2862

XZ= -1.3342 YZ= -0.5762 ZZ= 24.2927

Eigenvalues: 23.8264 26.5431 34.8977

39 H Isotropic = 30.6469 Anisotropy = 7.6554

XX= 30.8725 YX= -1.3438 ZX= -1.1012

XY= -1.8096 YY= 34.7327 ZY= -2.6140

XZ= -1.4211 YZ= -2.4997 ZZ= 26.3355

Eigenvalues: 25.1380 31.0522 35.7505

40 H Isotropic = 30.8029 Anisotropy = 8.4129

XX= 32.3421 YX= 2.8429 ZX= 3.7697

XY= 3.2592 YY= 31.1862 ZY= 1.4978

XZ= 3.1282 YZ= 0.6891 ZZ= 28.8803

Eigenvalues: 26.6152 29.3819 36.4115

41 H Isotropic = 31.1131 Anisotropy = 9.6520

XX= 30.8106 YX= 0.3170 ZX= -5.5851

XY= 1.6125 YY= 30.1951 ZY= -1.9784

XZ= -5.3200 YZ= -1.2687 ZZ= 32.3336

Eigenvalues: 26.0370 29.7546 37.5477

42 H Isotropic = 27.8304 Anisotropy = 7.0585

XX= 27.0039 YX= 1.0656 ZX= -0.2447

XY= 1.2242 YY= 27.6161 ZY= 5.1351

XZ= -0.0699 YZ= 3.2105 ZZ= 28.8712

Eigenvalues: 23.7321 27.2230 32.5360

43 H Isotropic = 26.0308 Anisotropy = 4.9475

XX= 26.2640 YX= 0.4072 ZX= -0.5093

XY= -0.5626 YY= 29.3225 ZY= -0.9691

XZ= 0.0872 YZ= 1.3164 ZZ= 22.5061

Eigenvalues: 22.4901 26.2733 29.3292

44 H Isotropic = 25.2851 Anisotropy = 4.5027

XX= 25.2708 YX= 2.0170 ZX= -2.9304

XY= 0.9904 YY= 24.2527 ZY= 0.0794

XZ= 0.1201 YZ= -2.8118 ZZ= 26.3318

Eigenvalues: 23.1325 24.4360 28.2869

45 H Isotropic = 28.4537 Anisotropy = 6.5400

XX= 27.2379 YX= 0.3084 ZX= -0.5157

XY= -1.7072 YY= 25.5728 ZY= -0.8019

XZ= -1.8064 YZ= -0.2722 ZZ= 32.5506

Eigenvalues: 25.2016 27.3459 32.8137

46 H Isotropic = 27.6060 Anisotropy = 9.8214

XX= 26.7367 YX= 1.8500 ZX= -0.9756

XY= 3.5747 YY= 28.9094 ZY= -4.9335

XZ= -1.0970 YZ= -5.0923 ZZ= 27.1720

Eigenvalues: 22.6805 25.9840 34.1536

47 H Isotropic = 27.6785 Anisotropy = 2.8384

XX= 27.7916 YX= -1.7766 ZX= 0.6355

XY= -3.1285 YY= 26.1676 ZY= 0.5078

XZ= 0.4313 YZ= 0.7891 ZZ= 29.0763

Eigenvalues: 24.2506 29.2141 29.5708

48 H Isotropic = 27.4925 Anisotropy = 7.5867

XX= 27.6538 YX= -2.7596 ZX= -2.5934

XY= -1.0107 YY= 31.5751 ZY= -2.0650

XZ= -2.8134 YZ= -2.8076 ZZ= 23.2485

Eigenvalues: 21.0952 28.8319 32.5503

49 H Isotropic = 28.3088 Anisotropy = 16.1691

XX= 27.2570 YX= -7.5296 ZX= 4.7972

XY= -8.1445 YY= 31.3509 ZY= -2.9322

XZ= 3.8352 YZ= -2.0272 ZZ= 26.3183

Eigenvalues: 20.4267 25.4114 39.0882

50 H Isotropic = 31.0822 Anisotropy = 20.3353

XX= 34.4678 YX= -6.1488 ZX= -9.8414

XY= -7.0467 YY= 26.8504 ZY= 2.5295

XZ= -8.7337 YZ= 0.5399 ZZ= 31.9285

Eigenvalues: 21.2465 27.3611 44.6391

Conformer 2

SCF GIAO Magnetic shielding tensor (ppm):

1 O Isotropic = -76.0299 Anisotropy = 572.1835

XX= 74.6209 YX= -169.1724 ZX= -172.4660

XY= -179.4051 YY= -160.6914 ZY= 171.8319

XZ= -170.9974 YZ= 222.8115 ZZ= -142.0191

Eigenvalues: -349.1092 -184.4062 305.4258

2 C Isotropic = 23.4878 Anisotropy = 84.3346

XX= 19.5055 YX= -58.1102 ZX= -34.4457

XY= -74.3876 YY= -3.1872 ZY= -27.1081

XZ= -15.5340 YZ= -3.2382 ZZ= 54.1450

Eigenvalues: -65.5048 56.2573 79.7108

3 O Isotropic = 124.2467 Anisotropy = 142.1101

XX= 153.3001 YX= -28.9232 ZX= -63.1235

XY= 36.0048 YY= 207.7225 ZY= 74.6846

XZ= -151.6267 YZ= -25.6089 ZZ= 11.7175

Eigenvalues: -48.2227 201.9761 218.9867

4 C Isotropic = 55.3457 Anisotropy = 124.3077

XX= 35.0009 YX= 37.0424 ZX= -80.8132

XY= 37.6337 YY= 67.1022 ZY= 3.1571

XZ= -77.1241 YZ= -2.5510 ZZ= 63.9341

Eigenvalues: -38.9334 66.7531 138.2175

5 C Isotropic = 115.6942 Anisotropy = 34.1587

XX= 129.7835 YX= 1.7860 ZX= 13.1609

XY= 4.3363 YY= 115.7858 ZY= 12.2534

XZ= 15.8044 YZ= 10.7886 ZZ= 101.5131

Eigenvalues: 91.5516 117.0643 138.4666

6 C Isotropic = 55.0030 Anisotropy = 142.7123

XX= 32.8149 YX= 20.9741 ZX= -93.4744

XY= 21.4342 YY= 64.3686 ZY= -20.0764

XZ= -87.6088 YZ= -11.0771 ZZ= 67.8256

Eigenvalues: -42.3003 57.1648 150.1446

7 C Isotropic = 149.2413 Anisotropy = 22.4506

XX= 157.4850 YX= -7.8313 ZX= -1.6774

XY= -10.3098 YY= 150.1227 ZY= 5.5480

XZ= -5.8016 YZ= -2.8793 ZZ= 140.1164

Eigenvalues: 139.3025 144.2131 164.2084

8 C Isotropic = 110.2439 Anisotropy = 45.7593

XX= 126.7563 YX= -10.7303 ZX= -23.1110

XY= -16.8430 YY= 103.6401 ZY= -15.1301

XZ= -21.8418 YZ= -9.0082 ZZ= 100.3353

Eigenvalues: 76.2778 113.7038 140.7501

9 C Isotropic = 152.9511 Anisotropy = 28.9868

XX= 162.8433 YX= -13.1377 ZX= -8.6607

XY= -7.8589 YY= 154.9739 ZY= -0.2969

XZ= -16.5784 YZ= -9.8350 ZZ= 141.0360

Eigenvalues: 131.5836 154.9940 172.2756

10 C Isotropic = 172.1736 Anisotropy = 24.7043

XX= 176.5063 YX= -12.9564 ZX= -0.8834

XY= -12.4369 YY= 174.7556 ZY= -5.3542

XZ= -1.7100 YZ= -4.8615 ZZ= 165.2590

Eigenvalues: 159.2307 168.6470 188.6432

11 C Isotropic = 148.4607 Anisotropy = 29.8352

XX= 150.9580 YX= -14.9585 ZX= 2.6502

XY= -18.4131 YY= 152.1841 ZY= -0.0798

XZ= 5.2023 YZ= 3.6052 ZZ= 142.2400

Eigenvalues: 133.0728 143.9585 168.3508

12 C Isotropic = 59.9632 Anisotropy = 127.4043

XX= 27.1935 YX= 76.2888 ZX= 4.4343

XY= 91.0218 YY= 76.6054 ZY= 17.5257

XZ= -14.4139 YZ= 38.8143 ZZ= 76.0907

Eigenvalues: -39.1750 74.1652 144.8994

13 N Isotropic = 196.2006 Anisotropy = 64.6736

XX= 189.2972 YX= -44.8214 ZX= -39.3848

XY= -36.9526 YY= 195.2934 ZY= -30.5649

XZ= -44.6592 YZ= -38.3938 ZZ= 204.0113

Eigenvalues: 117.3109 231.9746 239.3164

14 C Isotropic = 134.2555 Anisotropy = 63.7918

XX= 109.6378 YX= -17.6733 ZX= 5.1779

XY= -17.1423 YY= 172.0786 ZY= -1.2076

XZ= -5.4182 YZ= -5.3909 ZZ= 121.0499

Eigenvalues: 105.0562 120.9269 176.7833

15 C Isotropic = 175.3933 Anisotropy = 19.6078

XX= 182.8560 YX= 8.1649 ZX= -3.7668

XY= 9.0235 YY= 170.5683 ZY= -2.6855

XZ= -6.4531 YZ= 3.8827 ZZ= 172.7558

Eigenvalues: 164.9666 172.7483 188.4652

16 C Isotropic = 107.2989 Anisotropy = 27.2763

XX= 112.7041 YX= -8.5049 ZX= 15.2031

XY= 0.0185 YY= 100.0516 ZY= 12.0879

XZ= 13.4931 YZ= 2.1646 ZZ= 109.1411

Eigenvalues: 89.8868 106.5269 125.4831

17 C Isotropic = 132.4260 Anisotropy = 51.9400

XX= 150.0697 YX= 24.8221 ZX= -8.9155

XY= 21.9548 YY= 134.6830 ZY= 11.0301

XZ= -4.8800 YZ= 13.7179 ZZ= 112.5253

Eigenvalues: 100.8062 129.4192 167.0527

18 C Isotropic = 55.6397 Anisotropy = 115.1258

XX= 17.0014 YX= 69.5951 ZX= -7.2146

XY= 65.0899 YY= 68.8487 ZY= 51.5349

XZ= 9.0949 YZ= 17.9033 ZZ= 81.0690

Eigenvalues: -32.5387 67.0676 132.3902

19 C Isotropic = 125.8294 Anisotropy = 57.9161

XX= 108.2684 YX= -18.3042 ZX= 15.1913

XY= -20.2546 YY= 127.1193 ZY= -17.4630

XZ= 11.6174 YZ= -21.8206 ZZ= 142.1006

Eigenvalues: 96.2051 116.8430 164.4402

20 C Isotropic = 122.7371 Anisotropy = 44.2662

XX= 146.5475 YX= 15.4546 ZX= -9.6156

XY= 13.8812 YY= 112.9399 ZY= 0.0393

XZ= 0.6182 YZ= 7.2881 ZZ= 108.7240

Eigenvalues: 102.9533 113.0101 152.2479

21 O Isotropic = 287.5647 Anisotropy = 62.6541

XX= 257.4341 YX= 8.7561 ZX= -3.2625

XY= 0.9530 YY= 308.4122 ZY= -33.1058

XZ= 23.4892 YZ= -18.8457 ZZ= 296.8477

Eigenvalues: 252.3135 281.0465 329.3341

22 C Isotropic = 9.4429 Anisotropy = 96.3477

XX= -26.8961 YX= -52.2937 ZX= -48.9996

XY= -32.0026 YY= 47.9786 ZY= -5.3248

XZ= -69.8753 YZ= -12.7821 ZZ= 7.2461

Eigenvalues: -83.5181 38.1720 73.6747

23 O Isotropic = 130.7289 Anisotropy = 173.5559

XX= 162.4194 YX= -71.6015 ZX= -31.4407

XY= -128.7826 YY= 67.5110 ZY= 47.4941

XZ= 69.6648 YZ= 135.8542 ZZ= 162.2564

Eigenvalues: -35.5882 181.3422 246.4329

24 O Isotropic = 299.0000 Anisotropy = 52.4927

XX= 279.4551 YX= -5.1027 ZX= -15.2036

XY= 23.5227 YY= 290.2445 ZY= 16.3072

XZ= -10.3075 YZ= 13.0291 ZZ= 327.3006

Eigenvalues: 268.3123 294.6926 333.9952

25 O Isotropic = -41.1784 Anisotropy = 566.3279

XX= -23.7015 YX= -181.8443 ZX= -170.3174

XY= -153.6387 YY= 38.1119 ZY= 188.9193

XZ= -210.0017 YZ= 207.6720 ZZ= -137.9456

Eigenvalues: -298.4239 -161.4849 336.3735

26 H Isotropic = 26.7093 Anisotropy = 3.2395

XX= 26.4115 YX= 0.8430 ZX= 0.5412

XY= 1.2155 YY= 24.9472 ZY= 1.2301

XZ= -0.6785 YZ= -0.0043 ZZ= 28.7694

Eigenvalues: 24.3409 26.9180 28.8690

27 H Isotropic = 25.6076 Anisotropy = 7.7654

XX= 23.8847 YX= -2.7077 ZX= -0.7102

XY= -2.5536 YY= 29.5112 ZY= 0.9260

XZ= -0.3091 YZ= 1.4988 ZZ= 23.4269

Eigenvalues: 22.8440 23.1943 30.7845

28 H Isotropic = 29.7152 Anisotropy = 4.5083

XX= 28.6385 YX= -1.5214 ZX= 1.5027

XY= -1.8345 YY= 29.4609 ZY= 2.0527

XZ= 1.5264 YZ= 2.5744 ZZ= 31.0463

Eigenvalues: 25.9354 30.4894 32.7208

29 H Isotropic = 29.3110 Anisotropy = 3.9570

XX= 30.9701 YX= 0.5979 ZX= -1.0625

XY= 1.0096 YY= 30.2862 ZY= -0.5986

XZ= -1.3636 YZ= -1.2976 ZZ= 26.6765

Eigenvalues: 26.2258 29.7581 31.9490

30 H Isotropic = 27.0847 Anisotropy = 6.4055

XX= 26.2270 YX= 0.5382 ZX= -0.1266

XY= 0.9009 YY= 25.6755 ZY= 2.9977

XZ= -0.2073 YZ= 3.7263 ZZ= 29.3515

Eigenvalues: 23.4997 26.3993 31.3550

31 H Isotropic = 29.5383 Anisotropy = 10.2901

XX= 31.9247 YX= -1.3094 ZX= 4.9620

XY= -0.4071 YY= 25.5762 ZY= -1.4288

XZ= 4.1109 YZ= -2.2319 ZZ= 31.1139

Eigenvalues: 24.9644 27.2520 36.3984

32 H Isotropic = 29.3634 Anisotropy = 7.6060

XX= 29.4617 YX= -0.6260 ZX= 0.0769

XY= -0.4422 YY= 34.1880 ZY= -1.7382

XZ= -0.9194 YZ= -1.0942 ZZ= 24.4405

Eigenvalues: 24.1927 29.4634 34.4341

33 H Isotropic = 29.5068 Anisotropy = 4.2899

XX= 30.5108 YX= -0.0273 ZX= 2.8630

XY= -1.2440 YY= 30.7389 ZY= 1.4104

XZ= 2.2705 YZ= 3.3010 ZZ= 27.2708

Eigenvalues: 24.8878 31.2659 32.3668

34 H Isotropic = 30.2287 Anisotropy = 9.2111

XX= 28.3225 YX= -3.3276 ZX= -2.6472

XY= -2.2556 YY= 30.8466 ZY= 3.8203

XZ= -1.2898 YZ= 3.7374 ZZ= 31.5170

Eigenvalues: 26.4488 27.8678 36.3694

35 H Isotropic = 29.8019 Anisotropy = 8.9022

XX= 27.4433 YX= 1.2175 ZX= -1.0644

XY= 1.5844 YY= 34.1191 ZY= -2.9568

XZ= -1.3212 YZ= -3.1713 ZZ= 27.8433

Eigenvalues: 26.2215 27.4476 35.7367

36 H Isotropic = 29.6878 Anisotropy = 6.4813

XX= 32.6052 YX= 0.6545 ZX= 2.9874

XY= 0.9972 YY= 28.1466 ZY= -0.4998

XZ= 2.2859 YZ= 1.3954 ZZ= 28.3116

Eigenvalues: 27.0557 27.9991 34.0086

37 H Isotropic = 29.3345 Anisotropy = 5.7902

XX= 27.5641 YX= 0.4107 ZX= -1.0716

XY= 0.1360 YY= 29.8320 ZY= 3.5131

XZ= -2.2653 YZ= 1.9442 ZZ= 30.6073

Eigenvalues: 26.1280 28.6808 33.1947

38 H Isotropic = 28.4625 Anisotropy = 9.6766

XX= 33.5237 YX= -1.8258 ZX= -2.2145

XY= -3.7972 YY= 27.3507 ZY= 0.1436

XZ= -1.6261 YZ= -0.2886 ZZ= 24.5132

Eigenvalues: 23.9682 26.5058 34.9136

39 H Isotropic = 30.6780 Anisotropy = 8.0682

XX= 30.6269 YX= -1.0071 ZX= -1.1330

XY= -1.1528 YY= 35.4272 ZY= -2.3888

XZ= -2.0543 YZ= -2.2378 ZZ= 25.9799

Eigenvalues: 24.8783 31.0989 36.0568

40 H Isotropic = 30.9675 Anisotropy = 7.5455

XX= 33.3383 YX= 1.7984 ZX= 3.8942

XY= 2.1694 YY= 31.0361 ZY= 1.1068

XZ= 2.9030 YZ= 0.4217 ZZ= 28.5280

Eigenvalues: 26.7551 30.1495 35.9978

41 H Isotropic = 30.1450 Anisotropy = 8.9613

XX= 30.3250 YX= -0.4463 ZX= -5.4858

XY= 0.1300 YY= 29.1803 ZY= -1.4606

XZ= -5.3362 YZ= -0.7786 ZZ= 30.9298

Eigenvalues: 25.0180 29.2978 36.1192

42 H Isotropic = 27.7767 Anisotropy = 6.9065

XX= 27.1413 YX= 1.0076 ZX= 0.6920

XY= 1.2550 YY= 27.0495 ZY= 4.9055

XZ= 0.3660 YZ= 2.9208 ZZ= 29.1394

Eigenvalues: 23.9360 27.0132 32.3811

43 H Isotropic = 26.1427 Anisotropy = 5.2777

XX= 26.2692 YX= 1.1108 ZX= -0.5208

XY= -0.0323 YY= 29.5528 ZY= -0.6717

XZ= 0.2504 YZ= 1.5129 ZZ= 22.6060

Eigenvalues: 22.5729 26.1939 29.6611

44 H Isotropic = 25.2965 Anisotropy = 4.6744

XX= 25.6754 YX= 1.9009 ZX= -2.8281

XY= 1.0675 YY= 23.9005 ZY= 0.3591

XZ= -0.2731 YZ= -2.9326 ZZ= 26.3136

Eigenvalues: 23.0014 24.4753 28.4128

45 H Isotropic = 28.4700 Anisotropy = 6.6433

XX= 26.9114 YX= 0.0511 ZX= -0.5469

XY= -1.7806 YY= 25.8788 ZY= -0.8645

XZ= -1.9157 YZ= -0.3358 ZZ= 32.6198

Eigenvalues: 25.2092 27.3020 32.8989

46 H Isotropic = 27.6050 Anisotropy = 9.9980

XX= 27.5041 YX= 2.0697 ZX= -1.5890

XY= 4.0798 YY= 28.3161 ZY= -4.7302

XZ= -1.8730 YZ= -4.8565 ZZ= 26.9947

Eigenvalues: 22.6750 25.8696 34.2703

47 H Isotropic = 28.1747 Anisotropy = 6.3743

XX= 30.1574 YX= 2.4877 ZX= -1.4892

XY= 0.0589 YY= 31.5936 ZY= 1.9134

XZ= -1.8413 YZ= 1.9302 ZZ= 22.7731

Eigenvalues: 21.9338 30.1660 32.4242

48 H Isotropic = 27.9839 Anisotropy = 3.2026

XX= 29.1978 YX= 0.6156 ZX= 0.5588

XY= 0.4004 YY= 25.3091 ZY= 1.9302

XZ= -0.4746 YZ= 1.5136 ZZ= 29.4447

Eigenvalues: 24.6389 29.1938 30.1189

49 H Isotropic = 28.7314 Anisotropy = 18.9627

XX= 24.2112 YX= 2.9074 ZX= 2.2705

XY= 3.6168 YY= 34.0799 ZY= -10.0488

XZ= 0.8259 YZ= -9.4032 ZZ= 27.9032

Eigenvalues: 18.8561 25.9650 41.3732

50 H Isotropic = 29.1324 Anisotropy = 12.6883

XX= 30.4943 YX= 1.5315 ZX= 0.9540

XY= -0.2643 YY= 19.7934 ZY= 3.3492

XZ= 0.2819 YZ= 2.0517 ZZ= 37.1095

Eigenvalues: 19.3565 30.4495 37.5913

Conformer 3

SCF GIAO Magnetic shielding tensor (ppm):

1 O Isotropic = -76.8579 Anisotropy = 576.7809

XX= 121.9142 YX= -135.0084 ZX= -192.1798

XY= -141.9008 YY= -201.3212 ZY= 144.9814

XZ= -200.5512 YZ= 200.8697 ZZ= -151.1667

Eigenvalues: -352.7955 -185.4410 307.6627

2 C Isotropic = 23.6113 Anisotropy = 85.1055

XX= 37.6065 YX= -52.6994 ZX= -31.0338

XY= -69.2643 YY= -21.9730 ZY= -31.0310

XZ= -13.1718 YZ= -3.0501 ZZ= 55.2005

Eigenvalues: -65.7426 56.2283 80.3483

3 O Isotropic = 124.0958 Anisotropy = 144.3017

XX= 153.3180 YX= -38.9314 ZX= -73.7919

XY= 23.3457 YY= 206.2537 ZY= 62.5477

XZ= -146.1398 YZ= -50.4423 ZZ= 12.7157

Eigenvalues: -47.5100 199.5005 220.2970

4 C Isotropic = 55.8743 Anisotropy = 120.6383

XX= 22.7589 YX= 28.6649 ZX= -79.0621

XY= 28.3636 YY= 78.1142 ZY= -10.0392

XZ= -73.6864 YZ= -12.5078 ZZ= 66.7499

Eigenvalues: -37.0333 68.3564 136.2999

5 C Isotropic = 115.6694 Anisotropy = 34.4476

XX= 127.8067 YX= 3.6485 ZX= 11.3636

XY= 6.6557 YY= 117.6654 ZY= 14.2104

XZ= 14.7862 YZ= 13.0766 ZZ= 101.5360

Eigenvalues: 91.3954 116.9783 138.6345

6 C Isotropic = 56.6143 Anisotropy = 141.3139

XX= 26.8565 YX= 12.2718 ZX= -90.6479

XY= 15.3750 YY= 72.0775 ZY= -32.7584

XZ= -85.7794 YZ= -20.3285 ZZ= 70.9089

Eigenvalues: -42.3096 61.3289 150.8236

7 C Isotropic = 149.0605 Anisotropy = 21.3253

XX= 158.8812 YX= -6.4787 ZX= -3.1463

XY= -8.7893 YY= 147.9946 ZY= 5.1603

XZ= -2.5447 YZ= -1.9558 ZZ= 140.3058

Eigenvalues: 139.8388 144.0654 163.2774

8 C Isotropic = 110.4009 Anisotropy = 46.8421

XX= 130.9484 YX= -6.8633 ZX= -20.4256

XY= -14.2978 YY= 99.2280 ZY= -17.9024

XZ= -20.7839 YZ= -12.4895 ZZ= 101.0264

Eigenvalues: 75.9857 113.5881 141.6290

9 C Isotropic = 152.4775 Anisotropy = 28.6825

XX= 165.3071 YX= -11.6588 ZX= -7.6524

XY= -7.2285 YY= 151.7911 ZY= -1.9806

XZ= -13.8977 YZ= -13.3707 ZZ= 140.3343

Eigenvalues: 130.8239 155.0095 171.5992

10 C Isotropic = 173.1734 Anisotropy = 23.3315

XX= 180.7682 YX= -11.6475 ZX= -1.1554

XY= -11.5013 YY= 171.3869 ZY= -6.2238

XZ= -2.3928 YZ= -5.4758 ZZ= 167.3651

Eigenvalues: 159.2983 171.4942 188.7278

11 C Isotropic = 151.7861 Anisotropy = 24.6105

XX= 159.9156 YX= -10.5242 ZX= 1.5042

XY= -11.8180 YY= 152.4819 ZY= -1.9051

XZ= 6.3814 YZ= 4.8464 ZZ= 142.9608

Eigenvalues: 139.9993 147.1659 168.1931

12 C Isotropic = 60.1230 Anisotropy = 128.0033

XX= 5.8248 YX= 65.8570 ZX= -0.2241

XY= 80.5131 YY= 100.6214 ZY= 17.9011

XZ= -22.6533 YZ= 36.8647 ZZ= 73.9228

Eigenvalues: -38.7989 73.7093 145.4585

13 N Isotropic = 196.0526 Anisotropy = 67.3098

XX= 201.5848 YX= -44.3556 ZX= -34.5006

XY= -36.9034 YY= 181.0295 ZY= -34.3235

XZ= -40.1040 YZ= -41.7587 ZZ= 205.5434

Eigenvalues: 117.4703 229.7616 240.9257

14 C Isotropic = 134.7751 Anisotropy = 63.9541

XX= 115.9690 YX= -25.7759 ZX= 6.1130

XY= -25.6770 YY= 166.0815 ZY= -2.3959

XZ= -3.8002 YZ= -7.7238 ZZ= 122.2747

Eigenvalues: 105.0651 121.8490 177.4112

15 C Isotropic = 176.7438 Anisotropy = 22.9227

XX= 180.5469 YX= 11.2366 ZX= -3.7712

XY= 13.8519 YY= 176.1676 ZY= -2.9581

XZ= -5.9201 YZ= 1.6520 ZZ= 173.5169

Eigenvalues: 164.7764 173.4294 192.0256

16 C Isotropic = 106.0649 Anisotropy = 23.3128

XX= 113.4313 YX= -2.5480 ZX= 12.8089

XY= -4.9469 YY= 101.4157 ZY= 11.3023

XZ= 9.7638 YZ= 13.1119 ZZ= 103.3476

Eigenvalues: 86.0095 110.5784 121.6067

17 C Isotropic = 131.8678 Anisotropy = 52.2899

XX= 142.3494 YX= 25.6881 ZX= -10.4348

XY= 22.9268 YY= 142.4908 ZY= 6.9683

XZ= -7.6831 YZ= 11.0308 ZZ= 110.7631

Eigenvalues: 101.1502 127.7254 166.7277

18 C Isotropic = 55.5523 Anisotropy = 113.9961

XX= -0.4429 YX= 59.4583 ZX= -17.6159

XY= 56.4500 YY= 87.6550 ZY= 49.5611

XZ= 4.3853 YZ= 18.0592 ZZ= 79.4450

Eigenvalues: -33.2629 68.3702 131.5497

19 C Isotropic = 126.5240 Anisotropy = 59.1300

XX= 113.3984 YX= -19.8298 ZX= 18.3728

XY= -21.4307 YY= 120.9201 ZY= -14.7472

XZ= 15.5359 YZ= -19.3522 ZZ= 145.2536

Eigenvalues: 96.0652 117.5628 165.9440

20 C Isotropic = 122.1156 Anisotropy = 42.4552

XX= 117.6829 YX= -24.9848 ZX= -4.5206

XY= -21.9506 YY= 133.2971 ZY= -1.3700

XZ= -0.4786 YZ= -8.6681 ZZ= 115.3670

Eigenvalues: 99.2195 116.7083 150.4191

21 O Isotropic = 286.7638 Anisotropy = 39.2259

XX= 276.9506 YX= 36.5251 ZX= -0.8023

XY= 25.1137 YY= 286.1097 ZY= 6.7604

XZ= 13.6927 YZ= -13.0237 ZZ= 297.2310

Eigenvalues: 249.3690 298.0079 312.9143

22 C Isotropic = 10.3728 Anisotropy = 94.8242

XX= -14.7582 YX= -64.5659 ZX= -41.4138

XY= -42.9593 YY= 32.4392 ZY= -8.9101

XZ= -64.7494 YZ= -24.8195 ZZ= 13.4376

Eigenvalues: -80.8945 38.4240 73.5890

23 O Isotropic = 136.8984 Anisotropy = 168.2244

XX= 191.7428 YX= -64.0829 ZX= -27.0846

XY= -111.1538 YY= 65.5693 ZY= 43.4965

XZ= 68.3351 YZ= 151.7718 ZZ= 153.3830

Eigenvalues: -30.4661 192.1133 249.0480

24 O Isotropic = 307.7779 Anisotropy = 47.7747

XX= 281.5993 YX= 24.4467 ZX= 8.7071

XY= -13.2084 YY= 309.0012 ZY= 7.5521

XZ= 24.1329 YZ= 5.7159 ZZ= 332.7331

Eigenvalues: 276.3875 307.3185 339.6277

25 O Isotropic = -61.3656 Anisotropy = 600.2083

XX= 12.2784 YX= -195.9918 ZX= -207.3067

XY= -181.0494 YY= 13.7215 ZY= 158.2533

XZ= -233.4233 YZ= 175.5139 ZZ= -210.0968

Eigenvalues: -353.4067 -169.4635 338.7733

26 H Isotropic = 26.7045 Anisotropy = 3.1905

XX= 26.1316 YX= 0.9668 ZX= 0.3452

XY= 1.2113 YY= 25.3002 ZY= 1.3638

XZ= -0.7500 YZ= 0.0899 ZZ= 28.6817

Eigenvalues: 24.4298 26.8522 28.8315

27 H Isotropic = 25.6404 Anisotropy = 7.6141

XX= 24.7082 YX= -3.2131 ZX= -0.8635

XY= -3.2640 YY= 28.6676 ZY= 0.8354

XZ= -0.6604 YZ= 1.2726 ZZ= 23.5455

Eigenvalues: 22.8460 23.3588 30.7165

28 H Isotropic = 29.7522 Anisotropy = 4.6253

XX= 29.1154 YX= -1.3099 ZX= 1.1822

XY= -1.6510 YY= 29.1644 ZY= 2.4453

XZ= 1.1716 YZ= 2.7723 ZZ= 30.9770

Eigenvalues: 26.1182 30.3027 32.8358

29 H Isotropic = 29.3413 Anisotropy = 3.9340

XX= 30.6639 YX= 0.4876 ZX= -1.1303

XY= 1.0523 YY= 30.6929 ZY= -0.8658

XZ= -1.2722 YZ= -1.4063 ZZ= 26.6672

Eigenvalues: 26.1509 29.9091 31.9640

30 H Isotropic = 27.0624 Anisotropy = 6.3995

XX= 26.0251 YX= 0.5211 ZX= -0.5846

XY= 1.0939 YY= 26.0071 ZY= 2.9772

XZ= -0.7188 YZ= 3.8195 ZZ= 29.1549

Eigenvalues: 23.4262 26.4322 31.3287

31 H Isotropic = 29.5318 Anisotropy = 10.2592

XX= 31.9510 YX= -0.1504 ZX= 5.1504

XY= 0.6417 YY= 25.3837 ZY= -0.6370

XZ= 4.2862 YZ= -1.6911 ZZ= 31.2608

Eigenvalues: 24.8399 27.3843 36.3713

32 H Isotropic = 29.3107 Anisotropy = 7.6689

XX= 29.6522 YX= -1.2010 ZX= 0.5220

XY= -1.1023 YY= 33.8432 ZY= -1.9878

XZ= -0.6564 YZ= -1.5185 ZZ= 24.4366

Eigenvalues: 24.1070 29.4017 34.4233

33 H Isotropic = 30.2088 Anisotropy = 9.2862

XX= 29.1214 YX= -3.6322 ZX= -3.2476

XY= -2.4428 YY= 30.0184 ZY= 3.4634

XZ= -1.8206 YZ= 3.5014 ZZ= 31.4865

Eigenvalues: 26.4381 27.7886 36.3996

34 H Isotropic = 29.8507 Anisotropy = 9.0243

XX= 27.3490 YX= 0.2091 ZX= -0.5189

XY= 0.7341 YY= 34.5030 ZY= -3.0639

XZ= -1.0402 YZ= -3.4317 ZZ= 27.7002

Eigenvalues: 26.1386 27.5467 35.8669

35 H Isotropic = 29.4685 Anisotropy = 4.4818

XX= 30.5830 YX= 0.0559 ZX= 2.8778

XY= -1.1457 YY= 30.8183 ZY= 1.8447

XZ= 1.7878 YZ= 3.3951 ZZ= 27.0043

Eigenvalues: 24.7380 31.2112 32.4564

36 H Isotropic = 29.8702 Anisotropy = 6.1147

XX= 32.3540 YX= 1.2281 ZX= 2.9178

XY= 0.9767 YY= 28.9200 ZY= 0.0348

XZ= 2.1318 YZ= 1.7068 ZZ= 28.3367

Eigenvalues: 27.0597 28.6043 33.9467

37 H Isotropic = 29.3554 Anisotropy = 5.7391

XX= 27.5720 YX= -0.0504 ZX= -1.5893

XY= -0.2065 YY= 30.0647 ZY= 3.2610

XZ= -2.5824 YZ= 1.6049 ZZ= 30.4295

Eigenvalues: 26.1285 28.7562 33.1815

38 H Isotropic = 28.4250 Anisotropy = 9.7148

XX= 34.2184 YX= -0.9155 ZX= -2.0945

XY= -2.7893 YY= 26.7265 ZY= -0.2355

XZ= -1.4219 YZ= -0.5678 ZZ= 24.3302

Eigenvalues: 23.8329 26.5405 34.9016

39 H Isotropic = 30.6407 Anisotropy = 7.7569

XX= 30.7874 YX= -1.3214 ZX= -1.0820

XY= -1.6996 YY= 34.9236 ZY= -2.4903

XZ= -1.5437 YZ= -2.3093 ZZ= 26.2110

Eigenvalues: 25.1053 31.0048 35.8120

40 H Isotropic = 30.9472 Anisotropy = 8.3498

XX= 32.6701 YX= 2.7916 ZX= 3.7573

XY= 3.2154 YY= 31.2159 ZY= 1.4239

XZ= 3.0372 YZ= 0.5993 ZZ= 28.9556

Eigenvalues: 26.8038 29.5241 36.5137

41 H Isotropic = 30.9855 Anisotropy = 9.6902

XX= 30.5943 YX= 0.3903 ZX= -5.5843

XY= 1.6563 YY= 30.0958 ZY= -1.9465

XZ= -5.3854 YZ= -1.1789 ZZ= 32.2665

Eigenvalues: 25.8673 29.6437 37.4457

42 H Isotropic = 27.6979 Anisotropy = 7.0403

XX= 26.8492 YX= 1.1128 ZX= -0.0886

XY= 1.2836 YY= 27.5286 ZY= 5.1196

XZ= -0.0936 YZ= 3.1452 ZZ= 28.7158

Eigenvalues: 23.6506 27.0515 32.3914

43 H Isotropic = 26.0887 Anisotropy = 5.1292

XX= 26.2422 YX= 0.5452 ZX= -0.5639

XY= -0.3793 YY= 29.5039 ZY= -1.0224

XZ= 0.0402 YZ= 1.2817 ZZ= 22.5199

Eigenvalues: 22.4990 26.2589 29.5081

44 H Isotropic = 25.3074 Anisotropy = 4.4784

XX= 25.3165 YX= 2.0423 ZX= -2.8578

XY= 1.0583 YY= 24.2324 ZY= 0.1121

XZ= 0.1421 YZ= -2.8110 ZZ= 26.3733

Eigenvalues: 23.0874 24.5418 28.2930

45 H Isotropic = 28.4718 Anisotropy = 6.5795

XX= 27.2238 YX= 0.3023 ZX= -0.4809

XY= -1.7279 YY= 25.5813 ZY= -0.8153

XZ= -1.7677 YZ= -0.2909 ZZ= 32.6102

Eigenvalues: 25.1979 27.3593 32.8581

46 H Isotropic = 27.6130 Anisotropy = 9.8456

XX= 26.7958 YX= 1.8624 ZX= -1.0198

XY= 3.5926 YY= 28.8636 ZY= -4.9587

XZ= -1.1317 YZ= -5.0996 ZZ= 27.1797

Eigenvalues: 22.6617 26.0006 34.1767

47 H Isotropic = 28.0521 Anisotropy = 3.2356

XX= 28.2712 YX= -1.3338 ZX= -0.1778

XY= -2.0235 YY= 26.0959 ZY= 0.8380

XZ= -0.2523 YZ= 0.8419 ZZ= 29.7893

Eigenvalues: 25.0957 28.8514 30.2092

48 H Isotropic = 27.5639 Anisotropy = 7.7135

XX= 27.6356 YX= -2.3473 ZX= -2.5782

XY= -1.0251 YY= 31.6757 ZY= -2.7972

XZ= -3.4286 YZ= -2.9661 ZZ= 23.3802

Eigenvalues: 20.8043 29.1810 32.7062

49 H Isotropic = 28.2176 Anisotropy = 17.7002

XX= 23.3276 YX= 3.6550 ZX= 0.6949

XY= 3.1602 YY= 31.6254 ZY= -8.9737

XZ= 0.4439 YZ= -9.0648 ZZ= 29.6998

Eigenvalues: 19.5405 25.0946 40.0177

50 H Isotropic = 30.0065 Anisotropy = 14.5007

XX= 32.5775 YX= -3.7261 ZX= 7.5039

XY= -2.0846 YY= 24.7561 ZY= 2.9380

XZ= 6.5108 YZ= 4.7332 ZZ= 32.6859

Eigenvalues: 20.3935 29.9525 39.6736

Conformer 4

SCF GIAO Magnetic shielding tensor (ppm):

1 O Isotropic = -73.9202 Anisotropy = 571.0974

XX= 85.4165 YX= 180.4945 ZX= -161.0509

XY= 189.2235 YY= -147.9899 ZY= -166.7750

XZ= -158.3222 YZ= -215.8753 ZZ= -159.1873

Eigenvalues: -345.7295 -182.8425 306.8113

2 C Isotropic = 23.3153 Anisotropy = 84.5999

XX= 20.7609 YX= 59.3591 ZX= -33.4841

XY= 73.9850 YY= -4.0043 ZY= 27.5731

XZ= -14.6214 YZ= 3.2469 ZZ= 53.1891

Eigenvalues: -65.6968 55.9274 79.7152

3 O Isotropic = 122.8969 Anisotropy = 144.5580

XX= 158.9943 YX= 31.4591 ZX= -58.5118

XY= -28.5246 YY= 207.3289 ZY= -70.5331

XZ= -148.3514 YZ= 32.2480 ZZ= 2.3676

Eigenvalues: -50.1112 199.5331 219.2689

4 C Isotropic = 54.5136 Anisotropy = 125.1870

XX= 39.9445 YX= -34.8558 ZX= -83.1357

XY= -34.7400 YY= 66.1582 ZY= -4.1372

XZ= -79.0279 YZ= 3.1688 ZZ= 57.4382

Eigenvalues: -39.5572 65.1264 137.9716

5 C Isotropic = 115.8166 Anisotropy = 34.4095

XX= 129.0759 YX= -2.0843 ZX= 13.8424

XY= -4.7606 YY= 116.8464 ZY= -12.5474

XZ= 16.2845 YZ= -10.5909 ZZ= 101.5275

Eigenvalues: 91.3731 117.3204 138.7563

6 C Isotropic = 55.7160 Anisotropy = 141.3210

XX= 39.4931 YX= -19.1325 ZX= -94.3111

XY= -19.3015 YY= 64.8826 ZY= 19.1569

XZ= -88.4134 YZ= 10.5537 ZZ= 62.7723

Eigenvalues: -41.1738 58.3918 149.9300

7 C Isotropic = 149.8180 Anisotropy = 21.2933

XX= 158.7829 YX= 7.8157 ZX= -0.6747

XY= 8.9542 YY= 150.1984 ZY= -3.9962

XZ= -1.7468 YZ= 1.9799 ZZ= 140.4729

Eigenvalues: 140.3586 145.0819 164.0136

8 C Isotropic = 110.0039 Anisotropy = 46.2258

XX= 128.8110 YX= 11.0323 ZX= -22.2882

XY= 15.6926 YY= 102.2819 ZY= 15.5108

XZ= -20.0069 YZ= 10.5293 ZZ= 98.9187

Eigenvalues: 75.8568 113.3338 140.8211

9 C Isotropic = 152.9758 Anisotropy = 28.2037

XX= 163.4841 YX= 12.5633 ZX= -7.5825

XY= 7.5809 YY= 154.8018 ZY= 0.5801

XZ= -15.3530 YZ= 10.2408 ZZ= 140.6414

Eigenvalues: 132.1716 154.9774 171.7783

10 C Isotropic = 172.5253 Anisotropy = 24.1252

XX= 176.8892 YX= 12.6796 ZX= -0.6215

XY= 12.1356 YY= 174.4406 ZY= 5.9948

XZ= -1.2732 YZ= 5.6033 ZZ= 166.2460

Eigenvalues: 159.4301 169.5369 188.6087

11 C Isotropic = 153.5700 Anisotropy = 21.9115

XX= 158.3159 YX= 9.8175 ZX= -1.5946

XY= 9.4986 YY= 158.7068 ZY= 4.0760

XZ= 3.9657 YZ= -5.3084 ZZ= 143.6872

Eigenvalues: 143.3824 149.1499 168.1776

12 C Isotropic = 58.7194 Anisotropy = 128.4561

XX= 22.2062 YX= -74.2937 ZX= 0.8030

XY= -89.0061 YY= 79.8654 ZY= -22.8887

XZ= -18.6328 YZ= -41.5719 ZZ= 74.0866

Eigenvalues: -41.4832 73.2846 144.3568

13 N Isotropic = 196.6935 Anisotropy = 64.0409

XX= 193.4214 YX= 43.0199 ZX= -38.2703

XY= 36.7445 YY= 194.5787 ZY= 33.6139

XZ= -44.3927 YZ= 40.5220 ZZ= 202.0804

Eigenvalues: 117.6583 233.0348 239.3874

14 C Isotropic = 134.1320 Anisotropy = 63.2753

XX= 109.4109 YX= 17.8023 ZX= 5.0019

XY= 17.4859 YY= 171.2998 ZY= 2.6272

XZ= -6.3192 YZ= 6.6219 ZZ= 121.6854

Eigenvalues: 104.5376 121.5428 176.3155

15 C Isotropic = 176.9396 Anisotropy = 24.2483

XX= 184.1104 YX= -11.9294 ZX= -3.4873

XY= -14.5979 YY= 172.6022 ZY= 0.5150

XZ= -4.3634 YZ= -4.0126 ZZ= 174.1060

Eigenvalues: 162.7307 174.9830 193.1051

16 C Isotropic = 108.9917 Anisotropy = 27.4664

XX= 106.7899 YX= 6.6514 ZX= 12.0648

XY= 3.1946 YY= 103.1955 ZY= -7.8641

XZ= 16.7947 YZ= -2.9191 ZZ= 116.9896

Eigenvalues: 92.0092 107.6632 127.3026

17 C Isotropic = 133.6940 Anisotropy = 51.5093

XX= 150.0535 YX= -24.9628 ZX= -7.1809

XY= -21.9701 YY= 136.5845 ZY= -12.0308

XZ= -3.7941 YZ= -15.3583 ZZ= 114.4439

Eigenvalues: 102.1298 130.9187 168.0335

18 C Isotropic = 57.5927 Anisotropy = 112.5878

XX= 15.4518 YX= -66.2335 ZX= -13.5030

XY= -59.4851 YY= 75.1238 ZY= -53.5522

XZ= 3.4711 YZ= -21.0015 ZZ= 82.2024

Eigenvalues: -29.7874 69.9141 132.6512

19 C Isotropic = 125.9902 Anisotropy = 57.9152

XX= 108.3186 YX= 17.5946 ZX= 13.7787

XY= 19.0055 YY= 126.1050 ZY= 19.0094

XZ= 10.3855 YZ= 22.1550 ZZ= 143.5468

Eigenvalues: 96.8513 116.5189 164.6003

20 C Isotropic = 122.3766 Anisotropy = 57.4387

XX= 109.3778 YX= 5.6847 ZX= 3.7602

XY= 4.6555 YY= 97.8158 ZY= -6.1432

XZ= 8.2879 YZ= 2.4140 ZZ= 159.9362

Eigenvalues: 95.5999 110.8608 160.6691

21 O Isotropic = 274.6419 Anisotropy = 34.6880

XX= 251.1726 YX= -21.1687 ZX= 0.2939

XY= -11.0769 YY= 277.5520 ZY= -1.1414

XZ= 19.4601 YZ= 13.2356 ZZ= 295.2011

Eigenvalues: 241.0801 285.0784 297.7672

22 C Isotropic = 8.5602 Anisotropy = 97.2628

XX= -30.1778 YX= 50.9999 ZX= -51.7334

XY= 31.3905 YY= 46.2812 ZY= 4.1962

XZ= -71.9892 YZ= 11.0334 ZZ= 9.5772

Eigenvalues: -86.2367 38.5153 73.4021

23 O Isotropic = 125.2793 Anisotropy = 177.8711

XX= 166.1261 YX= 68.6501 ZX= -38.8845

XY= 124.3653 YY= 39.9319 ZY= -47.1487

XZ= 55.5704 YZ= -130.4842 ZZ= 169.7800

Eigenvalues: -44.4290 176.4070 243.8601

24 O Isotropic = 302.9415 Anisotropy = 121.8158

XX= 320.2517 YX= 27.2387 ZX= -66.9660

XY= 26.2433 YY= 268.5653 ZY= -31.5679

XZ= -37.7822 YZ= -18.7252 ZZ= 320.0075

Eigenvalues: 256.8203 267.8522 384.1520

25 O Isotropic = -33.8477 Anisotropy = 557.4058

XX= -24.9875 YX= 172.2155 ZX= -183.7622

XY= 136.4678 YY= 5.6871 ZY= -188.1279

XZ= -223.4393 YZ= -209.3824 ZZ= -82.2426

Eigenvalues: -276.2441 -163.0552 337.7562

26 H Isotropic = 26.6064 Anisotropy = 3.0676

XX= 26.3532 YX= -0.8858 ZX= 0.3859

XY= -1.1350 YY= 24.9437 ZY= -1.2921

XZ= -0.7981 YZ= -0.0914 ZZ= 28.5222

Eigenvalues: 24.2975 26.8702 28.6514

27 H Isotropic = 25.6318 Anisotropy = 7.7474

XX= 23.9625 YX= 2.7562 ZX= -0.6698

XY= 2.6155 YY= 29.5742 ZY= -0.6754

XZ= -0.2311 YZ= -1.1941 ZZ= 23.3585

Eigenvalues: 22.8703 23.2283 30.7967

28 H Isotropic = 29.6911 Anisotropy = 4.8105

XX= 28.3657 YX= 1.5258 ZX= 1.4015

XY= 1.8038 YY= 29.3836 ZY= -1.9400

XZ= 1.1159 YZ= -2.7557 ZZ= 31.3239

Eigenvalues: 25.9868 30.1883 32.8981

29 H Isotropic = 29.1968 Anisotropy = 4.5768

XX= 31.1391 YX= -0.9067 ZX= -0.8345

XY= -1.4543 YY= 30.1106 ZY= 0.6977

XZ= -1.3589 YZ= 1.0891 ZZ= 26.3407

Eigenvalues: 25.9995 29.3429 32.2480

30 H Isotropic = 27.0234 Anisotropy = 6.3452

XX= 26.2460 YX= -0.3941 ZX= -0.4066

XY= -0.8254 YY= 25.7683 ZY= -3.1679

XZ= -0.4431 YZ= -3.7754 ZZ= 29.0560

Eigenvalues: 23.3788 26.4380 31.2536

31 H Isotropic = 29.5442 Anisotropy = 10.2716

XX= 31.7350 YX= 1.0658 ZX= 5.0054

XY= 0.2433 YY= 25.4955 ZY= 1.2469

XZ= 4.1316 YZ= 2.1556 ZZ= 31.4021

Eigenvalues: 24.9601 27.2805 36.3919

32 H Isotropic = 29.4030 Anisotropy = 7.6370

XX= 29.5271 YX= 0.6392 ZX= 0.2304

XY= 0.5192 YY= 34.1841 ZY= 1.9220

XZ= -0.7854 YZ= 1.2555 ZZ= 24.4979

Eigenvalues: 24.2187 29.4960 34.4944

33 H Isotropic = 30.2369 Anisotropy = 9.2343

XX= 28.4839 YX= 3.5450 ZX= -2.6701

XY= 2.4598 YY= 31.0501 ZY= -3.7271

XZ= -1.3280 YZ= -3.6551 ZZ= 31.1768

Eigenvalues: 26.4380 27.8796 36.3931

34 H Isotropic = 29.8357 Anisotropy = 8.8939

XX= 27.6214 YX= -1.2890 ZX= -1.0831

XY= -1.6446 YY= 33.9797 ZY= 3.0925

XZ= -1.4202 YZ= 3.2865 ZZ= 27.9061

Eigenvalues: 26.2205 27.5217 35.7650

35 H Isotropic = 29.4623 Anisotropy = 4.3801

XX= 30.2881 YX= 0.0066 ZX= 3.0435

XY= 1.2046 YY= 30.8456 ZY= -1.2814

XZ= 2.4240 YZ= -3.2152 ZZ= 27.2532

Eigenvalues: 24.8026 31.2019 32.3824

36 H Isotropic = 29.2711 Anisotropy = 5.3386

XX= 31.5097 YX= -0.1750 ZX= 2.3589

XY= -0.4291 YY= 27.8967 ZY= -0.3786

XZ= 2.1677 YZ= -1.8390 ZZ= 28.4069

Eigenvalues: 26.6160 28.3672 32.8302

37 H Isotropic = 29.3102 Anisotropy = 5.7827

XX= 27.6496 YX= -0.3837 ZX= -1.1472

XY= -0.0287 YY= 29.8846 ZY= -3.4999

XZ= -2.3524 YZ= -2.0147 ZZ= 30.3964

Eigenvalues: 26.0811 28.6841 33.1654

38 H Isotropic = 28.4556 Anisotropy = 9.6519

XX= 33.6106 YX= 1.7794 ZX= -1.9952

XY= 3.7797 YY= 27.3328 ZY= -0.0160

XZ= -1.4173 YZ= 0.3713 ZZ= 24.4234

Eigenvalues: 23.9491 26.5274 34.8902

39 H Isotropic = 30.6139 Anisotropy = 8.0867

XX= 30.4985 YX= 0.2719 ZX= -0.9333

XY= 0.8099 YY= 35.3060 ZY= 2.6123

XZ= -1.5327 YZ= 2.6341 ZZ= 26.0373

Eigenvalues: 25.0230 30.8137 36.0051

40 H Isotropic = 30.9466 Anisotropy = 8.7089

XX= 32.6702 YX= -2.6220 ZX= 4.3657

XY= -2.9223 YY= 30.1714 ZY= -1.2721

XZ= 3.4041 YZ= -1.0322 ZZ= 29.9983

Eigenvalues: 27.0149 29.0725 36.7526

41 H Isotropic = 30.5916 Anisotropy = 9.5267

XX= 31.7641 YX= -0.9996 ZX= -5.6717

XY= -1.6238 YY= 29.4680 ZY= 0.3710

XZ= -5.5739 YZ= -0.3170 ZZ= 30.5428

Eigenvalues: 25.3173 29.5148 36.9428

42 H Isotropic = 27.7757 Anisotropy = 6.8196

XX= 27.1624 YX= -0.9810 ZX= 0.4217

XY= -1.1657 YY= 27.0680 ZY= -4.8793

XZ= 0.4060 YZ= -2.9634 ZZ= 29.0967

Eigenvalues: 23.9186 27.0864 32.3221

43 H Isotropic = 25.9489 Anisotropy = 4.8334

XX= 26.3386 YX= -1.0100 ZX= -0.2391

XY= 0.1715 YY= 29.1029 ZY= 0.9108

XZ= 0.4159 YZ= -1.2951 ZZ= 22.4051

Eigenvalues: 22.3981 26.2773 29.1712

44 H Isotropic = 25.3608 Anisotropy = 4.7427

XX= 25.6892 YX= -1.8838 ZX= -2.9021

XY= -0.9792 YY= 24.1311 ZY= -0.2393

XZ= -0.4546 YZ= 2.8540 ZZ= 26.2619

Eigenvalues: 23.2380 24.3217 28.5225

45 H Isotropic = 28.4305 Anisotropy = 6.7427

XX= 26.9910 YX= -0.0111 ZX= -0.8652

XY= 1.8170 YY= 25.7954 ZY= 0.9359

XZ= -2.2152 YZ= 0.2961 ZZ= 32.5051

Eigenvalues: 25.0778 27.2881 32.9256

46 H Isotropic = 27.5874 Anisotropy = 10.0187

XX= 27.5627 YX= -2.2571 ZX= -1.5234

XY= -4.2544 YY= 28.4015 ZY= 4.5569

XZ= -1.8426 YZ= 4.7963 ZZ= 26.7980

Eigenvalues: 22.6703 25.8254 34.2665

47 H Isotropic = 27.5592 Anisotropy = 4.1254

XX= 30.1206 YX= -0.3989 ZX= 0.0851

XY= 0.1051 YY= 24.5074 ZY= 0.2417

XZ= 1.1665 YZ= -1.3286 ZZ= 28.0495

Eigenvalues: 24.4254 27.9426 30.3095

48 H Isotropic = 28.0181 Anisotropy = 4.4680

XX= 26.7913 YX= 3.1695 ZX= 0.3996

XY= 3.1409 YY= 28.0448 ZY= -0.3236

XZ= -0.5111 YZ= 2.4466 ZZ= 29.2183

Eigenvalues: 24.0990 28.9586 30.9968

49 H Isotropic = 28.7429 Anisotropy = 17.7107

XX= 23.1856 YX= -1.3331 ZX= 3.9214

XY= -1.4193 YY= 35.0771 ZY= 8.2046

XZ= 2.1414 YZ= 8.3583 ZZ= 27.9660

Eigenvalues: 19.5357 26.1429 40.5500

50 H Isotropic = 31.3560 Anisotropy = 20.7837

XX= 34.5616 YX= 4.8321 ZX= -7.0956

XY= 5.2231 YY= 23.5001 ZY= -3.1199

XZ= -9.4134 YZ= -3.8240 ZZ= 36.0064

Eigenvalues: 21.5490 27.3073 45.2118

4) Briarane B-3

C1

SCF GIAO Magnetic shielding tensor (ppm):

1 C Isotropic = 56.0831 Anisotropy = 128.4010

XX= -36.3403 YX= 4.7215 ZX= -24.7532

XY= 6.3781 YY= 136.5094 ZY= 19.3677

XZ= -23.9806 YZ= 19.6447 ZZ= 68.0801

Eigenvalues: -42.2747 68.8402 141.6837

2 C Isotropic = -21.2844 Anisotropy = 185.7016

XX= -101.0237 YX= -31.2988 ZX= 6.9481

XY= -33.7958 YY= 96.8947 ZY= 3.9024

XZ= 2.8913 YZ= 14.1240 ZZ= -59.7243

Eigenvalues: -107.0781 -59.2919 102.5166

3 C Isotropic = 134.7964 Anisotropy = 31.8054

XX= 142.2317 YX= -5.1620 ZX= 12.4082

XY= -5.8123 YY= 124.5822 ZY= -1.5976

XZ= 17.0754 YZ= -4.2236 ZZ= 137.5752

Eigenvalues: 122.6540 125.7352 156.0000

4 C Isotropic = 24.8741 Anisotropy = 174.2869

XX= -97.4910 YX= 8.0909 ZX= 13.2436

XY= -0.8413 YY= 129.1924 ZY= 33.4721

XZ= 28.5809 YZ= 33.3669 ZZ= 42.9210

Eigenvalues: -100.5462 34.1032 141.0654

5 C Isotropic = 96.7639 Anisotropy = 20.7191

XX= 90.7316 YX= 5.9191 ZX= -4.0524

XY= 8.4478 YY= 89.8307 ZY= 1.5973

XZ= -1.5814 YZ= -5.2570 ZZ= 109.7293

Eigenvalues: 83.0699 96.6450 110.5766

6 C Isotropic = 110.0015 Anisotropy = 37.1438

XX= 123.1360 YX= -7.4860 ZX= 3.0962

XY= -9.5802 YY= 72.3968 ZY= 4.0474

XZ= -1.6016 YZ= 4.4518 ZZ= 134.4718

Eigenvalues: 70.7082 124.5323 134.7640

7 C Isotropic = 105.2940 Anisotropy = 54.3013

XX= 133.3286 YX= -8.0328 ZX= 20.0012

XY= -2.9884 YY= 99.0681 ZY= -2.9503

XZ= 19.9107 YZ= -7.9484 ZZ= 83.4852

Eigenvalues: 75.9871 98.3999 141.4949

8 C Isotropic = 106.1479 Anisotropy = 42.3630

XX= 122.6698 YX= 4.1479 ZX= -22.9115

XY= 12.6950 YY= 81.9924 ZY= 19.4821

XZ= -8.0156 YZ= 8.9161 ZZ= 113.7815

Eigenvalues: 72.7703 111.2834 134.3899

9 C Isotropic = 35.9113 Anisotropy = 167.1224

XX= -59.8072 YX= -14.5646 ZX= 44.3994

XY= -13.2266 YY= 145.8495 ZY= -2.1954

XZ= 44.2532 YZ= -7.7632 ZZ= 21.6916

Eigenvalues: -79.7800 40.1876 147.3262

10 C Isotropic = 150.2618 Anisotropy = 20.3683

XX= 149.4706 YX= -4.0061 ZX= -9.5766

XY= -2.6016 YY= 147.7894 ZY= -0.4304

XZ= -14.6071 YZ= -8.0596 ZZ= 153.5255

Eigenvalues: 136.7230 150.2219 163.8407

11 C Isotropic = 152.8700 Anisotropy = 23.9848

XX= 157.5630 YX= -10.5669 ZX= -1.9364

XY= -14.3517 YY= 145.8972 ZY= -11.4029

XZ= 1.5243 YZ= -11.5363 ZZ= 155.1499

Eigenvalues: 133.3428 156.4073 168.8599

12 C Isotropic = 102.9296 Anisotropy = 42.8319

XX= 122.8374 YX= 1.5241 ZX= -15.1885

XY= 1.1151 YY= 96.6229 ZY= -17.6301

XZ= -16.6765 YZ= -19.6308 ZZ= 89.3284

Eigenvalues: 71.2828 106.0217 131.4842

13 C Isotropic = 137.9543 Anisotropy = 15.9395

XX= 140.2662 YX= -0.9552 ZX= 0.7136

XY= 5.2491 YY= 148.0145 ZY= 0.5290

XZ= -0.9808 YZ= 0.5799 ZZ= 125.5823

Eigenvalues: 125.5662 139.7161 148.5807

14 C Isotropic = 144.4065 Anisotropy = 18.4900

XX= 154.7531 YX= 3.1977 ZX= -1.9438

XY= 0.3257 YY= 129.4268 ZY= -6.2524

XZ= -4.6510 YZ= -2.9398 ZZ= 149.0397

Eigenvalues: 128.3642 148.1222 156.7332

15 O Isotropic = -316.8906 Anisotropy = 978.8709

XX= -369.4698 YX= -124.9091 ZX= 87.2464

XY= -131.4783 YY= 306.5962 ZY= 88.2097

XZ= 67.4332 YZ= 107.6435 ZZ= -887.7982

Eigenvalues: -910.2512 -376.1106 335.6900

16 C Isotropic = 170.8555 Anisotropy = 23.8400

XX= 160.4478 YX= -1.7333 ZX= -1.9584

XY= 3.2862 YY= 185.8591 ZY= 5.4222

XZ= -1.7831 YZ= 3.0893 ZZ= 166.2594

Eigenvalues: 159.7400 166.0775 186.7488

17 C Isotropic = 170.1036 Anisotropy = 21.5938

XX= 166.0011 YX= -1.8336 ZX= -2.2336

XY= -0.7481 YY= 167.2324 ZY= 12.5091

XZ= -2.3716 YZ= 9.3115 ZZ= 177.0774

Eigenvalues: 160.1806 165.6307 184.4995

18 H Isotropic = 28.8166 Anisotropy = 6.5042

XX= 29.3130 YX= -1.0878 ZX= -2.0904

XY= -2.5021 YY= 31.8224 ZY= -0.3948

XZ= -2.0128 YZ= 2.1166 ZZ= 25.3143

Eigenvalues: 24.4476 28.8494 33.1527

19 C Isotropic = 138.4501 Anisotropy = 26.4190

XX= 149.2139 YX= -2.5313 ZX= 16.6508

XY= 2.4203 YY= 138.1105 ZY= -15.1694

XZ= 7.0223 YZ= -7.9692 ZZ= 128.0259

Eigenvalues: 117.2494 142.0382 156.0628

20 C Isotropic = 6.2556 Anisotropy = 80.2759

XX= 49.1320 YX= -43.0941 ZX= 18.9602

XY= -27.7426 YY= -63.6800 ZY= 41.0595

XZ= 9.8952 YZ= 42.5994 ZZ= 33.3148

Eigenvalues: -89.7492 48.7431 59.7729

21 O Isotropic = 76.2258 Anisotropy = 217.7736

XX= -81.8916 YX= 120.2262 ZX= -69.7800

XY= 42.1305 YY= 193.7026 ZY= -11.8591

XZ= -43.3673 YZ= -5.2652 ZZ= 116.8665

Eigenvalues: -115.8734 123.1426 221.4082

22 O Isotropic = -82.9688 Anisotropy = 594.8565

XX= -325.2044 YX= -76.9768 ZX= -47.0162

XY= -103.1906 YY= -163.7361 ZY= 181.5430

XZ= 8.9378 YZ= 181.4274 ZZ= 240.0341

Eigenvalues: -371.9692 -190.5393 313.6022

23 C Isotropic = 177.5937 Anisotropy = 10.8261

XX= 178.8910 YX= 0.9964 ZX= -2.3350

XY= 3.3988 YY= 174.9374 ZY= -4.6103

XZ= -1.6510 YZ= -7.6196 ZZ= 178.9528

Eigenvalues: 170.4610 177.5091 184.8112

24 H Isotropic = 26.5303 Anisotropy = 5.7652

XX= 29.1406 YX= -1.8311 ZX= -1.6191

XY= -1.0327 YY= 25.7109 ZY= 1.1430

XZ= -2.0438 YZ= 0.5178 ZZ= 24.7394

Eigenvalues: 24.0077 25.2095 30.3737

25 Cl Isotropic = 806.6471 Anisotropy = 320.7298

XX= 812.7650 YX= -28.1405 ZX= -126.7159

XY= -41.5878 YY= 738.5474 ZY= 57.1222

XZ= -186.6365 YZ= 83.0517 ZZ= 868.6288

Eigenvalues: 674.3542 725.1201 1020.4669

26 C Isotropic = 56.1022 Anisotropy = 158.6465

XX= -28.8715 YX= -17.5144 ZX= 44.1676

XY= -25.1070 YY= 154.5051 ZY= -28.3318

XZ= 47.2201 YZ= -7.8956 ZZ= 42.6731

Eigenvalues: -51.7582 58.1983 161.8665

27 O Isotropic = 267.4238 Anisotropy = 48.9815

XX= 281.2367 YX= -10.7157 ZX= -25.2821

XY= -10.8874 YY= 293.5289 ZY= 9.1360

XZ= 15.2702 YZ= -4.7915 ZZ= 227.5059

Eigenvalues: 227.0222 275.1711 300.0781

28 O Isotropic = 79.1535 Anisotropy = 145.9678

XX= 70.8795 YX= 13.3556 ZX= 64.3718

XY= 141.1562 YY= 84.9153 ZY= -106.5299

XZ= 122.3240 YZ= -71.5352 ZZ= 81.6658

Eigenvalues: -94.2457 155.2409 176.4654

29 C Isotropic = 8.2420 Anisotropy = 93.7748

XX= -57.7520 YX= -44.8768 ZX= 23.1505

XY= -73.5881 YY= 19.3176 ZY= -1.4500

XZ= 5.4858 YZ= -9.4265 ZZ= 63.1605

Eigenvalues: -90.5351 44.5026 70.7586

30 O Isotropic = -106.2475 Anisotropy = 579.7655

XX= -176.3699 YX= -113.4597 ZX= 108.3693

XY= -99.1846 YY= -251.4884 ZY= -271.5775

XZ= 114.9373 YZ= -216.5365 ZZ= 109.1158

Eigenvalues: -386.0400 -212.9653 280.2628

31 C Isotropic = 164.5819 Anisotropy = 46.8824

XX= 189.6566 YX= 9.1944 ZX= -14.9403

XY= 0.9553 YY= 151.6726 ZY= 4.9182

XZ= -16.9144 YZ= 1.7528 ZZ= 152.4166

Eigenvalues: 143.5139 154.3951 195.8369

32 O Isotropic = 102.9544 Anisotropy = 140.1040

XX= 183.9176 YX= -24.2272 ZX= 67.0697

XY= -21.3730 YY= 121.1565 ZY= 183.2859

XZ= 25.0158 YZ= 39.9014 ZZ= 3.7892

Eigenvalues: -73.7851 186.2912 196.3571

33 C Isotropic = 8.9788 Anisotropy = 90.5548

XX= 65.1322 YX= -13.8584 ZX= -27.9046

XY= -16.0997 YY= 12.0471 ZY= -81.1478

XZ= -13.0370 YZ= -46.1207 ZZ= -50.2428

Eigenvalues: -93.9678 51.5556 69.3487

34 O Isotropic = -107.4238 Anisotropy = 594.8944

XX= 224.3123 YX= -199.2768 ZX= 7.5436

XY= -199.5687 YY= -326.5742 ZY= -9.0421

XZ= -4.5631 YZ= -54.4377 ZZ= -220.0094

Eigenvalues: -396.2107 -215.2331 289.1725

35 C Isotropic = 163.8912 Anisotropy = 46.5420

XX= 145.1518 YX= 3.6641 ZX= -0.1366

XY= 3.8746 YY= 151.9413 ZY= -0.6254

XZ= 2.7167 YZ= 7.6506 ZZ= 194.5804

Eigenvalues: 143.4726 153.2818 194.9192

36 H Isotropic = 25.6261 Anisotropy = 4.7351

XX= 24.3210 YX= 0.0812 ZX= -0.0831

XY= -0.2283 YY= 24.3969 ZY= -1.7238

XZ= -0.4403 YZ= -1.5477 ZZ= 28.1605

Eigenvalues: 23.7402 24.3553 28.7829

37 H Isotropic = 29.1357 Anisotropy = 4.7734

XX= 28.2376 YX= -3.4565 ZX= -3.5209

XY= -4.0707 YY= 28.7818 ZY= -2.3121

XZ= -1.3627 YZ= -2.5407 ZZ= 30.3876

Eigenvalues: 23.1096 31.9795 32.3179

38 H Isotropic = 25.0315 Anisotropy = 8.2288

XX= 23.4780 YX= -0.8563 ZX= 2.7719

XY= -1.1184 YY= 25.4647 ZY= -2.8890

XZ= 4.0656 YZ= -3.4442 ZZ= 26.1519

Eigenvalues: 20.8579 23.7192 30.5174

39 H Isotropic = 26.2425 Anisotropy = 3.2510

XX= 27.8657 YX= -0.0265 ZX= 1.1860

XY= -1.6860 YY= 26.2746 ZY= 0.5667

XZ= 0.5131 YZ= -0.6980 ZZ= 24.5872

Eigenvalues: 24.3688 25.9488 28.4098

40 H Isotropic = 26.4677 Anisotropy = 6.1337

XX= 24.6980 YX= -1.1305 ZX= -2.4884

XY= -1.7222 YY= 26.5601 ZY= -2.9711

XZ= -2.4699 YZ= -2.6962 ZZ= 28.1450

Eigenvalues: 21.8450 27.0012 30.5568

41 H Isotropic = 28.4951 Anisotropy = 8.3058

XX= 30.7566 YX= 3.0861 ZX= -0.9690

XY= 3.5450 YY= 30.4104 ZY= 0.3811

XZ= -1.7905 YZ= -0.7049 ZZ= 24.3181

Eigenvalues: 23.9823 27.4706 34.0323

42 H Isotropic = 29.8613 Anisotropy = 7.4811

XX= 28.9198 YX= -2.0376 ZX= -0.3983

XY= -2.5532 YY= 30.5667 ZY= -4.3283

XZ= 0.4448 YZ= -3.6844 ZZ= 30.0972

Eigenvalues: 25.5280 29.2071 34.8487

43 H Isotropic = 30.2629 Anisotropy = 6.8234

XX= 29.6777 YX= -0.7258 ZX= -2.8082

XY= -1.3067 YY= 27.8929 ZY= -1.3676

XZ= -2.7937 YZ= -1.0687 ZZ= 33.2182

Eigenvalues: 26.5089 29.4680 34.8118

44 H Isotropic = 28.9914 Anisotropy = 8.5537

XX= 31.9074 YX= 3.7490 ZX= 0.7291

XY= 2.7509 YY= 30.7741 ZY= -0.1280

XZ= 1.0103 YZ= 0.3876 ZZ= 24.2928

Eigenvalues: 24.1842 28.0962 34.6939

45 H Isotropic = 26.8590 Anisotropy = 3.3306

XX= 28.6947 YX= 0.9210 ZX= -0.9999

XY= 0.0893 YY= 28.3770 ZY= -0.1331

XZ= -0.5732 YZ= 1.4122 ZZ= 23.5052

Eigenvalues: 23.2898 28.2077 29.0793

46 H Isotropic = 29.7654 Anisotropy = 6.2640

XX= 28.9605 YX= 1.0387 ZX= 4.7611

XY= 2.7502 YY= 31.4043 ZY= 0.7170

XZ= 3.7058 YZ= -0.7344 ZZ= 28.9315

Eigenvalues: 24.4453 30.9096 33.9414

47 H Isotropic = 30.7015 Anisotropy = 11.8652

XX= 26.3770 YX= 1.1683 ZX= -0.9054

XY= 2.1059 YY= 34.4725 ZY= -5.2821

XZ= -1.5325 YZ= -5.0430 ZZ= 31.2551

Eigenvalues: 26.0365 27.4565 38.6117

48 H Isotropic = 30.8554 Anisotropy = 6.3454

XX= 31.7219 YX= -2.2386 ZX= -2.0836

XY= -2.3192 YY= 33.2077 ZY= 0.4443

XZ= -2.9889 YZ= -1.1608 ZZ= 27.6366

Eigenvalues: 26.1605 31.3201 35.0857

49 H Isotropic = 30.3819 Anisotropy = 6.2734

XX= 31.1006 YX= -0.6228 ZX= 1.5374

XY= -1.1205 YY= 26.8079 ZY= 1.7462

XZ= 2.7025 YZ= 0.2620 ZZ= 33.2373

Eigenvalues: 26.3248 30.2568 34.5642

50 H Isotropic = 30.5978 Anisotropy = 10.3469

XX= 34.9456 YX= -1.5016 ZX= -3.3999

XY= -2.3544 YY= 28.8067 ZY= 2.9452

XZ= -4.0788 YZ= 2.1189 ZZ= 28.0411

Eigenvalues: 25.4908 28.8068 37.4957

51 H Isotropic = 30.6915 Anisotropy = 6.3212

XX= 29.0599 YX= 1.2129 ZX= -1.3787

XY= 1.5504 YY= 34.5083 ZY= 0.9377

XZ= -1.5324 YZ= 1.0574 ZZ= 28.5063

Eigenvalues: 26.9393 30.2295 34.9056

52 H Isotropic = 29.4484 Anisotropy = 5.5459

XX= 31.5648 YX= -0.2202 ZX= -1.5267

XY= -0.2803 YY= 27.0886 ZY= 3.1661

XZ= -1.1759 YZ= 3.8447 ZZ= 29.6919

Eigenvalues: 24.6036 30.5960 33.1457

53 H Isotropic = 30.2959 Anisotropy = 11.5146

XX= 35.8915 YX= 4.0206 ZX= 1.3409

XY= 4.7344 YY= 28.4208 ZY= -0.4635

XZ= 0.8887 YZ= -0.3832 ZZ= 26.5753

Eigenvalues: 25.6067 27.3087 37.9722

54 H Isotropic = 30.8693 Anisotropy = 7.9471

XX= 31.0360 YX= -1.1198 ZX= -0.9265

XY= -1.0851 YY= 34.2502 ZY= -3.6741

XZ= -0.6806 YZ= -4.3268 ZZ= 27.3216

Eigenvalues: 25.2479 31.1925 36.1674

55 H Isotropic = 30.7855 Anisotropy = 8.4151

XX= 30.5298 YX= 1.1205 ZX= -3.7943

XY= 1.2174 YY= 28.1971 ZY= 2.5419

XZ= -3.8470 YZ= 1.9533 ZZ= 33.6297

Eigenvalues: 25.7809 30.1802 36.3956

56 H Isotropic = 25.4108 Anisotropy = 6.7276

XX= 23.6867 YX= 0.4723 ZX= 0.6445

XY= 0.6611 YY= 26.4721 ZY= 3.0291

XZ= 2.9652 YZ= 3.3108 ZZ= 26.0734

Eigenvalues: 22.2686 24.0678 29.8958

57 H Isotropic = 25.4396 Anisotropy = 7.0686

XX= 26.4668 YX= 0.4320 ZX= 5.0496

XY= 0.8417 YY= 24.9443 ZY= 2.3253

XZ= 2.1351 YZ= 2.3937 ZZ= 24.9078

Eigenvalues: 21.3472 24.8197 30.1521

58 H Isotropic = 28.3548 Anisotropy = 22.9424

XX= 20.0550 YX= -8.1885 ZX= -4.0279

XY= -6.3109 YY= 39.6138 ZY= 4.5529

XZ= -2.9852 YZ= 4.6181 ZZ= 25.3956

Eigenvalues: 17.1529 24.2617 43.6497

59 H Isotropic = 29.5407 Anisotropy = 6.5702

XX= 32.7695 YX= -2.4137 ZX= 0.5361

XY= -1.4725 YY= 29.6978 ZY= -1.9334

XZ= 0.4857 YZ= -1.6763 ZZ= 26.1547

Eigenvalues: 25.3853 29.3160 33.9208

60 H Isotropic = 29.6410 Anisotropy = 6.4043

XX= 30.0543 YX= 0.8776 ZX= -2.7468

XY= 1.7981 YY= 26.2181 ZY= 0.8329

XZ= -1.6334 YZ= 1.3670 ZZ= 32.6506

Eigenvalues: 25.3621 29.6504 33.9105

61 H Isotropic = 29.8010 Anisotropy = 9.0509

XX= 32.6372 YX= 5.5757 ZX= -0.3174

XY= 2.5605 YY= 30.6064 ZY= 1.3225

XZ= -1.8800 YZ= 0.0686 ZZ= 26.1593

Eigenvalues: 25.4014 28.1666 35.8349

62 H Isotropic = 29.6899 Anisotropy = 9.9874

XX= 26.0181 YX= 2.0946 ZX= 0.9403

XY= 2.0986 YY= 30.7562 ZY= 2.4477

XZ= 1.9754 YZ= 5.8769 ZZ= 32.2956

Eigenvalues: 25.2205 27.5011 36.3482

63 H Isotropic = 29.5610 Anisotropy = 6.0784

XX= 27.7356 YX= -2.3970 ZX= 2.6953

XY= -2.4703 YY= 29.2411 ZY= 0.0906

XZ= 2.5176 YZ= -1.1118 ZZ= 31.7062

Eigenvalues: 25.3716 29.6980 33.6133

64 H Isotropic = 29.5910 Anisotropy = 5.0833

XX= 30.9313 YX= 1.9378 ZX= -1.2017

XY= 1.8867 YY= 26.1517 ZY= 1.6976

XZ= -2.0280 YZ= 0.7331 ZZ= 31.6899

Eigenvalues: 25.0428 30.7503 32.9798
